# Spironolactone and Fibrosis in Heart Failure Risk: Machine Learning Analysis of HOMAGE Trial Plasma Proteomics

**DOI:** 10.1002/mco2.70634

**Published:** 2026-02-17

**Authors:** Susana Ravassa, Nicolas Girerd, Frank Edelman, Begoña López, João Pedro Ferreira, Daniela Zurkan, Gorka San José, Iñigo Latasa, Pierpaolo Pellicori, Franco Cosmi, Johannes Petutschnigg, Stephane Heymans, Hans‐Peter Brunner‐La Rocca, Burkert Pieske, Christian Delles, Andrew L. Clark, Javier Díez, Faiez Zannad, John G. F. Cleland, Arantxa González, the HOMAGE Trial Committees and Investigators

**Affiliations:** ^1^ Laboratory of Heart Failure CIMA Universidad de Navarra and IdiSNA Pamplona Spain; ^2^ CIBERCV, Carlos III Institute of Health Madrid Spain; ^3^ Inserm, Centre d'Investigation Clinique Plurithématique 1433, U1116, CHRU De Nancy, F‐CRIN INI‐CRCT Université De Lorraine Nancy France; ^4^ Department of Internal Medicine and Cardiology Campus Virchow Klinikum Charité University Medicine Berlin, Germany; ^5^ DZHK (German Centre for Cardiovascular Research), Partner Site Berlin Berlin Germany; ^6^ Cardiovascular Research and Development Center, Department of Surgery and Physiology Faculty of Medicine of the University of Porto Porto Portugal; ^7^ School of Cardiovascular and Metabolic Health University of Glasgow Glasgow UK; ^8^ Department of Cardiology Cortona Hospital Arezzo Italy; ^9^ Deutsches Herzzentrum der Charité, Klinik für Kardiologie Angiologie & Intensivmedizin, Augustenburger Platz 1 Berlin Germany; ^10^ Charité – Universitätsmedizin Berlin Corporate Member of Freie Universität Berlin and Humboldt‐Universität zu Berlin Berlin Germany; ^11^ Department of Cardiology Maastricht University Medical Center Maastricht the Netherlands; ^12^ Division of Cardiology, Department of Internal Medicine University Medicine Rostock Rostock Germany; ^13^ Castle Hill Hospital Hull University Teaching Hospitals NHS Trust Cottingham UK; ^14^ Department of Cardiology and Cardiac Surgery Clínica Universidad de Navarra Pamplona Spain

**Keywords:** biomarkers, fibrosis, heart failure, machine learning algorithms (MLA), procollagen Type I C‐terminal propeptide (PICP), spironolactone

## Abstract

In the HOMAGE (Heart Omics in AGEing) trial, spironolactone reduced serum concentrations of procollagen Type I C‐terminal propeptide (PICP), a fibrosis biomarker, in patients at risk of heart failure. To elucidate the underlying mechanisms, multidimensional analyses including proteomics were conducted. Olink cardiovascular and inflammation panels (*n* = 276 proteins) were measured in plasma from 488 HOMAGE participants at baseline, 1 month, and 9 months after randomization. Proteins associated with PICP changes were identified using machine learning algorithms (MLAs). Selected candidates were further analyzed in patients with heart failure and preserved ejection fraction (Aldo‐DHF trial). Linear regression and mediation analyses assessed which MLA‐selected proteins mediated spironolactone's effects on PICP. MLAs consistently linked PICP reduction to changes in biomarkers of collagen (e.g., decreased COL1A1), fatty acid metabolism (e.g., increased FABP4), immune function (e.g., increased CCL24 and IL6RA, and decreased FLT3L), neurological function (e.g., increased DNER), cell–matrix interactions (e.g., increased galectin‐9 [GAL9] and decreased thrombospondin‐2 [THBS2]), and reduced NT‐proBNP. Mediation analysis suggested that changes in GAL9 and THBS2 were associated with spironolactone‐induced PICP reduction, which was confirmed in Aldo‐DHF patients. This study raises the hypothesis that spironolactone inhibits collagen synthesis via inflammatory, metabolic, and extracellular matrix pathways, and particularly through modulation of GAL9 and THBS2.

## Graphical abstract

In HOMAGE, 276 plasma proteins were profiled using Olink panels. Machine learning algorithms identified galectin‐9 (GAL9), thrombospondin‐2 (THBS2), and NT‐proBNP as predictors of changes in procollagen type I C‐terminal propeptide (PICP) after spironolactone treatment. Decreasing THBS2 and increasing GAL9 were associated with PICP reduction in HOMAGE and Aldo‐DHF patients, suggesting a potential mediating role of these proteins in fibrosis modulation

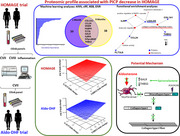

## 1 Introduction

Cardiac fibrosis is a key feature of myocardial remodeling, contributes to the development and progression of heart failure, and is associated with adverse cardiovascular (CV) outcomes [1]. The renin [REN]–angiotensin–aldosterone system is an important trigger for cardiac fibrosis; mineralocorticoid receptor antagonists (MRAs) exert antifibrotic effects in clinical and experimental studies [2] but the molecular mechanisms involved are uncertain.

In the Heart OMics in AGEing (HOMAGE) randomized trial investigating patients at risk of heart failure, treatment with spironolactone reduced serum concentrations of procollagen Type I C‐terminal propeptide (PICP) [3], a peptide related to collagen Type I synthesis that is associated with the extent of myocardial fibrosis [1]. The effect on collagen metabolism was observed within 1 month of initiating spironolactone and was maintained until the end of the trial (9 months or last visit). Similarly, in patients with heart failure and preserved ejection fraction (HFpEF) from the Aldosterone Receptor Blockade in Diastolic Heart Failure (Aldo‐DHF) trial, spironolactone also reduced serum PICP [4]. Given the pleiotropic actions of MR blockade by spironolactone, identifying potential pathways involved in its antifibrotic effects could be useful in clarifying its mechanisms of action leading to discovery of novel therapeutic targets.

The plasma proteome is a dynamic summary of the complex biological pathways in health and disease. In the absence of direct access to cardiac tissue, the plasma proteome may be a useful reflection of molecular changes in the myocardium associated with disease progression and therapy [5]. Although exploratory in nature, the development of high throughput targeted technologies, like the proximity extension assay (PEA), has overcome classical limitations of unbiased plasma proteomic analysis and opened new avenues of research. Measuring an array of proteins should provide a more comprehensive tool for generating hypothesis about molecular pathways than analysis of single biomarkers [6].

Therefore, we attempted to unravel potential downstream pathways associated with the antifibrotic effects of spironolactone‐induced MR blockade in the HOMAGE trial by analyzing the association of changes in serum concentrations of PICP compared with 276 plasma proteins, measured using targeted PEA panels (Olink).

## 2 Results

### 2.1 HOMAGE: Baseline Characteristics by PICP‐Change Quartiles

Baseline characteristics of patients classified by quartiles of change in serum PICP concentration both after 1 (*n* = 481) and 9 (*n* = 488) months of treatment are shown in Table 1. One month after randomization, higher baseline PICP, age, and *E*:*e*′ ratio, female sex, and treatment with spironolactone were associated with a greater reduction of serum PICP (lowest quartile). Except for baseline PICP, and spironolactone, these trends were not observed at 9 months.

**TABLE 1:** Baseline characteristics by quartiles of the PICP fold‐change at 1 and 9 months (HOMAGE trial).

	Classification by PICP changes at 1 month				*p* Trend	Classification by PICP changes at 9 months				*p* Trend
	Q1 (*n* = 121)	Q2 (*n* = 120)	Q3 (*n* = 120)	Q4 (*n* = 120)		Q1 (*n* = 122)	Q2 (*n* = 122)	Q3 (*n* = 122)	Q4 (*n* = 122)	
PICP change (final/baseline)	<0.86	0.86–0.97	0.97–1.12	>1.12		<0.81	0.81–0.96	0.96–1.12	>1.12	
Demographics										
Age, years	74 (69–79)	73 (68–79)	71 (67–77)	71 (68–75)	**0.009**	72 (67–78)	72 (68–78)	73 (69–77)	72 (68–78)	0.378
Men, *n* (%)	78 (64.5)	93 (77.5)	95 (79.2)	95 (79.2)	**0.009**	89 (73.0)	86 (70.5)	96 (78.7)	96 (78.7)	0.146
Current smoker, *n* (%)	11 (9.1)	10 (8.3)	10 (8.3)	9 (7.5)	0.641	14 (11.5)	8 (6.6)	7 (5.7)	7 (5.7)	0.091
Prior medical history, *n* (%)										
Hypertension	101 (83.5)	91 (75.8)	87 (72.5)	96 (80.0)	0.413	97 (79.5)	93 (76.2)	94 (77.0)	97 (79.5)	0.961
Diabetes mellitus	49 (40.5)	55 (45.8)	45 (37.5)	41 (34.2)	0.171	49 (40.2)	47 (38.5)	50 (41.0)	47 (38.5)	0.901
Coronary artery disease	86 (71.1)	87 (72.5)	87 (72.5)	90 (75.0)	0.516	78 (63.9)	91 (74.6)	97 (79.5)	88 (72.1)	0.102
Myocardial infarction	48 (39.7)	47 (39.2)	52 (43.3)	49 (40.8)	0.702	42 (34.4)	48 (39.3)	57 (46.7)	55 (45.1)	0.049
PCI	56 (46.3)	59 (49.2)	60 (50.0)	70 (58.3)	0.070	49 (40.2)	61 (50.0)	74 (60.7)	66 (54.1)	**0.010**
CABG	31 (25.6)	35 (29.2)	31 (25.8)	34 (28.3)	0.790	32 (26.2)	36 (29.5)	35 (28.7)	27 (22.1)	0.464
STIA	7 (5.8)	5 (4.2)	6 (5.0)	5 (4.2)	0.643	2 (1.6)	8 (6.6)	6 (4.9)	5 (4.1)	0.485
COPD	6 (5.0)	9 (7.5)	7 (5.8)	9 (7.5)	0.551	8 (6.6)	2 (1.6)	11 (9.0)	8 (6.6)	0.441
Randomized treatment, n (%)										
Spironolactone	72 (59.5)	69 (57.5)	47 (39.2)	51 (42.5)	**0.001**	78 (63.9)	59 (48.4)	59 (48.4)	50 (41.0)	**0.001**
Other baseline medications, *n* (%)										
ACE inhibitor	68 (56.2)	68 (56.7)	56 (46.7)	59 (49.2)	0.127	65 (53.3)	65 (53.3)	62 (50.8)	62 (50.8)	0.627
ARB	32 (26.4)	22 (18.3)	39 (32.5)	38 (31.7)	0.101	34 (27.9)	31 (25.4)	36 (29.5)	34 (27.9)	0.821
Beta‐blockers	84 (69.4)	82 (68.3)	86 (71.7)	81 (67.5)	0.897	80 (65.6)	85 (69.7)	93 (76.2)	84 (68.9)	0.377
Thiazide diuretics	22 (18.2)	19 (15.8)	15 (12.5)	22 (18.3)	0.846	22 (18.0)	17 (13.9)	19 (15.6)	23 (18.9)	0.786
Calcium channel blockers	26 (21.5)	27 (22.5)	22 (18.3)	27 (22.5)	0.946	24 (19.7)	23 (18.9)	28 (23.0)	26 (21.3)	0.583
Lipid‐lowering therapy	99 (81.8)	103 (85.8)	99 (82.5)	94 (78.3)	0.379	97 (79.5)	103 (84.4)	105 (86.1)	98 (80.3)	0.790
Aspirin	80 (66.1)	82 (68.3)	89 (74.2)	93 (77.5)	0.030	75 (61.5)	91 (74.6)	89 (73.0)	90 (73.8)	0.056
Any antiplatelet therapy	93 (76.9)	94 (78.3)	96 (80.0)	96 (80.0)	0.505	86 (70.5)	100 (82.0)	99 (81.1)	95 (77.9)	0.205
Physical exam										
BMI, kg/m^2^	28.4 (26.1–31.6)	28.2 (25.0–31.9)	28.3 (25.4–31.6)	27.3 (25.0–30.8)	0.044	28.2 (25.8–31.4)	28.1 (25.3–31.7)	27.8 (24.9–31.1)	28.4 (25.4–32.1)	0.953
HR, beats/min	60.0 (54.5–66.0)	60.0 (55.0–66.0)	59.0 (54.5–68.5)	61.0 (54.0–68.0)	0.385	60.5 (54.0–67.0)	60.0 (55.0–67.0)	60.0 (54.0–66.0)	61.0 (55.0–69.0)	0.510
SBP, mmHg	141 (131–154)	140 (128–156)	140 (125–159)	140 (127–150)	0.262	142 (129–156)	141 (127–151)	139 (127–153)	141 (128–156)	0.561
DBP, mmHg	78 (72–84)	78 (72–83)	76 (71–85)	80 (72–87)	0.437	78 (72–84)	78 (71–83)	77 (70–84)	79 (71–87)	0.465
Breathlessness scale	5.0 (3.0–6.0)	5.0 (3.0–6.2)	5.5 (4.0–7.0)	5.0 (3.0–6.0)	0.571	5.0 (3.0–7.0)	5.0 (3.0–6.0)	5.0 (4.0–6.0)	5.0 (4.0–7.0)	0.249
Baseline blood tests										
Hemoglobin, g/dL	13.8 (12.9–14.7)	14.0 (13.3–14.9)	14.2 (13.3–14.9)	14.1 (13.1–14.9)	0.114	13.9 (13.1–14.7)	14.0 (12.9–15.0)	14.3 (13.4–15.1)	13.9 (12.9–14.8)	0.602
Sodium, mmol/L	139 (138–141)	140 (138–141)	139 (138–141)	140 (138–141)	0.979	140 (138–142)	139 (137–141)	140 (138–141)	139 (138–141)	0.282
Potassium, mmol/L	4.3 (4.1–4.7)	4.3 (4.0–4.5)	4.4 (4.2–4.6)	4.3 (4.1–4.5)	0.275	4.3 (4.1–4.6)	4.3 (4.1–4.5)	4.3 (4.1–4.5)	4.3 (4.1–4.6)	0.995
eGFR, mL/min/1.73 m^2^	72 (61–4)	75 (63–86)	74 (61–83)	77 (65–88)	0.255	76 (63–86)	72 (59–87)	75 (63–86)	74 (62–84)	0.725
Electrocardiography										
QRS	92 (84–102)	96 (84–110)	92 (82–106)	90 (84–106)	0.991	90 (84–100)	90 (82–102)	96 (86–110)	96 (84–110)	0.032
Echocardiography										
LVEDVi, mL/m^2^	40.1 (34.7–47.1)	41.9 (35.2–48.7)	42.1 (35.7–48.7)	43.1 (37.4–50.0)	0.041	41.2 (34.8–49.3)	41.8 (36.9–48.0)	43.9 (36.5–48.4)	41.9 (33.7–49.0)	0.858
LVEF, %	63.4 (58.6–66.7)	63.1 (58.7–67.0)	63.5 (58.9–65.7)	62.3 (55.1–66.4)	0.282	61.8 (57.4–67.0)	63.7 (60.3–67.3)	63.5 (59.6–65.9)	61.8 (55.0–66.2)	0.209
LVMI, g/m^2^	94.9 (80.7–112.5)	96.8 (82.6–112.1)	92.0 (80.6–113.6)	92.3 (77.2–109.8)	0.168	95.4 (84.2–113.2)	92.0 (78.3–112.5)	92.8 (79.1–102.7)	96.6 (82.0–114.5)	0.898
LAVI, mL/m^2^	31.3 (26.6–35.6)	30.9 (27.1–36.3)	30.8 (25.5–36.5)	30.1 (25.8–36.0)	0.630	31.1 (26.0–36.8)	30.9 (26.7–35.0)	31.2 (27.0–37.0)	30.0 (24.9–37.1)	0.537
*E*:*A* ratio	0.8 (0.7–1.0)	0.8 (0.7–1.1)	0.8 (0.7–1.0)	0.8 (0.6–1.0)	0.159	0.8 (0.7–1.0)	0.8 (0.7–1.0)	0.9 (0.7–1.0)	0.8 (0.7–0.9)	0.179
*E*:*e*′ ratio	10.0 (8.0–11.6)	9.5 (8.3–12.0)	8.8 (7.4–10.9)	8.9 (6.8–11.1)	**0.004**	9.4 (7.7–11.6)	9.3 (7.5–11.1)	9.1 (7.4–11.3)	9.4 (7.7–11.4)	0.669
TAPSE	22.7 (19.1–26.9)	21.0 (16.7–27.0)	22.3 (16.6–26.2)	21.5 (16.4–26.4)	0.196	21.4 (17.5–25.8)	22.8 (16.7–26.8)	20.9 (15.4–26.9)	22.0 (18.2–26.4)	0.995
Blood biomarkers at baseline										
NT‐proBNP, ng/L	216 (131–387)	230 (137–378)	191 (133–293)	192 (132–299)	0.101	223 (147–384)	184 (132–306)	208 (130–293)	226 (133–338)	0.365
Hs‐TnT, ng/L	13.3 (8.4–18.0)	12.4 (9.1–17.3)	12.4 (9.2–16.0)	12.0 (8.5–17.6)	0.452	11.9 (8.6–15.9)	12.2 (8.4–17.8)	12.8 (8.7–17.1)	13.2 (9.8–19.4)	0.144
PICP, µg/L	90.1 (76.6–106)	77.6 (66.1–98.0)	77.4 (63.1–91.9)	73.2 (58.0–85.1)	**<0.001**	94.3 (76.4–113)	79.9 (70.3–96.5)	75.5 (63.2–87.8)	74.5 (58.9–89.0)	**<0.001**
PIIINP, µg/L	4.3 (3.3–5.4)	4.0 (3.2–4.8)	3.9 (3.0–5.0)	3.6 (3.0–4.6)	**0.001**	4.3 (3.3–5.3)	3.9 (3.0–4.7)	4.0 (3.2–4.9)	3.7 (2.9–4.8)	0.035
Galectin 3, µg/L	16.1 (13.6–20.6)	15.8 (13.4–19.2)	16.4 (13.2–18.6)	15.3 (13.4–19.1)	0.380	16.4 (13.1–20.4)	16.4 (13.6–19.1)	15.3 (13.1–18.6)	16.3 (14.1–20.8)	0.600
GDF‐15, µg/L	1489 (1107–2269)	1473 (972–2119)	1384 (1035–2002)	1380 (946–1944)	0.036	1462 (1107–2134)	1413 (966–2143)	1415 (1045–1946)	1432 (982–2292)	0.610

PICP means procollagen Type I C‐terminal propeptide.

Values are expressed as median (interquartile range) and categorical variables as numbers and percentages. *p* Values ≤ 0.01 are in bold.

Abbreviations: PCI, percutaneous coronary intervention; CABG, coronary artery bypass graft; STIA, stroke/transient ischemic attach; COPD, chronic obstructive pulmonary disease; ACE, angiotensin‐converting enzyme; ARB, angiotensin II receptor blocker; BMI, body mass index; HR, heart rate; SBP, systolic blood pressure; DBP, diastolic blood pressure; eGFR, estimated glomerular filtration rate; LVEDVi, left ventricular (LV) end‐diastolic volume index; LVEF, LV ejection fraction; LVMI, LV mass index; LAVI, left atrial volume index; *E*, early mitral flow velocity; *A*, late (atrial) mitral flow velocity; *e*’, early diastolic tissue velocity; TAPSE: tricuspid annular plane systolic excursion; NT‐proBNP; N‐terminal pro‐brain natriuretic peptide; Hs‐TnT, high sensitivity troponin T; PIIINP, procollagen type III N‐terminal propeptide; GDF‐15, growth differentiation factor 15.

The relative baseline concentrations of all 276 Olink (description of protein names and panels in Table S1) across quartiles of the PICP change at 1 and 9 months are shown in the Table S2. Higher baseline plasma thrombospondin‐2 (THBS2) was associated with a greater reduction in PICP at both 1 and 9 months (Table S2).

### 2.2 HOMAGE: Proteomic and Clinical Predictors of PICP Reduction

**FIGURE 1:**
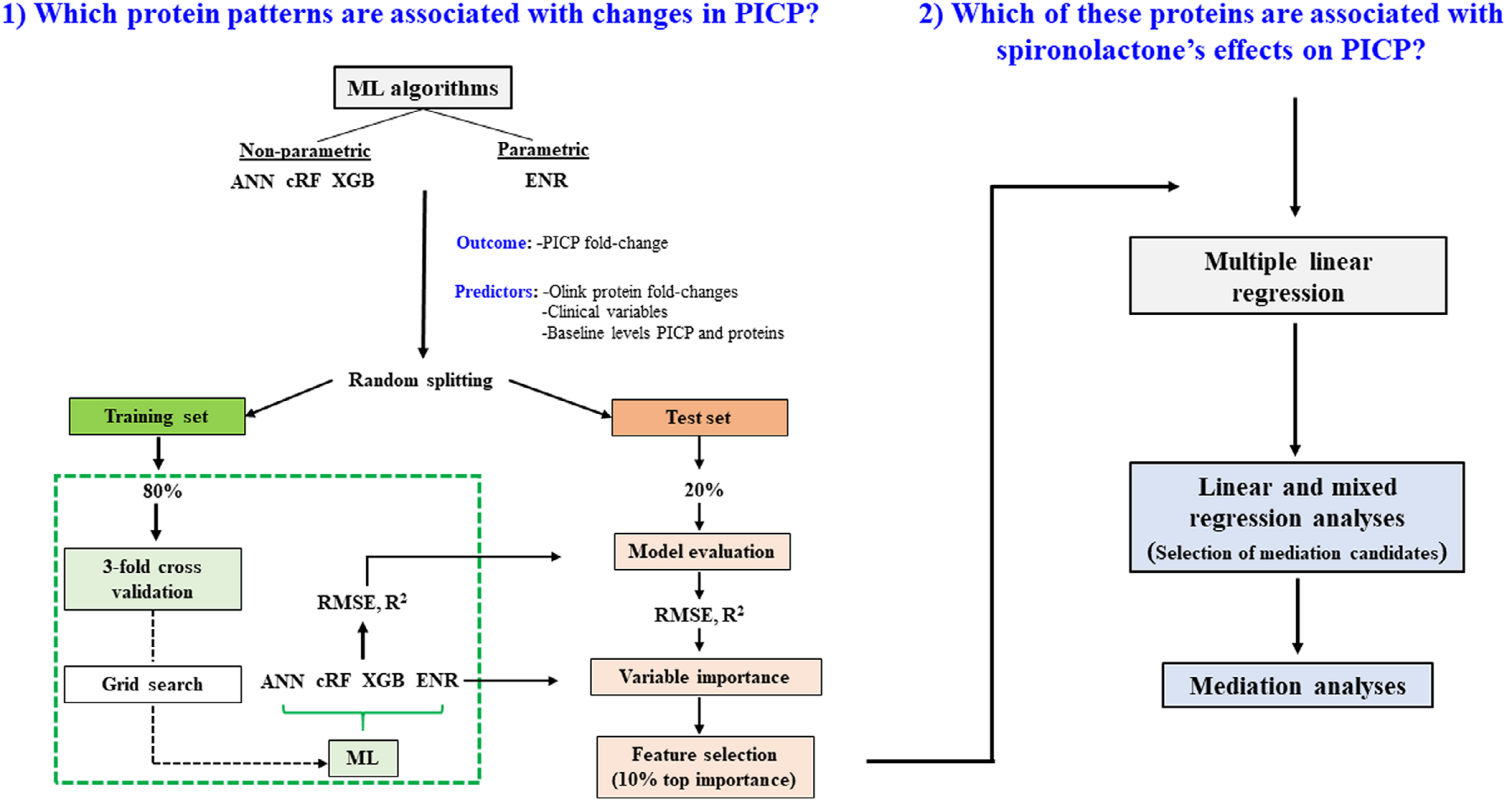
Flowchart illustrating the machine learning and statistical analyses workflow. PICP means procollagen Type I C‐terminal propeptide; ML, machine learning; RMSE, root mean square of residuals; ANN, artificial neural network; cRF, conditional random forest; XGB, extreme gradient boosting; ENR, elastic net regression.

To characterize the protein profiles associated with changes in serum PICP, we performed a series of complementary analyses including machine learning algorithms (MLAs), functional enrichment, association, and mediation analyses. An overview of the analytical workflow is provided in Figure 1, and a description of variables and participants is shown in Figure S1.

MLAs were performed to identify the most relevant clinical and proteomic predictors of changes in serum PICP. Model performance was evaluated in balanced training and test subsets, which showed no major differences in clinical characteristics (Table S3). The performance of all MLA models, assessed by RMSE and *R* ^2^ in both training and test sets, is shown in Figures S2 and S3.

Variable importance was analyzed to identify features most strongly associated with changes in serum PICP (importance graphs are shown in Figures S4–S7). As expected, baseline PICP and changes in collagen alpha‐1(I) chain (COL1A1) were the most influential predictors across all MLAs at both 1 (Figure S4) and 9 (Figure S5) months. To improve visualization of the remaining features, importance plots excluding these collagen‐related proteins are shown in Figures S6 and S7.

**FIGURE 2:**
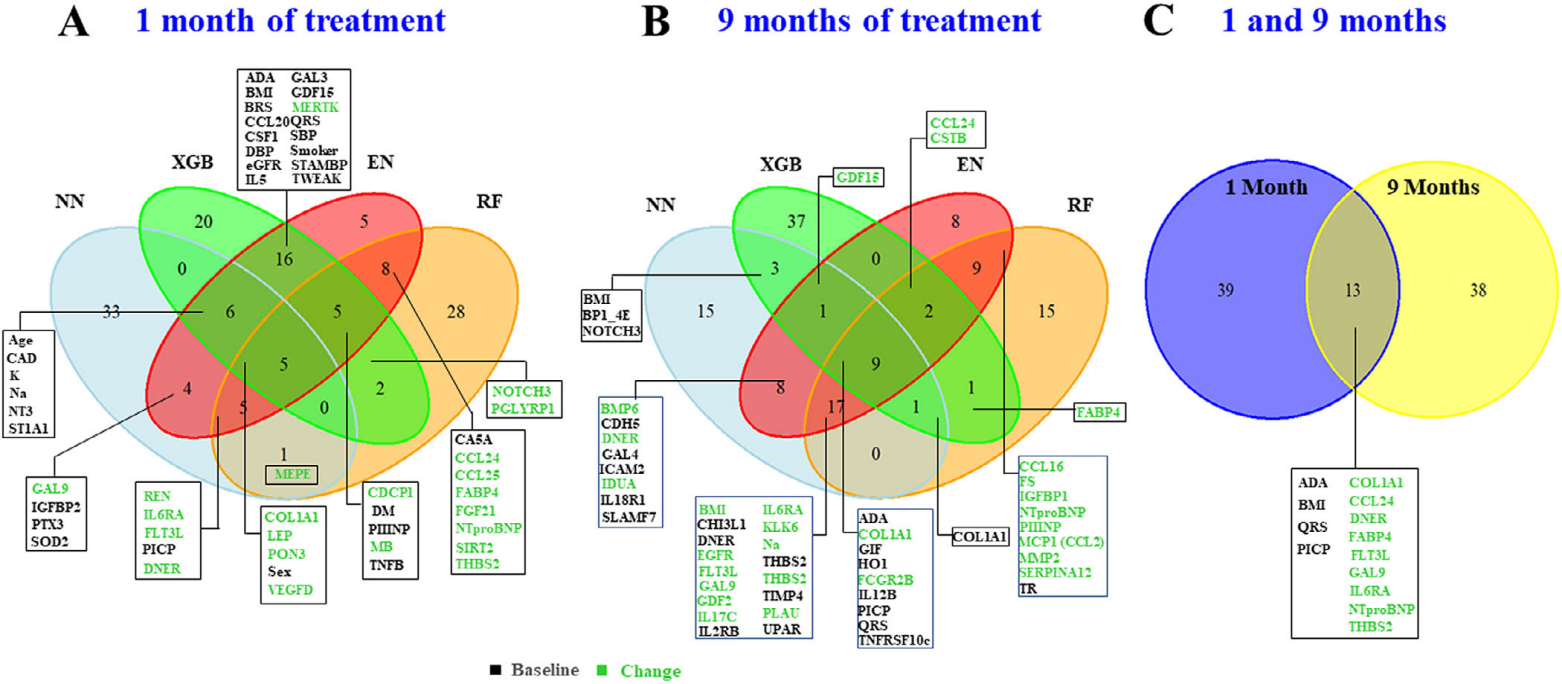
Selection of proteins associated with PICP changes after treatment. Venn diagrams highlighting baseline levels (black) and fold‐changes (green) of proteins selected if present within the 10% most important at least in two of the four machine learning models after 1 (A) and 9 (B) months of treatment, and the common proteins at both visits (C).

To identify parameters consistently associated with PICP changes across different modeling strategies, we compared the top 10% most influential variables selected by the four MLAs at 1 and 9 months. Venn diagrams illustrating the overlap among models are presented in Figure 2A,B. Variables identified by at least two models were considered consistently predictive of PICP change. Using this approach, COL1A1, NT‐proBNP, C–C motif chemokine ligand 24 (CCL24), notch‐like epidermal growth factor‐related receptor (DNER), fatty acid binding protein 4 (FABP4), FMS‐related receptor tyrosine kinase 3 ligand (FLT3L), galectin‐9 (GAL9), interleukin‐6 receptor subunit alpha (IL6RA), and THBS2 emerged as the most consistently influential variables explaining PICP variations at both time‐points (Figure 2C). Partial dependence plots illustrating the direction and magnitude of these associations are shown in Figures S8 and S9.

Of note, among the clinical variables included in the MLA models (age, sex, current smoker, diabetes mellitus [DM], coronary artery disease [CAD], QRS, as well as the baseline and fold‐change values of body mass index, systolic and diastolic blood pressure, serum sodium and potassium, eGFR, and breathlessness scale; Figure S1), only baseline body mass index and QRS were strongly associated with PICP reduction (Figure 2C).

### 2.3 HOMAGE: Functional Enrichment of MLA‐Selected Proteins

**FIGURE 3:**
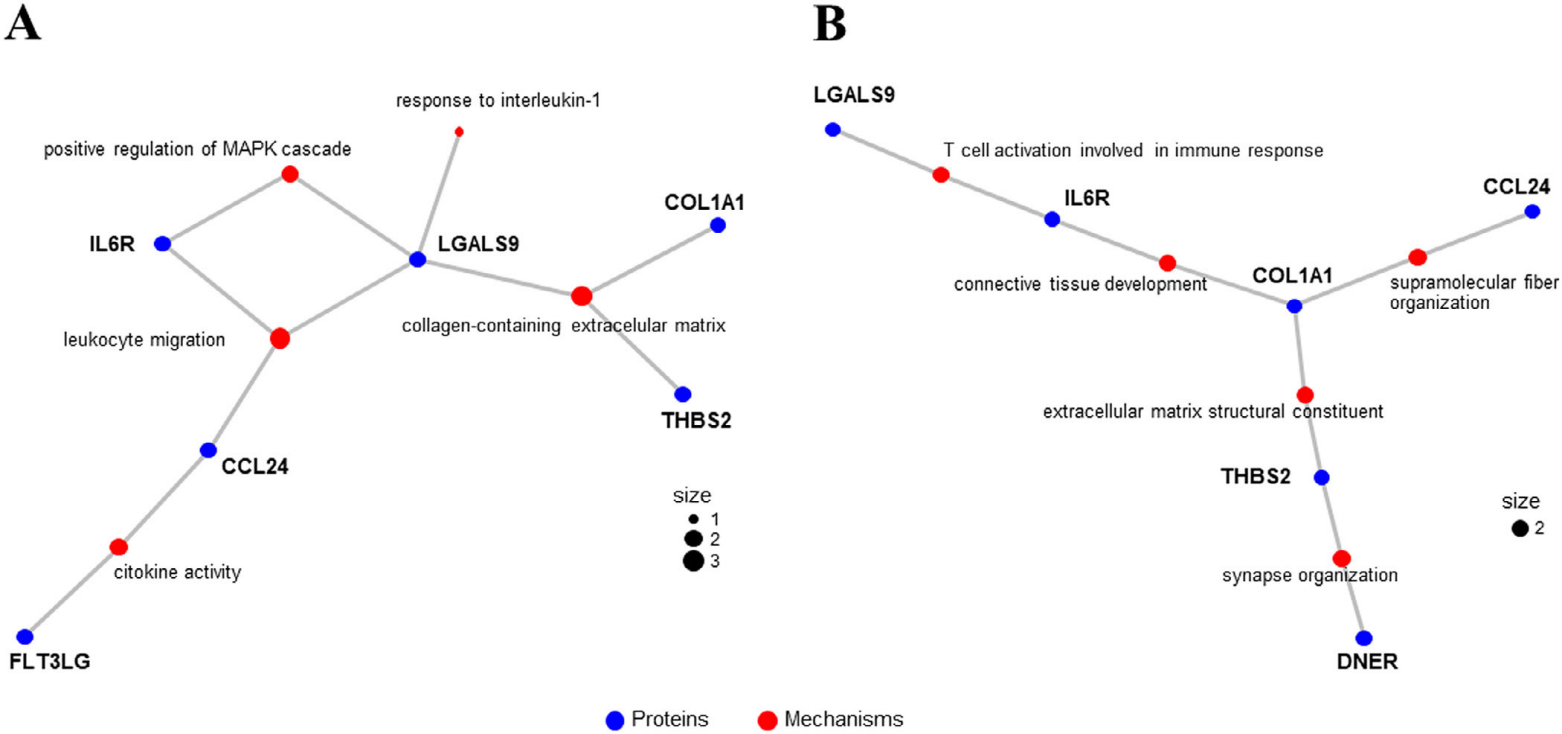
Functional enrichment analyses. Cnetplots showing the connections among the five most significantly enriched GO terms using the human genome as the background (A), and among the top enriched GO terms (fold enrichment > 5, unadjusted *p* < 0.05) using the list of all Olink proteins determined as the background (B), for the MLA‐selected proteins associated with PICP fold‐changes at 1 and 9 months. Proteins are shown in blue, GO terms in red, and dot size reflects the number of genes per term. GO, Gene Ontology; MLA, machine learning algorithms.

To gain biological insight into the MLA‐selected proteins, functional enrichment analyses were performed. GO analyses revealed enrichment in pathways related to immune response, inflammation, survival, and extracellular collagen matrix (Figure 3A). Sensitivity analyses using the targeted list of proteins as background further highlighted extracellular matrix‐related pathways among the most enriched categories (fold‐enrichment values >5, nonadjusted *p* values <0.05; Figure 3B). Correlations between MLA‐selected proteins and enriched Gene Ontology (GO) and Kyoto Encyclopedia of Genes and Genomes analysis (KEGG) pathways are shown in Figures S10 and S11.

### 2.4 HOMAGE: Mediation Analyses of Spironolactone Effect

Mediation analyses were performed to evaluate the extent to which spironolactone‐induced PICP reduction was mediated by proteins changing in parallel with PICP. COL1A1 was excluded from these analyses because of its direct structural relationship with PICP, which is part of collagen Type I, and could mask the mediation effects attributable to other proteins. Candidate mediators were selected based on whether spironolactone modified their association slope with PICP (Figure S12) and/or their expression relative to baseline (Table S4).

**FIGURE 4:**
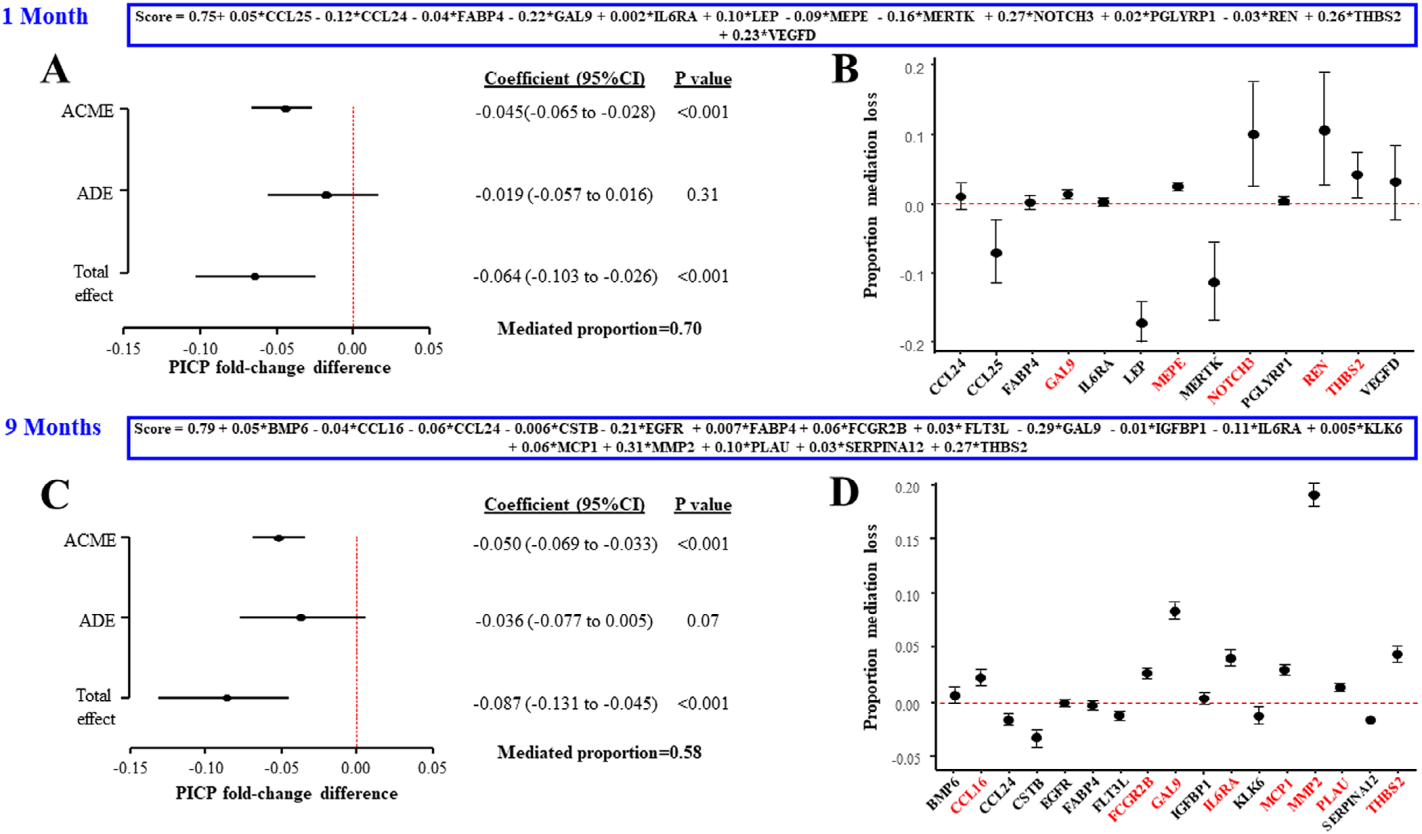
Mediation analyses in HOMAGE clinical trial. Effect decomposition plots for the combination of the selected candidates in mediator signature scores derived from linear regression models using PICP fold‐change as the continuous target and including all selected protein fold‐changes as independent variables at 1 (A) and 9 months (C). These scores rendered mediated proportions of 0.70 and 0.58, suggesting that the combined variation in these proteins mediated 70 and 58% of the total effect of spironolactone on PICP at 1 (A) and 9 (C) months, respectively. Graphs depicting mediation loss after removal of each protein from the mediator scores estimated at 1 and 9 months of spironolactone are shown in panels B and D, respectively. Estimates are accompanied by 95% percentile bootstrap confidence intervals based on 1000 bootstrap resamples. Variables in red are those with higher proportion mediation loss values, indicating greater importance in the mediation. ACME, average causal mediation effect; ADE, average direct effect.

*One month‐mediation analyses*: Candidate mediators included CCL24, FABP4, GAL9, IL6RA, THBS2, matrix extracellular phosphoglycoprotein (MEPE), peptidoglycan recognition protein 1, CCL25, leptin, notch receptor 3 (NOTCH3), MER proto‐oncogene tyrosine kinase, REN, and vascular endothelial growth factor D. These proteins were combined in a linear model to derive a score that rendered a mediated proportion of 0.70, suggesting that 70% of the overall spironolactone effect on PICP at 1 month was mediated by this group of proteins (Figure 4A). To assess the relative contribution of each individual mediator within this multivariable framework, we calculated the “mediated proportion loss” by sequentially removing each protein from the full model and recalculating the mediated proportion. Using this approach, REN and NOTCH3 emerged as the most influential mediators, followed by THBS2, MEPE, and GAL9 (Figure 4B).

*Nine months‐mediation analyses*: Candidate mediators included CCL24, FABP4, GAL9, IL6RA, THBS2, bone morphogenetic protein 6, CCL16, CSTB, epidermal growth factor receptor (EGFR), FCGR2B, FLT3L, insulin‐like growth factor binding protein 1, kallikrein‐related peptidase 6, C–C motif chemokine ligand 2 (MCP1), matrix metallopeptidase 2 (MMP2), serpin family A member 12, and plasminogen activator, urokinase (PLAU). These proteins were combined in a linear model to derive a score that rendered a mediated proportion of 0.58 (Figure 4C), suggesting that 58% of the overall spironolactone effect on PICP at 9 months was mediated by this group of proteins. As in the 1‐month analysis, the relative contribution of each mediator was assessed by estimating the mediated proportion loss after sequential exclusion of individual proteins. Using this approach, the most important mediator at 9 months was MMP2, followed by GAL9, THBS2, IL6‐RA, MCP1, FCGR2B, CCL16, and PLAU (Figure 4D).

Overall, mediation analyses suggest that GAL9 and THBS2 may be consistent mediators of spironolactone‐induced PICP reduction at both 1 and 9 months, independently of hemodynamic, electrolyte, and renal effects (Figure S13). GO enrichment analyses confirmed their involvement with collagen‐containing extracellular matrix mechanisms (Figure 3).

### 2.5 HOMAGE: Combined THBS2 and GAL9 Association With PICP and Cardiac Trajectories

**FIGURE 5:**
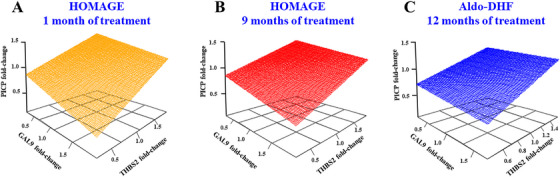
Association of changes in plasma THBS2 and GAL9 with the decrease in serum PICP. 3D scatterplots with regression planes depicting THBS2 and GAL9 fold‐changes associated with PICP fold‐changes in patients from the HOMAGE trial after 1 month (A) and 9 months (B) of spironolactone treatment, and in patients from the Aldo‐DHF trial after 12 months of treatment (C). Multiple linear regressions were adjusted by the baseline values of PICP, THBS2, and GAL9.

**FIGURE 6:**
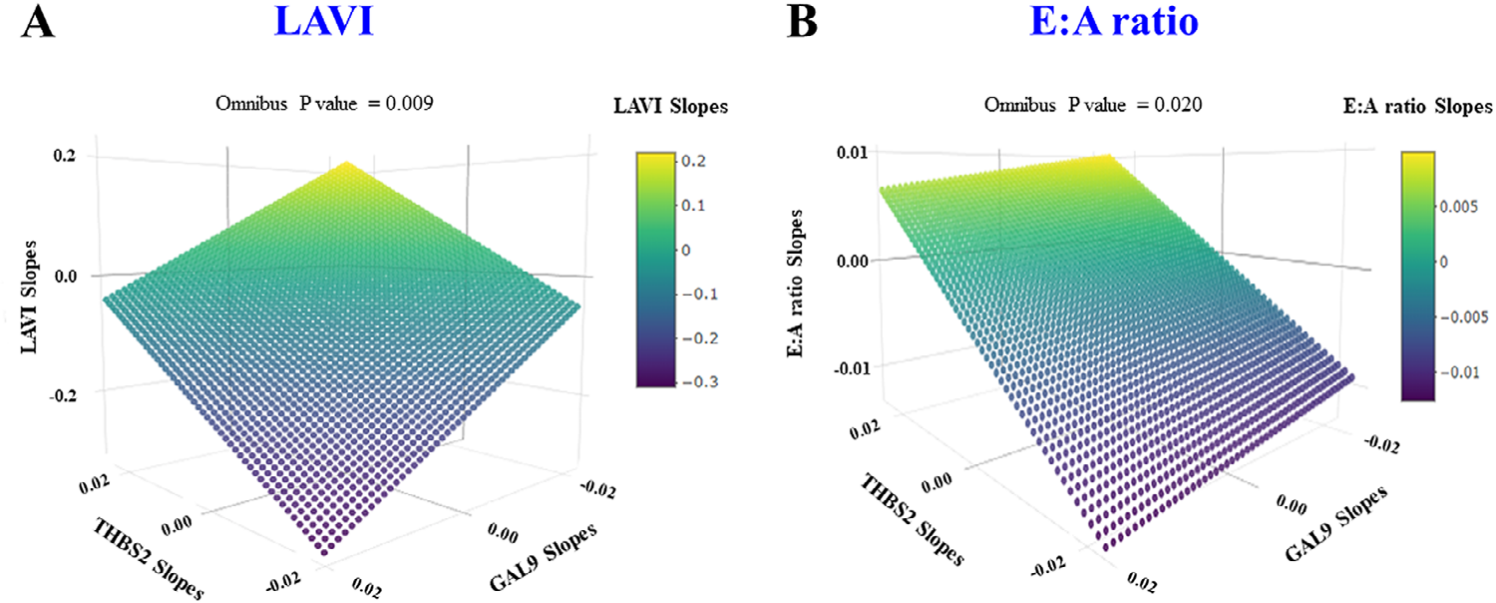
Associations between plasma THBS2 and GAL9 slopes and the slopes of LAVI and *E*:*A* ratio in HOMAGE. 3D scatterplots with regression planes depict the predicted subject‐specific slopes of LAVI (A) and the *E*:*A* ratio (B) as a function of subject‐specific slopes of THBS2 and GAL9. Subject‐specific slopes were estimated as random slopes from linear mixed‐effects models using all three visits (basal, 1, and 9 months). Multivariable linear models assessing these associations were adjusted for age, sex, study arm, and eGFR. The color scale indicates the magnitude of the predicted slopes. Omnibus *p* values refer to the joint statistical significance of THBS2 and GAL9 slopes in the multivariable models.

Given their consistent mediating roles, the joint association of THBS2 and GAL9 with PICP was further explored. Three‐dimensional (3D) regression models showed that reductions in THBS2 together with increases in GAL9 were associated with decreases in PICP at both 1 and 9 months (Figure 5A,B). Longitudinal trajectory analyses across all time points further showed that this joint GAL9–THBS2 pattern was also associated with steeper reductions in LAVI (Figure 6A) and *E*:*A* ratio (Figure 6B). No associations were observed for other echocardiographic parameters (Table S5).

### 2.6 Aldo‐DHF: THBS2 and GAL9 Associations and Mediation at 12 Months

We next examined the associations of GAL9 and THBS2 with changes in serum PICP and their potential mediating role in the effects of spironolactone in HFpEF patients (Aldo‐DHF trial). Proteomic measurements were available in 368 HFpEF patients of whom 190 (51.6%) were treated with spironolactone for 12 months. Greater reductions in serum PICP were associated with spironolactone treatment, higher baseline serum sodium and PICP, and higher baseline plasma THBS2 (Table 2).

**TABLE 2:** Baseline characteristics by quartiles of the PICP fold‐change at 12 months (ALDO‐DHF trial).

	PICP quartiles				*p* Trend
	Q1 (*n* = 92)	Q2 (*n* = 92)	Q3 (*n* = 92)	Q4 (*n* = 92)	
PICP change (final/baseline)	<0.79	0.79–0.95	0.95–1.11	>1.11	
Demographics					
Age, years	66 (61–72)	68 (64–72)	68 (61–72)	69 (62–73)	0.364
Men, n (%)	42 (45.7)	43 (46.7)	46 (50.0)	44 (47.8)	0.674
Current smoker, n(%)	38 (41.3)	35 (38.0)	50 (54.3)	46 (50.0)	0.071
Prior medical history, *n* (%)					
Hypertension	85 (92.4)	84 (91.3)	85 (92.4)	82 (89.1)	0.455
Diabetes mellitus	13 (14.1)	16 (17.4)	14 (15.2)	18 (19.6)	0.421
CAD	32 (34.8)	36 (39.1)	44 (47.8)	33 (35.9)	0.613
MI	17 (18.5)	11 (12.0)	16 (17.4)	14 (15.2)	0.767
PCI	23 (25.0)	22 (23.9)	34 (37.0)	22 (23.9)	0.649
CABG	2 (2.2)	8 (8.7)	13 (14.1)	7 (7.6)	0.090
STIA	8 (8.7)	9 (9.8)	10 (10.9)	9 (9.8)	0.760
COPD	3 (3.3)	5 (5.4)	4 (4.3)	1 (1.1)	0.375
Randomized treatment, *n* (%)					
Spironolactone	57 (62.0)	48 (52.2)	42 (45.7)	43 (46.7)	0.025
Other baseline medications, *n* (%)					
ACE inhibitor	40 (43.5)	40 (43.5)	49 (53.3)	38 (41.3)	0.905
ARB	34 (37.0)	33 (35.9)	26 (28.3)	32 (34.8)	0.512
Beta‐blockers	64 (69.6)	64 (69.6)	70 (76.1)	67 (72.8)	0.457
Thiazide diuretics	39 (42.4)	38 (41.3)	33 (35.9)	44 (47.8)	0.651
Loop diuretics	16 (17.4)	12 (13.0)	19 (20.7)	12 (13.0)	0.743
Calcium channel blockers	17 (18.5)	26 (28.3)	24 (26.1)	27 (29.3)	0.138
Lipid‐lowering therapy	46 (50.0)	48 (52.2)	53 (57.6)	52 (56.5)	0.293
Aspirin	44 (47.8)	45 (48.9)	58 (63.0)	45 (48.9)	0.470
Any antiplatelet therapy	10 (10.9)	7 (7.6)	10 (10.9)	4 (4.3)	0.206
Physical examination					
BMI, kg/m^2^	29.4 (27.5–31.6)	28.7 (26.2–31.4)	29.4 (26.6–31.9)	29.2 (26.6–31.3)	0.630
HR, beats/min	66.0 (59.8–72.2)	65.0 (59.0–73.5)	65.5 (58.8–73.0)	64.0 (57.0–72.5)	0.426
SBP, mmHg	136 (126–148)	133 (123–146)	135 (124–147)	132 (124–145)	0.229
DBP, mmHg	79 (73–86)	77 (68–84)	81 (74–90)	80 (71–88)	0.526
NYHA class (IIIvsII)	15 (16.3)	18 (19.6)	8 (8.7)	13 (14.1)	0.288
Blood tests at baseline					
Hemoglobin, g/dL	13.8 (13.1–14.5)	13.7 (12.9–14.4)	13.7 (13.0–14.6)	13.8 (12.9–14.7)	0.952
Sodium, mmol/L	141 (140–143)	141 (139–143)	140 (139–142)	140 (138–142)	0.001
Potassium, mmol/L	4.2 (4.0–4.5)	4.2 (3.9–4.5)	4.2 (3.9–4.4)	4.2 (3.9–4.4)	0.341
eGFR, mL/min/1.73 m^2^	81 (67–90)	74 (61–87)	80 (67–90)	73 (61–88)	0.190
Echocardiography					
LVEDV, mL	69.0 (54.0–98.0)	68.0 (50.0–91.0)	76.0 (59.0–98.5)	76.0 (59.0–97.0)	0.367
LVEF, %	67.0 (60.0–73.2)	68.0 (63.0–74.0)	67.0 (62.0–73.0)	66.0 (60.0–71.0)	0.247
LVMI, g/m^2^	105 (89.7–118)	104 (86.0–120)	110 (94.5–126)	108 (97.1–132)	0.077
LAVI, mL/m^2^	26.2 (22.0–34.0)	26.4 (21.9–30.9)	26.6 (22.3–32.9)	26.8 (23.6–33.5)	0.488
*E*:*A* ratio	0.8 (0.7–0.9)	0.8 (0.7–0.9)	0.8 (0.7–0.9)	0.8 (0.7–0.9)	0.830
*E*:*e*′ ratio	11.9 (10.6–15.3)	11.8 (10.1–14.1)	11.7 (9.9–13.7)	12.0 (10.5–13.8)	0.545
Blood biomarkers at baseline					
NT‐proBNP, ng/L	191 (83.2–378)	127 (66.1–282)	185 (92.2–311)	163 (93.2–292)	0.909
PICP, µg/L	133 (112–155)	112 (89.8–138)	110 (88.4–130)	98.7 (79.3–112)	**<0.001**

Abbreviations as in Table 1.

Consistent with the HOMAGE findings, a decline in serum PICP was associated with increases in GAL9 and decreases in THBS2 over 12 months (Figure 5C). Mediation analyses suggested that these two proteins accounted for approximately 12% of the spironolactone‐induced PICP reduction in Aldo‐DHF (Figure S14A–D).

While baseline GAL9 was not associated with changes in PICP, higher baseline THBS2 was consistently associated with greater PICP reduction in both studies (Tables S2 and S6). Since spironolactone may impact PICP through the regulation of THBS2, we further investigated the role of this protein. Interaction analyses showed that higher baseline THBS2 was associated with PICP reduction only in patients treated with spironolactone (*p* for interaction = 0.002; Figure S15A). Stratification by the THBS2 median (NPX = 5.95) showed that spironolactone reduced PICP only in patients with high baseline THBS2 (Figure S15B,C), with a similar trend observed in HOMAGE (Figure S16).

## 3 Discussion

To the best of our knowledge, this is the first study investigating the interaction between plasma proteomic changes and changes in serum PICP, a marker of collagen metabolism and fibrosis, in response to spironolactone in patients with or at risk of HFpEF. Previous studies have shown a direct association between PICP and cardiac fibrosis in different cardiac conditions, supporting its potential value as a biomarker of cardiac collagen synthesis [1]. However, it should be noted that PICP is not a cardiac‐specific biomarker, as it can also be derived from other organs. Its elevation may reflect systemic profibrotic activation in HFpEF, involving not only the heart but also other organs affected by comorbidities such as hypertension and chronic kidney disease. In this study, a reduction in PICP was associated with changes in many CV‐ and inflammation‐related proteins. Among them, the effect of spironolactone on PICP was associated with greater decreases in THBS2 and increases in GAL9 during follow‐up.

The proteomic associations with changes in PICP found in the MLA analysis should be considered as an interconnected network rather than individual associations. The association between a specific protein and PICP should be interpreted with caution. Nevertheless, the strong association between PICP and COL1A1 reinforces the value of PICP as a biomarker of collagen Type I metabolism. The stronger spironolactone‐induced reduction of PICP in patients with higher PICP at baseline has also been reported previously [7]. Of interest, in HOMAGE, changes in PICP were consistently associated with changes in NT‐proBNP both at 1 and 9 months of treatment with spironolactone, reinforcing the notion that reduced collagen synthesis and fibrosis may contribute to improved myocardial remodeling and decreased cardiac stress. This direct association between PICP and NT‐proBNP has been previously reported in other studies [8]. On the other hand, no consistent association was observed between PICP and hs‐TnT, which might be expected in this population at risk but without overt HF and low myocardial damage.

Overall, most of the molecules found to be related to PICP in the MLA analyses were classified as inflammation and fibrosis‐related proteins. For instance, associations between CCL24 [9] and IL6RA [10] with inflammation and fibrosis pathways in experimental models of cardiac damage have been reported. In addition, FABP4 is associated with inflammatory markers in patients with HF and atrial fibrillation [11], and FLT3 is related to inflammation and fibrosis in experimental cardiac remodeling [12, 13].

Spironolactone may drive some of the observed associations that, even if weak, may be relevant in a multidimensional context, because simultaneous small changes in multiple proteins may have synergistic effects. Mediation analyses suggested that around 60–70% of spironolactone's effects on PICP may be mediated by the MLA‐selected proteins combined, although only THBS2 and GAL9 emerged as potential mediators at both 1 and 9 months. Other significant mediators of spironolactone‐induced PICP reduction at 9 months were MMP2 and MCP1. This is in accordance with previous data showing that MRAs reduce MMP2 expression in parallel with cardiac fibrosis in experimental autoimmune myocarditis [14] and diminish its vascular expression in stroke‐prone spontaneously hypertensive rats [15]. MCP1, involved in macrophage recruitment, plays also a role in MR‐mediated cardiac fibrosis [16].

However, mediation analyses should be interpreted as hypothesis generating, as causal effects cannot be inferred from their results. Mechanistic validation in experimental models would be necessary to establish the biological relevance of the identified candidates and to disentangle the proteomic signatures reflecting the direct effects of spironolactone on cardiac fibrosis from those related to its influence on other pathophysiological processes (e.g., blood pressure reduction) or compensatory responses (e.g., increased REN levels) [17]. After 1 month, spironolactone reduced weight, serum sodium concentration and blood pressure, suggesting diuresis and natriuresis, which may also explain the association between the increase in REN and the decline in PICP. However, changes in blood pressure, sodium, potassium, or eGFR were not among the most significant variables associated with PICP changes, suggesting a modest influence of these parameters in the change in PICP. Further research is needed to distinguish the percentage corresponding to an active mediation from a passive reflection of the systemic responses triggered by spironolactone. The response to initiating an MRA will vary over time, as the body finds a new steady state. The effects of an MRA may be most obvious before the new steady state is established. Indeed, mediation analysis suggested stronger associations at 1 month than at 9 months.

The contribution of galectins to CV disease is well established, but little is known on the specific role of GAL9 in HF [18]. This protein has been described as an anti‐inflammatory and antifibrotic factor mainly in pulmonary disease [19, 20], acting through mechanisms that involve TGF‐β signaling [21]. Administration of GAL9 to CVB3‐infected mice reduced myocarditis severity and mortality [22]. In accordance with its antifibrotic potential, we found that an increase in GAL9 was associated with a decrease in PICP in spironolactone‐treated patients. Of interest, GAL9 may induce cardiac myofibroblast apoptosis [23] and ameliorate myocardial fibrosis in experimental models of cardiac pressure overload [24]. To the best of our knowledge the connection between GAL9 and aldosterone has not been previously explored.

THBS2 appears to be a key modulator of fibrogenesis, specifically affecting collagen production. We found that THBS2 decreases in parallel with PICP reduction. While mechanistic studies will be needed to establish a direct link between aldosterone and THBS2 regulation, some available evidence supports this hypothesis. Aldosterone increases cardiac expression of TGF‐β [25], while TGF‐β has been shown to regulate THBS2 expression in cancer associated fibroblasts [26]. We also found that spironolactone reduced PICP especially in patients with HFpEF and high baseline THBS2, suggesting that THBS2 could be a potential target for antifibrotic therapies. In this context, some data suggest that SGLT2i may reduce serum THBS2 [27], while exerting antifibrotic effects. The effects of other existing or novel CV therapies on these biomarkers should be explored [28, 29].

We have not evaluated the prognostic value of THBS2 in our study, but existing evidence supports its association with progression of CV disease and adverse outcomes [30]. In particular, increased THBS2 circulating levels have been associated with poor CV outcomes (including HF hospitalizations and mortality) in patients both with HFpEF [31] and HF with reduced ejection fraction [32] and with HF hospitalization in diabetic patients [33].

THBS2 and GAL9 are not cardiac specific and are involved in the development of fibrosis in other organs, which may also be affected in multimorbid patients at risk of HFpEF. In this context, MRAs have been shown to reduce fibrosis in the aorta [34] and kidneys [35, 36]. Therefore, the antifibrotic effects of spironolactone, and its impact on THBS2 and GAL9, may not be limited to the heart but could also play a role in other tissues affected by fibrotic remodeling.

Of interest, we found that decreasing THBS2 combined with increasing GAL9 was associated with an improvement in the LAVI and the *E*:*A* ratio, parameters of diastolic dysfunction which were modified by spironolactone in the HOMAGE clinical trial [3]. This finding suggests a coordinated interplay between these circulating proteins, collagen Type I turnover, and the progression of diastolic dysfunction. Overall, this analysis is the first to hypothesize that simultaneous modulation of both THBS2 and GAL9 may be involved in spironolactone‐mediated inhibition of fibrosis. Nonetheless, additional mechanistic work in in vitro assays (e.g., fibroblast cultures exposed to spironolactone) and in vivo animal models will be needed to determine whether GAL9 and THBS2 play causal roles in mediating the antifibrotic effects of spironolactone and whether these are independent of hemodynamic or renal effects.

### 3.1 Study Limitations

We tested the effect of spironolactone on multiple proteins by applying four different MLAs, permitting accommodation of nonlinear as well as linear analyses of dynamic changes, and selecting only proteins more strongly linked to changes in PICP. Including nontargeted proteomic approaches, or a larger array of proteins, could have provided further insights into the inhibitory actions of spironolactone on collagen synthesis. For the Aldo‐DHF trial, only one of the three Olink panels was available; a more complete analysis would be preferable. The observed reductions in PICP with spironolactone were modest in absolute terms, and their specific biological meaning remains to be fully established. Serum PICP reflects systemic collagen metabolism rather than being cardiac specific, although we have previously shown a strong association with histologically assessed myocardial fibrosis [1]. Furthermore, circulating levels can be affected by pathologies affecting hepatic or bone metabolism. The myocardium may be susceptible to both systemic and local drivers of fibrosis and myocardial function may be more sensitive to the effects of fibrosis than other organs. Mediation analyses are hypothesis‐generating tools and no causal effects can be concluded from their results. These observations need validation in further cohorts of patients at risk or with HF with preserved ejection fraction.

### 3.2 Conclusions

In a plasma proteomic analysis of patients with or at risk of HFpEF, we have identified molecular pathways associated with spironolactone‐induced reductions in PICP, a marker of collagen Type I synthesis. A decline in THBS2 and an increase in GAL9 might mediate the effects of spironolactone on PICP and therefore its antifibrotic effects. Further mechanistic studies are warranted to validate this hypothesis and elucidate the potential antifibrotic of modulating these candidates, as well as the utility of the combined assessment of THBS2 and GAL9 to track changes in fibrosis.

## 4 Materials and Methods

### 4.1 Study Populations

This is a posthoc, exploratory, observational analysis based on data from two randomized controlled trials: the HOMAGE trial (ClinicalTrials.gov identifier NCT02556450) and the Aldo‐DHF trial (NCT00624431). For both studies, the respective protocols were approved by all relevant Ethics Committees and regulatory bodies [3, 37]. All participants provided written informed consent prior to study‐specific procedures.

HOMAGE was a multicenter trial including people at increased risk of developing heart failure who were randomly assigned, open‐label, to receive spironolactone or not, in addition to background therapy, excluding MRAs or loop diuretics [3]. Participants were followed for up to 9 months.

Aldo‐DHF was a multicenter trial including patients with HFpEF who were randomly assigned, double‐blind, to spironolactone or placebo [37]. Sixteen percent of the patients were treated with loop diuretics. Patients were followed for 12 months.

For more details on inclusion and exclusion criteria in both trials, see the Supporting Information.

### 4.2 Biomarkers

Serum PICP was measured using an enzyme‐linked immune assay (Quidel Corporation), and NT‐proBNP and sensitivity troponin T (hs‐TnT) were measured by electro‐chemiluminescent assays (Roche diagnostics) as previously described [3, 4, 37].

### 4.3 Targeted Proteomics

For HOMAGE participants, baseline, 1‐month, and 9‐month (or “last visit”) plasma samples were analyzed for 276 proteins by the TATAA‐biocenter using the Olink Proseek Multiplex CV II, CV III, and inflammation panels (Table S1) [38]. For Aldo‐DHF patients, the CV II Olink panel was measured in plasma samples [39]. The data generated are given in the form of log_2_‐transformed relative quantification (NPX values).

### 4.4 Machine Learning Analyses

Patients from the HOMAGE trial were randomly assigned to training (80%) and test (20%) sets, and the MLAs artificial neural network (R packages *Keras* and *Tensorflow* as interfaces to these packages in Python environment), conditional random forest (R package *party*), extreme gradient boosting (R package *xgboost*), and linear elastic‐net penalized regression (R package *glmnet*) were performed. PICP fold‐change (final/baseline values) was the continuous dependent variable, and the Olink protein baseline and fold‐change (2^final/2^baseline NPX) values, baseline PICP and the clinical variables age, sex, current smoker, DM, CAD and QRS, and the baseline and fold‐change values of body mass index, systolic and diastolic blood pressure, serum sodium and potassium, eGFR and breathlessness scale, were considered as predictors (Figure S1). Optimal normalization was achieved by rescaling the data within the range 0–1. These associations were explored both at 1‐ and 9‐month visits. For MLA model development, we applied threefold cross‐validation and grid search to achieve hyperparameter optimization and to prevent overfitting. The optimal hyperparameter set for each algorithm is shown in the Table S7. Variable importance was computed following a 1000 permutation‐based approach and represented in bar charts (the longer the bar chart, the higher the importance of the feature in each ML) using the *DALEX* R package.

### 4.5 Function Enrichment Analyses

Functional enrichment analysis was performed using *clusterProfiler* R package and the GO and KEGG databases. The human genome and the list of all proteins examined were used as background references. The terms were considered significant if their associated *p* and *q* values were <0.05 (Benjamin–Hochberg method).

For more details on MLA and functional enrichment analyses, see the Supporting Information.

### 4.6 Statistical Analyses

Continuous data are expressed as median with 25th and 75th percentiles, and categorical variables as numbers and percentages. Spearman and Cochran–Armitage tests were used to estimate *p* for trend values for continuous and categorical variables, respectively, across quartiles of the PICP fold‐change.

Interaction analyses were performed to test the influence of spironolactone on the slopes of the PICP fold‐change (as a continuous variable) regressed on the MLA‐selected variables. The linear regression models included the randomization variable, the MLA‐selected protein fold‐change, their interaction term, eGFR fold‐change, and baseline PICP and ML‐selected proteins. The effect of spironolactone on the circulating levels of the MLA‐selected proteins at 1 and 9 months (dependent variables) was assessed using mixed‐effect models with the randomization variable, the respective baseline protein levels, and the eGFR fold‐change values as fixed‐effects variables. The heteroscedasticity and normality of the residuals were examined by graphic analysis of the scatterplots and *Q*–*Q* plots, respectively.

Mediation analyses were performed (R package *mediation*) to investigate whether the effect of spironolactone (independent variable) on the PICP fold‐change (continuous dependent variable) was indirectly explained by those ML‐selected proteins that were themselves influenced by spironolactone (mediation candidates). All candidate protein fold‐changes were included in a linear regression model and a mediation signature score was generated. Then, the following regression analyses were run: (1) the mediator model for the conditional distribution of the mediation score given the treatment (spironolactone) and the observed pretreatment covariates (baseline values of the mediators and PICP); and (2) the outcome model for the conditional distribution of the outcome (PICP fold‐change) given the treatment, the mediation score, and the pretreatment covariates. These models were fitted separately and comprised as the main inputs to the *mediate* function, which computes the estimated average causal mediation effects (ACMEs), average direct effects (ADEs), total effects (TEs), and the mediated proportion (ACME/TE). To estimate the importance of each variable in the mediation effect, a new mediation score was calculated after removing the mediator of interest, the mediation analyses were then rerun, and the new mediation proportion was subtracted from the one comprising all the mediators. All estimates were accompanied by 95% percentile bootstrap confidence intervals (CI) based on 1000 bootstrap resamples.

Longitudinal biomarker trajectories were modeled using linear mixed‐effects models with random intercepts and random slopes for time to obtain subject‐specific linear trends across basal, 1‐, and 9‐month visits. For each variable, we extracted the subject‐specific random slope using the *ranef* function. Subject‐specific slopes of the variables of interest were then regressed on subject‐specific slopes of GAL9 and THBS2 using ordinary least squares linear regression, adjusting for the previously mentioned confounding variables. Predicted surfaces of LAVI and *E*:*A* ratio slopes versus GAL9 and THBS2 slopes (covariates held at their sample means) were visualized using a 3D prediction grid.

Model adequacy was assessed by inspection of residuals, evaluation of linearity and heteroscedasticity, and influence diagnostics. As a robustness check, we re‐estimated the regression models using a robust regression estimator (*lmrob*) when necessary.

Statistical significance was set as a two‐sided *p* of 0.05. The statistical analyses were performed by using R (version 4.5.1) software.

A flow chart illustrating the sequence of steps for MLA and conventional statistical analysis is given in the Figure 1. For more details, see the Supporting Information.

## Author Contributions

A.G. and S.R. conceived, designed, and drafted the manuscript. S.R. performed the statistical and ML analyses. J.G.F.C. and N.G. participated in designing the study and helped to draft the manuscript. F.E., J.P.F., D.Z., P.P., F.C., J.P., S.H., H.P.B.L.R., B.P., C.D., A.L.C., J.D., and F.Z. collected, evaluated the clinical data, and revised the manuscript. B.L., G.S.J., and I.L. collected the biomarker data and revised the manuscript. All authors have read and approved the final manuscript.

## Funding

This project was funded by the Horizon Europe ERA4HEALTH CARDINNOV program (Project PCI2024‐153490 funded by MICIU/AEI /10.13039/501100011033 and Co‐funded by the European Union) and European Union Commission's Seventh Framework Programme under grant agreement 305507 (HOMAGE [Heart Omics in Ageing consortium]) awarded to Inserm, CIMA Universidad de Navarra, University of Glasgow, Charité — Universitätsmedizin Berlin, Maastricht University, Hull University; the Instituto de Salud Carlos III (CIBERCV CB16/11/00483, PI20/01319, and PI21/00946 co‐financed with ERDF funds, awarded to the Laboratory of Heart Failure, CIMA Universidad de Navarra), the Agencia Estatal de Investigación and the La Marató de TV3 Foundation (202321‐31) awarded to the Laboratory of Heart Failure, CIMA Universidad de Navarra.

## Conflicts of Interest

J.G.F.C. reports research grants paid to the institution from Bristol‐Myers‐Squibb, CSL‐Vifor, and Pharmacosmos, honoraria paid to the institution from Medtronic and honoraria for speaking from Edwards Ltd and Pharmacosmos. N.G. reports honoraria from Bayer, Boehringer, Echosens, Lilly, Novonordisk, Novartis, and NP Medical. F.Z. reports personal fees from 89bio, Applied Therapeutics, Bayer, Betagenon, Biopeutics, Boehringer, CVRx, Cardior, Cereno Pharmaceutical, Cellprothera, Merck, Northsea, Otsuka, and Owkin. H.P.B.R. reports grants from Roche Diagnostics, consulting fees from Novartis, Boehringer Ingelheim, Vifor Pharma, Roche Diagnostics, and AstraZeneca, payment for export testimony from Novartis, participation in board of CeleCor Therapeutics, and received equipment and materials from Roche Diagnostics. Arantxa González is an Editorial board member of MedComm. Arantxa González was not involved in the journal's review of or decisions related to this manuscript. The other authors declare no conflicts of interest.

## Ethics Statement

The HOMAGE^3^ trial was conducted in nine centers in the UK, France, Italy, Ireland, Germany, and the Netherlands. The trial was approved by relevant ethics committees and regulatory bodies: Greater Manchester Central Research Ethics Committee (no. 16/NW/0012; EudraCT number: 2015‐000413‐48); Comité de Protection des Personnes Est‐III, Hôpital de Brabois (no. 15.03.04); Comitato Etico Regione Toscana (no. 378/CEAVSE); Ethics and Medical Research Committee (no. 16/6/2015); Ethik‐Kommission des Landes Berlin (no. 7.0.21/07/2016); De medisch‐ethische toetsingscommissie (no. NL52729.068.15).

The ALDO‐DHF [37] trial was approved by the Ethics Committee of the University of Göttingen Medical Center (#6/12/06) and all participating sites, that is, the Ethics Committees at the Universities of Berlin (#566/06), Cologne (#06–229), and Würzburg (#179/06), and the Medical Associations of Hamburg (#M‐311–06) and Munich (#411–06). Trial registration: ISRCTN94726526; Eudra‐CT No: 2006‐002605‐31.

All participants provided written informed consent prior to study‐specific procedures.

## Supporting information




**Supporting File 1**: mco270634‐sup‐0001‐SuppMat.docx

## Data Availability

The data underlying this article will be shared on reasonable request to the corresponding authors.

## References

[1] S. Ravassa , B. López , T. A. Treibel , et al., “Cardiac Fibrosis in Heart Failure: Focus on Non‐invasive Diagnosis and Emerging Therapeutic Strategies,” Molecular Aspects of Medicine 93 (2023): 101194. 37384998 10.1016/j.mam.2023.101194

[2] R. Agarwal , P. Kolkhof , G. Bakris , et al., “Steroidal and Non‐steroidal Mineralocorticoid Receptor Antagonists in Cardiorenal Medicine,” European Heart Journal 42 (2021): 152–161. 33099609 10.1093/eurheartj/ehaa736 PMC7813624

[3] J. G. F. Cleland , J. P. Ferreira , B. Mariottoni , et al., “The Effect of Spironolactone on Cardiovascular Function and Markers of Fibrosis in People at Increased Risk of Developing Heart Failure: The Heart ‘OMics’ in AGEing (HOMAGE) Randomized Clinical Trial,” European Heart Journal 42 (2021): 684–696. 33215209 10.1093/eurheartj/ehaa758 PMC7878013

[4] S. Ravassa , T. Trippel , D. Bach , et al., “Biomarker‐based Phenotyping of Myocardial Fibrosis Identifies Patients With Heart Failure With Preserved Ejection Fraction Resistant to the Beneficial Effects of Spironolactone: Results From the Aldo‐DHF Trial,” European Journal of Heart Failure 20 (2018): 1290–1299. 29709099 10.1002/ejhf.1194

[5] A. Bayes‐Genis , P. P. Liu , D. E. Lanfear , et al., “Omics Phenotyping in Heart Failure: The next Frontier,” European Heart Journal 41 (2020): 3477–3484. 32337540 10.1093/eurheartj/ehaa270

[6] A. González , A. M. Richards , R. A. de Boer , et al., “Cardiac Remodelling—part 1: From Cells and Tissues to Circulating Biomarkers. A Review From the Study Group on Biomarkers of the Heart Failure Association of the European Society of Cardiology,” European Journal of Heart Failure 24 (2022): 927–943. 35334137 10.1002/ejhf.2493

[7] F. Zannad , F. Alla , B. Dousset , A. Perez , and B. Pitt , “Limitation of Excessive Extracellular Matrix Turnover May Contribute to Survival Benefit of Spironolactone Therapy in Patients With Congestive Heart Failure: Insights From the Randomized Aldactone Evaluation Study (RALES). Rales Investigators,” Circulation 102 (2000): 2700–2706. 11094035 10.1161/01.cir.102.22.2700

[8] A. G. Raafs , J. A. J. Verdonschot , M. Henkens , et al., “The Combination of Carboxy‐terminal Propeptide of Procollagen Type I Blood Levels and Late Gadolinium Enhancement at Cardiac Magnetic Resonance Provides Additional Prognostic Information in Idiopathic Dilated Cardiomyopathy—a Multilevel Assessment of Myocardial Fibrosis in Dilated Cardiomyopathy,” European Journal of Heart Failure 23 (2021): 933–944. 33928704 10.1002/ejhf.2201 PMC8362085

[9] Z. Wang , H. Xu , M. Chen , Y. Lu , L. Zheng , and L. Ma , “CCL24/CCR3 axis Plays a central Role in angiotensin II‐induced Heart Failure by Stimulating M2 Macrophage Polarization and Fibroblast Activation,” Cell Biology and Toxicology 39 (2023): 1413–1431. 36131165 10.1007/s10565-022-09767-5 PMC10425496

[10] K. E. Ng , P. J. Delaney , D. Thenet , et al., “Early Inflammation Precedes Cardiac Fibrosis and Heart Failure in Desmoglein 2 Murine Model of Arrhythmogenic Cardiomyopathy,” Cell and Tissue Research 386 (2021): 79–98. 34236518 10.1007/s00441-021-03488-7 PMC8526453

[11] X. Fu , A. Iglesias‐Álvarez DGarcía‐Campos , et al., “Enhanced Levels of Adiposity, Stretch and Fibrosis Markers in Patients With Coexistent Heart Failure and Atrial Fibrillation,” J Cardiovasc Transl Res 17 (2024): 13–23. 37878196 10.1007/s12265-023-10454-x

[12] X. Jiang , K. Zhang , C. Gao , et al., “Activation of FMS‐Like Tyrosine Kinase 3 Protects Against Isoprenaline‐induced Cardiac Hypertrophy by Improving Autophagy and Mitochondrial Dynamics,” Faseb Journal 36 (2022): e22672. 36440960 10.1096/fj.202200419RR

[13] W. Ma , F. Liang , H. Zhan , et al., “Activated FMS‐Like Tyrosine Kinase 3 Ameliorates Angiotensin II‐induced Cardiac Remodelling,” Acta Physiol (Oxf) 230 (2020): e13519. 32480429 10.1111/apha.13519

[14] W.‐K. Wang , B. Wang , X.‐H. Cao , and Y.‐S. Liu , “Spironolactone Alleviates Myocardial Fibrosis via Inhibition of Ets‐1 in Mice With Experimental Autoimmune Myocarditis,” Exp Ther Med 23 (2022): 369. 35495592 10.3892/etm.2022.11296 PMC9019666

[15] A. P. Harvey , A. C. Montezano , K. Y. Hood , et al., “Vascular Dysfunction and Fibrosis in Stroke‐prone Spontaneously Hypertensive Rats: The Aldosterone‐mineralocorticoid Receptor‐Nox1 Axis,” Life Sciences 179 (2017): 110–119. 28478264 10.1016/j.lfs.2017.05.002 PMC5446265

[16] J. Z. Shen , J. Morgan , G. H. Tesch , P. J. Fuller , and M. J. Young , “CCL2‐dependent Macrophage Recruitment Is Critical for Mineralocorticoid Receptor‐mediated Cardiac Fibrosis, Inflammation, and Blood Pressure Responses in Male Mice,” Endocrinology 155 (2014): 1057–1066. 24428529 10.1210/en.2013-1772

[17] H. Akhtar , H. Al Sudani , F. M. U. N. HusseinM , and K. Elkholy , “Effects of Renin‐angiotensin‐aldosterone System Inhibition on Left Ventricular Hypertrophy, Diastolic Function, and Functional Status in Patients With Hypertrophic Cardiomyopathy: A Systematic Review,” Cureus 14 (2022): e26642. 35949750 10.7759/cureus.26642 PMC9356743

[18] A. A. Mansour , F. Krautter , Z. Zhi , A. J. Iqbal , and C. Recio , “The Interplay of Galectins‐1, ‐3, and ‐9 in the Immune‐inflammatory Response Underlying Cardiovascular and Metabolic Disease,” Cardiovasc Diabetol 21 (2022): 253. 36403025 10.1186/s12933-022-01690-7 PMC9675972

[19] N. Matsumoto , S. Katoh , S. Yanagi , et al., “A Possible Role of Galectin‐9 in the Pulmonary Fibrosis of Patients With Interstitial Pneumonia,” Lung 191 (2013): 191–198. 23321864 10.1007/s00408-012-9446-0

[20] N. Zhang , X. Li , J. Wang , et al., “Galectin‐9 Regulates Follicular Helper T Cells to Inhibit Humoral Autoimmunity‐induced Pulmonary Fibrosis,” Biochemical and Biophysical Research Communications 534 (2021): 99–106. 33316546 10.1016/j.bbrc.2020.11.097

[21] Y. A. Hsu , C. Y. Chang , J. L. Lan , et al., “Amelioration of Bleomycin‐induced Pulmonary Fibrosis via TGF‐β‐induced Smad and Non‐Smad Signaling Pathways in Galectin‐9‐deficient Mice and Fibroblast Cells,” Journal of Biomedical Science 27 (2020): 24. 31937306 10.1186/s12929-020-0616-8 PMC6961390

[22] K. Lv , W. Xu , C. Wang , T. Niki , M. Hirashima , and S. Xiong , “Galectin‐9 Administration Ameliorates CVB3 Induced Myocarditis by Promoting the Proliferation of Regulatory T Cells and Alternatively Activated Th2 Cells,” Clinical Immunology 140 (2011): 92–101. 21507728 10.1016/j.clim.2011.03.017

[23] J. W. Lee , J. E. Oh , K. J. Rhee , et al., “Co‐treatment With Interferon‐γ and 1‐methyl Tryptophan Ameliorates Cardiac Fibrosis Through Cardiac Myofibroblasts Apoptosis,” Molecular and Cellular Biochemistry 458 (2019): 197–205. 31006829 10.1007/s11010-019-03542-7 PMC6616223

[24] A. Kimura , Y. Ishida , M. Furuta , et al., “Protective Roles of Interferon‐γ in Cardiac Hypertrophy Induced by Sustained Pressure Overload,” Journal of the American Heart Association 7 (2018): e008145. 29555642 10.1161/JAHA.117.008145 PMC5907566

[25] B. Schreier , S. Rabe , B. Schneider , et al., “Aldosterone/NaCl‐induced Renal and Cardiac Fibrosis Is Modulated by TGF‐β Responsiveness of T Cells,” Hypertension Research 34 (2011): 623–629. 21346767 10.1038/hr.2011.16

[26] P. Nan , X. Dong , X. Bai , et al., “Tumor–stroma TGF‐β1–THBS2 Feedback Circuit Drives Pancreatic Ductal Adenocarcinoma Progression via Integrin αvβ3/CD36‐mediated Activation of the MAPK Pathway,” Cancer Letters 528 (2022): 59–75. 34958892 10.1016/j.canlet.2021.12.025

[27] J. H. C. Mak , D. T. W. Lui , C. H. Y. Fong , et al., “Serum Thrombospondin‐2 Level Changes With Liver Stiffness Improvement in Patients With Type 2 Diabetes,” Clinical Endocrinology 100 (2024): 230–237. 38127469 10.1111/cen.15010

[28] K. P. Kresoja , K. P. Rommel , R. Wachter , et al., “Proteomics to Improve Phenotyping in Obese Patients With Heart Failure With Preserved Ejection Fraction,” European Journal of Heart Failure 23 (2021): 1633–1644. 34231954 10.1002/ejhf.2291

[29] N. Girerd , D. Levy , K. Duarte , et al., “Protein Biomarkers of New‐onset Heart Failure: Insights From the Heart Omics and Ageing Cohort, the Atherosclerosis Risk in Communities Study, and the Framingham Heart Study,” Circ Heart Fail 16 (2023): E009694. 37192292 10.1161/CIRCHEARTFAILURE.122.009694 PMC10179982

[30] D. A. Chistiakov , A. A. Melnichenko , V. A. Myasoedova , A. V. Grechko , and A. N. Orekhov , “Thrombospondins: A Role in Cardiovascular Disease,” International Journal of Molecular Sciences 18 (2017): 1540. 28714932 10.3390/ijms18071540 PMC5536028

[31] Y. Kimura , Y. Izumiya , S. Hanatani , et al., “High Serum Levels of Thrombospondin‐2 Correlate With Poor Prognosis of Patients With Heart Failure With Preserved Ejection Fraction,” Heart and Vessels 31 (2016): 52–59. 25150586 10.1007/s00380-014-0571-y

[32] S. Hanatani , Y. Izumiya , S. Takashio , et al., “Circulating Thrombospondin‐2 Reflects Disease Severity and Predicts Outcome of Heart Failure With Reduced Ejection Fraction,” Circulation Journal 78 (2014): 903–910. 24500070 10.1253/circj.cj-13-1221

[33] C. Lee , M. Wu , D. Lui , et al., “Prospective Associations of Circulating Thrombospondin‐2 Level With Heart Failure Hospitalization, Left Ventricular Remodeling and Diastolic Function in Type 2 Diabetes,” Cardiovasc Diabetol 21 (2022): 231. 36335340 10.1186/s12933-022-01646-x PMC9637303

[34] P. Lacolley , M. E. Safar , B. Lucet , K. Ledudal , C. Labat , and A. Benetos , “Prevention of Aortic and Cardiac Fibrosis by Spironolactone in Old Normotensive Rats,” Journal of the American College of Cardiology 37 (2001): 662–667. 11216994 10.1016/s0735-1097(00)01129-3

[35] C. J. Leader , G. T. Wilkins , and R. J. Walker , “The Effect of Spironolactone on Cardiac and Renal Fibrosis Following Myocardial Infarction in Established Hypertension in the Transgenic Cyp1a1Ren2 Rat,” PLoS ONE 16 (2021): e0260554. 34843581 10.1371/journal.pone.0260554 PMC8629264

[36] R. Palacios‐Ramirez , M. Soulié , A. Fernandez‐Celis , et al., “Mineralocorticoid Receptor (MR) Antagonist Eplerenone and MR Modulator Balcinrenone Prevent Renal Extracellular Matrix Remodeling and Inflammation via the MR/Proteoglycan/TLR4 Pathway,” Clinical Science (London, England: 1979) 138 (2024): 1025–1038. 39092535 10.1042/CS20240302

[37] F. Edelmann , R. Wachter , A. G. Schmidt , et al., “Effect of Spironolactone on Diastolic Function and Exercise Capacity in Patients With Heart Failure With Preserved Ejection Fraction: The Aldo‐DHF Randomized Controlled Trial,” Jama 309 (2013): 781–791. 23443441 10.1001/jama.2013.905

[38] J. P. Ferreira , J. Verdonschot , P. Wang , et al., “Proteomic and Mechanistic Analysis of Spironolactone in Patients at Risk for HF,” JACC Heart Fail 9 (2021): 268–277. 33549556 10.1016/j.jchf.2020.11.010

[39] M. Schnelle , A. Leha , A. Eidizadeh , et al., “Plasma Biomarker Profiling in Heart Failure Patients With Preserved Ejection Fraction Before and After Spironolactone Treatment: Results From the Aldo‐DHF Trial,” Cells 10 (2021): 2796. 34685778 10.3390/cells10102796 PMC8535031

