# Supplementary material for: Spironolactone and Fibrosis in Heart Failure Risk: Machine Learning Analysis of HOMAGE Trial Plasma Proteomics
Source: MedComm (2020). 2026 Feb 17;7(3):e70634. doi: 10.1002/mco2.70634 (PMC12914075; doi:10.1002/mco2.70634)
Supplement: Supplementary file 1 — Supporting File 1: mco270634‐sup‐0001‐SuppMat.docx [file MCO2-7-e70634-s001.docx]

**Spironolactone and fibrosis in heart failure risk: Machine learning analysis of HOMAGE trial plasma proteomics**

Running title: Spironolactone and fibrosis in heart failure risk

Susana Ravassa PhD^1,2*^, Nicolas Girerd MD PhD^3^, Frank Edelman MD PhD^4,5^, Begoña López PhD^1,2^, João Pedro Ferreira MD PhD^3,6^, Daniela Zurkan MD^4,5^, Gorka San José PhD^1,2^, Iñigo Latasa BPhEd MSc^1,2^, Pierpaolo Pellicori MD^7^, Franco Cosmi MD^8^, Johannes Petutschnigg MD^5,9,10^ Stephane Heymans MD PhD^11^, Hans-Peter Brunner-La Rocca MD PhD^11^, Burkert Pieske MD^12^, Christian Delles^7^, Andrew L. Clark MD^13^, Javier Díez MD PhD^1,2^, Faiez Zannad MD PhD^3^, John G.F. Cleland MD^7^, Arantxa González PhD^1,^^2,14^* *on behalf of the HOMAGE Trial Committees and Investigators.*

^1^Laboratory of Heart Failure, CIMA Universidad de Navarra and IdiSNA, Pamplona, Spain. Emails: S.R, [sravassa@unav.es](mailto:sravassa@unav.es); B.L., [blopez@unav.es](mailto:blopez@unav.es); G.S.J., [gsanjose@unav.es](mailto:gsanjose@unav.es); I.L., [ilatasaamez@alumni.unav.es](mailto:ilatasaamez@alumni.unav.es); J.D., [jadimar@unav.es](mailto:jadimar@unav.es).

^2^CIBERCV, Carlos III Institute of Health, Madrid, Spain.

^3^Université de Lorraine, Inserm, Centre d'Investigation Clinique Plurithématique 1433, U1116, CHRU de Nancy, F-CRIN INI-CRCT, Nancy, France. Emails: N.G., [n.girerd@chru-nancy.fr](mailto:n.girerd@chru-nancy.fr); F.Z., [f.zannad@chru-nancy.fr](mailto:f.zannad@chru-nancy.fr);

^4^Department of Internal Medicine and Cardiology Campus Virchow Klinikum, Charité University Medicine Berlin. Emails: F.E., [frank.edelmann@dhzc-charite.de](mailto:frank.edelmann@dhzc-charite.de); D.Z., [daniela.zurkan@dhzc-charite.de](mailto:daniela.zurkan@dhzc-charite.de);

^5^DZHK (German Centre for Cardiovascular Research), partner site Berlin, Germany. Emails: J.P., [johannes.petutschnigg@dhzc-charite.de](mailto:johannes.petutschnigg@dhzc-charite.de).

^6^Cardiovascular Research and Development Center, Department of Surgery and Physiology, Faculty of Medicine of the University of Porto, Porto, Portugal. Email: J.P.F., [jp7ferreira@hotmail.com](mailto:jp7ferreira@hotmail.com).

^7^School of Cardiovascular and Metabolic Health, University of Glasgow, Glasgow, UK. Emails: P.P., [pierpaolo.pellicori@glasgow.ac.uk](mailto:pierpaolo.pellicori@glasgow.ac.uk); C.D., [Christian.Delles@glasgow.ac.uk](mailto:Christian.Delles@glasgow.ac.uk); J.G.F.C., [John.Cleland@glasgow.ac.uk](mailto:John.Cleland@glasgow.ac.uk).

^8^Department of Cardiology, Cortona Hospital, Arezzo, Italy. Email: francocosmi@virgilio.it.

^9^Deutsches Herzzentrum der Charité, Klinik für Kardiologie, Angiologie & Intensivmedizin, Augustenburger Platz 1, 13353 Berlin, Germany.

^10^Charité – Universitätsmedizin Berlin, corporate member of Freie Universität Berlin and Humboldt-Universität zu Berlin, Charitéplatz 1, 10117 Berlin, Germany.

^11^Department of Cardiology, Maastricht University Medical Center, the Netherlands. Emails: S.H., [stephane.heymans@mumc.nl](mailto:stephane.heymans@mumc.nl); H.P.B.L.R., hp.brunnerlarocca@mumc.nl.

^12^Division of Cardiology, Department of Internal Medicine, University Medicine Rostock, Schillingallee 35, 18057 Rostock, Germany. Email: B.P., [burkert.pieske@t-online.de](mailto:burkert.pieske@t-online.de).

^13^Hull University Teaching Hospitals NHS Trust, Castle Hill Hospital, Cottingham, HU16 5JQ, UK. Email: A.L.C., a.l.clark@hull.ac.uk.

^14^Department of Cardiology and Cardiac Surgery, Clínica Universidad de Navarra, Pamplona, Spain. Email: A.G., amiqueo@unav.es.

* Authors for correspondence: Arantxa González (amiqueo@unav.es) or Susana Ravassa (sravassa@unav.es), Laboratory of Heart Failure, CIMA Universidad de Navarra.

**METHODS**

**Study Populations**

HOMAGE trial

The main inclusion criteria were age 60 years or older, increased risk of cardiac dysfunction (defined by the presence of coronary artery disease or at least two of the following: diabetes, treated hypertension, microalbuminuria, abnormal electrocardiogram), and evidence of cardiac dysfunction as previously defined ^1^. Exclusion criteria included estimated glomerular filtration rate (eGFR) <30 mL/min/1.73m^2^, serum potassium >5.0 mmol/L, LV ejection fraction <45%, a previous diagnosis of HF or treatment with loop diuretics and atrial fibrillation/flutter before randomization ^1^.

Aldo-DHF trial

The main inclusion criteria were stable HFpEF defined as New York Heart Association (NYHA) class II or III HF symptoms, LV ejection fraction (LVEF) ≥50% at rest, echocardiographic evidence of grade≥I diastolic dysfunction or atrial fibrillation, and peak oxygen consumption ≤25 mL/kg/min. Major exclusion criteria included: prior documented LVEF ≤40%, significant coronary artery disease, myocardial infarction or coronary artery bypass graft surgery within three months, definitive or probable pulmonary disease [vital capacity <80% or forced expiratory volume in 1 s (FEV1) <80% or reference values on spirometry], body mass index ≥36 kg/m2, or serum creatinine >1.8 mg/dL ^2^.

**Biomarkers**

Serum PICP and galectin3 were measured using an enzyme-linked immune-assays (Quidel Corporation and BG Medicine, respectively), PIIINP was measured by radio-immunoassay (Orion Diagnostica), and NT-proBNP, sensitivity troponin T (hs-TnT) and growth differentiation factor 15 (GDF-15) were measured by electro-chemiluminescent assays (Roche diagnostics) as previously described.^1-3^

**Machine learning analyses**

Patients from the HOMAGE trial were randomly assigned to training (80%) and test (20%) sets, and the machine learning (ML) algorithms artificial neural network (ANN, R (v4.4.2) packages *Keras* (v2.15.0)^4^ and *Tensorflow* (v2.16.0)^5^ as interfaces to these packages in Python environment), conditional random forest (cRF. R package *party,* v1.3-17)^6^, extreme gradient boosting (XGB, R package *xgboost*, v1.7.8.1)^7^ and linear elastic-net penalized regression (ENR, R package *glmnet*, v4.1-8)^8^ were performed. These ML algorithms were chosen as methods accommodating linear and non-linear relationships as well as feature interactions, including those with the highest accuracy (ANN, cRF and XGB) and interpretability (ENR).^9-11^ PICP fold-change (final/baseline values) was the continuous dependent variable, and the Olink protein baseline and fold-change (2^final/2^baseline NPX) values, baseline PICP and the variables age, sex, current smoker, diabetes mellitus (DM), coronary artery disease (CAD) and QRS, as well as the baseline and fold-change values of body mass index, systolic and diastolic blood pressure, serum sodium and potassium, eGFR and breathlessness scale, were considered as predictors (Supplemental Figure 1). These associations were explored both at one- and nine-months visits. Subjects with ≥50% missing values were excluded. In the remaining patients with missing values (<2% per patient) multivariate imputations by chained equations were performed with the R package *mice* (v3.17.0)^12^ (Supplemental Figure 1). Optimal normalization was achieved by rescaling the data within the range 0-1.

For ML model development, we applied 3-fold cross-validation and grid search to achieve hyperparameter optimization and to prevent overfitting. The optimal hyperparameter set for each algorithm is shown in the supplemental Table 7. For ANN, the different architectures were trained by using the R package *tfruns* (v1.5.3).^13^ We used the Rectified Linear Unit (ReLU) as the nonlinear activation function and dropout was not required for optimization. For the hyperparameters not mentioned in the supplemental Table 7, the default values of the respective software R packages were used. Evaluation of algorithms was performed in terms of minimization of the RMSE and maximization of the R^2^ on the test set. Variable importance was computed following a 1000 permutation-based approach and represented in bar charts (the longer the bar chart, the higher the importance of the feature in each ML algorithm), and partial dependence profiles, showing how the expected value of a model prediction changes as a function of a feature, were obtained using the *DALEX* (v2.4.3)^14^ R package. Variables were considered relevant factors associated with PICP if selected in at least two of the four ML models, at one and nine months, to increment the probability that the selected features are robust and not just artifacts of a single model. Performance and visualization of the correlation (Pearson) heatmap was performed using the *heatmap.2* function of the R package *gplots* (v3.2.0).^15^

**Function enrichment analyses**

Gene ontology (GO) and Kyoto Encyclopedia of Genes and Genomes analysis (KEGG) were conducted using the *clusterProfiler* R package (v4.12.6).^16^ The *ggplot2* R package (v3.5.1)^17^ was used for visual mapping. The human genome, and the list of all proteins examined, were used as background references. The terms were considered significant if their associated p and q values were <0.05 (Benjamin-Hochberg method). The *enrichplot* R package (v1.24.4)^18^ was used to draw bubble charts and cnetplots.

**Statistical analyses**

Mann-Whitney-Wilcoxon and Chi-square (or Fisher’s exact test as required) tests were performed to analyze differences between two groups of continuous and categorical variables, respectively.

Interaction analyses were performed to test the influence of spironolactone on the slopes of the PICP fold-change regressed on the ML-selected variables. The linear regression models included the randomization variable, the ML-selected protein fold-change, their interaction term, eGFR fold-change, and the baseline PICP, ML-selected protein and eGFR. If non-linearity was found, polynomial or restricted cubic spline terms were used, confirming their degree by 10-fold cross-validation and further evaluation of goodness of fit with the likelihood ratio test. The effect of spironolactone on the circulating levels of the ML-selected proteins at one and nine months (dependent variables) was assessed using mixed effect models with the randomization variable, the respective baseline protein levels and the eGFR fold change values as fixed-effects variables; the random intercepts were set at the patient “ID” levels and if the model performed better using “visit” as random slope, it was included in addition to the random intercept. The variance component structure was specified according to the best-fit model determined by the likelihood ratio test. The heteroscedasticity and normality of the residuals were examined by graphic analysis of the scatterplots and Q-Q plots, respectively. If outlying residual observations were found, analyses were confirmed by robust regression (R package *robustbase*, v0.99-4-1).^19^ No multicollinearity, as defined by a variance inflation factor greater than 5, was detected.

**References**

1. Cleland JGF, Ferreira JP, Mariottoni B, et al. The effect of spironolactone on cardiovascular function and markers of fibrosis in people at increased risk of developing heart failure: the heart ‘OMics’ in AGEing (HOMAGE) randomized clinical trial. *Eur Heart J* 2021;42:684–696.

2. Edelmann F, Wachter R, Schmidt AG, et al. Effect of spironolactone on diastolic function and exercise capacity in patients with heart failure with preserved ejection fraction: the Aldo-DHF randomized controlled trial. *JAMA* 2013;309:781–791.

3. Ravassa S, Trippel T, Bach D, et al. Biomarker-based phenotyping of myocardial fibrosis identifies patients with heart failure with preserved ejection fraction resistant to the beneficial effects of spironolactone: results from the Aldo-DHF trial. Eur *J Heart Fail*. 2018;20:1290–1299.

4. Kalinowski T, Allaire J, Chollet F (2024). keras3: R Interface to 'Keras'. R package version 1.2.0, https://github.com/rstudio/keras3.

5. Allaire J, Tang Y (2024). tensorflow: R Interface to 'TensorFlow'. R package version 2.16.0.9000, <https://github.com/rstudio/tensorflow>.

6. Strobl C, Boulesteix AL, Kneib T, Augustin T, Zeileis A. Conditional variable importance for random forests. *BMC Bioinformatics*. 2008;9:307. doi: 10.1186/1471-2105-9-307.

7. Chen T, He T, Benesty M, Khotilovich V, Tang Y, Cho H, Chen K, Mitchell R, Cano I, Zhou T, Li M, Xie J, Lin M, Geng Y, Li Y, Yuan J (2024). xgboost: Extreme Gradient Boosting. R package version 1.7.8.1, <https://CRAN.R-project.org/package=xgboost>.

8. Tay JK, Narasimhan B, Hastie T. Elastic Net Regularization Paths for All Generalized Linear Models. *J Stat Softw*. 2023;106:1. doi: 10.18637/jss.v106.i01.

9. Crivello JC, Joubert JM, Sokolovska N. Supervised deep learning prediction of the formation enthalpy of complex phases using a DFT database: The σ−phase as an example. *Comput Mater Sci* 2022;201.

10. Viswan V, Shaffi N, Mahmud M, Subramanian K, Hajamohideen F. Explainable Artificial Intelligence in Alzheimer’s Disease Classification: A Systematic Review. *Cognitive Computation* 2024;16:1–44.

11. Oikonomou EK, Khera R. Machine learning in precision diabetes care and cardiovascular risk prediction. *Cardiovasc Diabetol* 2023;22:259.

12. van Buuren S, Groothuis-Oudshoorn K (2011). mice: Multivariate Imputation by Chained Equations in R. *Journal of Statistical Software* 2011;45:1-67. DOI 10.18637/jss.v045.i03.

13. Allaire J (2024). tfruns: Training Run Tools for 'TensorFlow'. R package version 1.5.3, <https://github.com/rstudio/tfruns>.

14. Biecek P. “DALEX: Explainers for Complex Predictive Models in R*. Journal of Machine Learning Research* 2018;19(84):1-5. <https://jmlr.org/papers/v19/18-416.html>

15. Warnes G, Bolker B, Bonebakker L, Gentleman R, Huber W, Liaw A, Lumley T, Maechler M, Magnusson A, Moeller S, Schwartz M, Venables B, Galili T (2024). gplots: Various R Programming Tools for Plotting Data. R package version 3.2.0, , <https://github.com/talgalili/gplots>.

16. Xu S, Hu E, Cai Y, Xie Z, Luo X, Zhan L, Tang W, Wang Q, Liu B, Wang R, Xie W, Wu T, Xie L, Yu G. Using clusterProfiler to characterize multiomics data. *Nat Protoc*. 2024;19:3292-3320. doi: 10.1038/s41596-024-01020-z.

17. H. Wickham. ggplot2: Elegant Graphics for Data Analysis. Springer-Verlag New York, 2016.

18. Yu G (2024). enrichplot: Visualization of Functional Enrichment Result. R package version 1.24.4, <https://bioconductor.org/packages/enrichplot>.

19. Maechler M, Rousseeuw P, Croux C, Todorov V, Ruckstuhl A, Salibian-Barrera M, Verbeke T, Koller M, Conceicao EL, Anna di Palma M (2024). robustbase: Basic Robust Statistics. R package version 0.99-4-1, <http://robustbase.r-forge.r-project.org/>.

**Supplemental table 1. Protein names and respective Olink® panel sorted in alphabetical order**

| **Protein full name** | **Entry name** | **Olink® Panel*** | **Uniprot ID**** |
| --- | --- | --- | --- |
| Angiotensin-converting enzyme 2 | ACE2 | CV II | Q9BYF1 |
| Adenosine Deaminase | ADA | INF | P00813 |
| Adisintegrin and metalloproteinase with thrombospondin motifs 13 | ADAMTS13 | CV II | Q76LX8 |
| ADM | ADM | CV II | P35318 |
| Agouti-related protein | AGRP | CV II | O00253 |
| CD166 antigen | ALCAM | CV III | Q13740 |
| Protein AMBP | AMBP | CV II | P02760 |
| Angiopoietin-1 | ANG1 | CV II | Q15389 |
| Aminopeptidase N | APN | CV III | P15144 |
| Axin-1 | AXIN1 | INF | O15169 |
| Tyrosine-protein kinase receptor UFO | AXL | CV III | P30530 |
| Azurocidin | AZU1 | CV III | P20160 |
| Brain-derived neurotrophic factor | BDNF | INF | P23560 |
| Beta-nerve growth factor | BETANGF | INF | P01138 |
| Bleomycin hydrolase | BLMHYDROLASE | CV III | Q13867 |
| Bone morphogenetic protein 6 | BMP6 | CV II | P22004 |
| Natriuretic peptides B | BNP | CV II | P16860 |
| Eukaryotic translation initiation factor 4E-binding protein 1 | BP1_4E | INF | Q13541 |
| Carbonic anhydrase 5A, mitochondrial | CA5A | CV II | P35218 |
| Caspase-3 | CASP3 | CV III | P42574 |
| Caspase 8 | CASP8 | INF | Q14790 |
| Eotaxin-1 | CCL11 | INF | P51671 |
| C-C motif chemokine 15 | CCL15 | CV III | Q16663 |
| C-C motif chemokine 16 | CCL16 | CV III | O15467 |
| C-C motif chemokine 17 | CCL17 | CV II | Q92583 |
| C-C motif chemokine 19 | CCL19 | INF | Q99731 |
| C-C motif chemokine 20 | CCL20 | INF | P78556 |
| C-C motif chemikine 22 | CCL22 | CV III | O00626 |
| C-C motif chemokine 23 | CCL23 | INF | P55773 |
| C-C motif chemokine 24 | CCL24 | CV III | O00175 |
| C-C motif chemokine 25 | CCL25 | INF | O15444 |
| C-C motif chemokine 28 | CCL28 | INF | Q9NRJ3 |
| C-C motif chemokine 3 | CCL3 | CV II | P10147 |
| C-C motif chemokine 4 | CCL4 | INF | P13236 |
| Scavenger receptor cysteine-rich type 1 protein M130 | CD163 | CV III | Q86VB7 |
| Natural killer cell receptor 2B4 | CD244 | INF | Q9BZW8 |
| T-cell surface glycoprotein CD4 | CD4 | CV II | P01730 |
| CD40L receptor | CD40 | INF | P25942 |
| CD40 ligand | CD40L | CV II | P29965 |
| T-cell surface glycoprotein CD5 | CD5 | INF | P06127 |
| T cell surface glycoprotein CD6 | CD6 | INF | P30203 |
| SLAM family member 5 | CD84 | CV II | Q9UIB8 |
| Complement component C1q receptor | CD93 | CV III | Q9NPY3 |
| CUB domain-containing protein 1 | CDCP1 | INF | Q9H5V8 |
| Cadherin-5 | CDH5 | CV III | P33151 |
| Carcinoembryonic antigenrelated cell adhesion molecule 8 | CEACAM8 | CV II | P31997 |
| Chitinase-3-like protein 1 | CHI3L1 | CV III | P36222 |
| Chitotriosidase-1 | CHIT1 | CV III | Q13231 |
| Contactin-1 | CNTN1 | CV III | Q12860 |
| Collagen alpha-1(I) chain | COL1A1 | CV III | P02452 |
| Carboxypeptidase A1 | CPA1 | CV III | P15085 |
| Carboxypeptidase B | CPB1 | CV III | P15086 |
| Macrophage colony-stimulating factor 1 | CSF1 | INF | P09603 |
| Cystatin D | CST5 | INF | P28325 |
| Cystatin-B | CSTB | CV III | P04080 |
| Chymotrypsin C | CTRC | CV II | Q99895 |
| Cathepsin D | CTSD | CV III | P07339 |
| Cathepsin L1 | CTSL1 | CV II | P07711 |
| Cathepsin Z | CTSZ | CV III | Q9UBR2 |
| Fractalkine | CX3CL1 | INF | P78423 |
| C-X-C motif chemokine 1 (CV2) | CXCL1 | CV II | P09341 |
| C-X-C motif chemokine 10 | CXCL10 | INF | P02778 |
| C-X-C motif chemokine 11 | CXCL11 | INF | O14625 |
| C-X-C motif chemokine 16 | CXCL16 | CV III | Q9H2A7 |
| C-X-C motif chemokine 5 | CXCL5 | INF | P42830 |
| C-X-C motif chemokine 6 | CXCL6 | INF | P80162 |
| C-X-C motif chemokine 9 | CXCL9 | INF | Q07325 |
| Decorin | DCN | CV II | P07585 |
| 2,4-dienoyl-CoA reductase, mitochondrial | DECR1 | CV II | Q16698 |
| Dickkopf-related protein 1 | DKK1 | CV II | O94907 |
| Azurocidin | DLK1 | CV III | P80370 |
| Delta and Notch-like epidermal growth factor-related receptor | DNER | INF | Q8NFT8 |
| Epidermal growth factor receptor | EGFR | CV III | P00533 |
| Protein S100-A12 | ENRAGE | INF | P80511 |
| Epithelial cell adhesion molecule | EPCAM | CV III | P16422 |
| Ephrin type-B receptor 4 | EPHB4 | CV III | P54760 |
| Fatty acid-binding protein, intestinal | FABP2 | CV II | P12104 |
| Fatty acid-binding protein, adipocyte | FABP4 | CV III | P15090 |
| Tumor necrosis factor receptor superfamily member 6 | FAS | CV III | P25445 |
| Fibroblast growth factor 19 | FGF19 | INF | O95750 |
| Fibroblast growth factor 21 (CV2) | FGF21 | CV II | Q9NSA1 |
| Fibroblast growth factor 23 (CV2) | FGF23 | CV II | Q9GZV9 |
| Fibroblast growth factor 5 | FGF5 | INF | P12034 |
| Fms-related tyrosine kinase 3 ligand | FLT3L | INF | P49771 |
| Follistatin | FS | CV II | P19883 |
| Galectin-3 | GAL3 | CV III | P17931 |
| Galectin-4 | GAL4 | CV III | P56470 |
| Galectin-9 | GAL9 | CV II | O00182 |
| Growth/differentiation factor 15 | GDF15 | CV III | Q99988 |
| Growth/differentiation factor 2 | GDF2 | CV II | Q9UK05 |
| Growth hormone | GH | CV II | P01241 |
| Gastric intrinsic factor | GIF | CV II | P27352 |
| Lactoylglutathione lyase | GLO1 | CV II | Q04760 |
| Granulins | GRN | CV III | P28799 |
| Gastrotropin | GT | CV II | P51161 |
| Hydroxyacid oxidase 1 | HAOX1 | CV II | Q9UJM8 |
| Proheparin-binding EGF-like growth factor | HBEGF | CV II | Q99075 |
| Glial cell line-derived neurotrophic factor | HGDNF | INF | P39905 |
| Hepatocyte growth factor | HGF | INF | P14210 |
| Heme oxygenase 1 | HO1 | CV II | P09601 |
| Osteoclast-associated immunoglobulin-like receptor | HOSCAR | CV II | Q8IYS5 |
| Heat shock 27 kDa protein | HSP27 | CV II | P04792 |
| Intercellular adhesion molecule 2 | ICAM2 | CV III | P13598 |
| Alpha-L-iduronidase | IDUA | CV II | P35475 |
| Insulin-like growth factor-binding protein 1 | IGFBP1 | CV III | P08833 |
| Insulin-like Growth Factor-Binding Protein 2 | IGFBP2 | CV III | P18065 |
| Insulin-like growth factor-binding protein 7 | IGFBP7 | CV III | Q16270 |
| Low affinity immunoglobulin gamma Fc region receptor II-b | IGGFCRECEPTORIIB | CV II | P31994 |
| Interleukin-10 | IL10 | INF | P22301 |
| Interleukin-10 receptor subunit alpha | IL10RA | INF | Q13651 |
| Interleukin-10 receptor subunit beta | IL10RB | INF | Q08334 |
| Interleukin-12 subunit beta | IL12B | INF | P29460 |
| Interleukin-13 | IL13 | INF | P35225 |
| Interleukin-15 receptor subunit alpha | IL15RA | INF | Q13261 |
| Pro-interleukin-16 | IL16 | CV II | Q14005 |
| Interleukin-17A | IL17A | INF | Q16552 |
| Interleukin-17C | IL17C | INF | Q9P0M4 |
| Interleukin-17D | IL17D | CV II | Q8TAD2 |
| Interleukin-17 receptor A | IL17RA | CV III | Q96F46 |
| Interleukin-18 (CV2) | IL18 | CV II | Q14116 |
| Interleukin-18-binding protein | IL18BP | CV III | O95998 |
| Interleukin-18 receptor 1 | IL18R1 | INF | Q13478 |
| Interleukin-1 receptor antagonist protein | IL1RA | CV II | P18510 |
| Interleukin-1 receptor-like 2 | IL1RL2 | CV II | Q9HB29 |
| Interleukin-1 receptor type 1 | IL1RT1 | CV III | P14778 |
| Interleukin-1 receptor type 2 | IL1RT2 | CV III | P27930 |
| Interleukin-20 receptor subunit alpha | IL20RA | INF | Q9UHF4 |
| Interleukin-27 | IL27 | CV II | Q8NEV9 |
| Interleukin-2 receptor subunit alpha | IL2RA | CV III | P01589 |
| Interleukin-4 receptor subunit alpha | IL4RA | CV II | P24394 |
| Interleukin-6 (CV2) | IL6 | CV II | P05231 |
| Interleukin-6 receptor subunit alpha | IL6RA | CV III | P08887 |
| Interleukin-7 | IL7 | INF | P13232 |
| Interleukin-8 | IL8 | INF | P10145 |
| Melusin | ITGB1BP2 | CV II | Q9UKP3 |
| Integrin beta-2 | ITGB2 | CV III | P05107 |
| Junctional adhesion molecule A | JAMA | CV III | Q9Y624 |
| Kidney injury molecule 1 | KIM1 | CV II | Q96D42 |
| Kallikrein-6 | KLK6 | CV III | Q92876 |
| Latency-associated peptide transforming growth factor beta 1 | LAPTGFBETA1 | INF | P01137 |
| Low-density lipoprotein receptor | LDLRECEPTOR | CV III | P01130 |
| Leptin | LEP | CV II | P41159 |
| Leukemia inhibitory factor receptor | LIFR | INF | P42702 |
| Lectin-like oxidized LDL receptor 1 | LOX1 | CV II | P78380 |
| Lipoprotein lipase | LPL | CV II | P06858 |
| Lymphotoxin-beta receptor | LTBR | CV III | P36941 |
| Macrophage receptor MARCO | MARCO | CV II | Q9UEW3 |
| Myoglobin | MB | CV III | P02144 |
| Monocyte chemotactic protein 1 | MCP1_CV3 | CV III | P13500 |
| Monocyte chemotactic protein 2 | MCP2 | INF | P80075 |
| Monocyte chemotactic protein 3 | MCP3 | INF | P80098 |
| Monocyte chemotactic protein 4 | MCP4 | INF | Q99616 |
| Matrix extracellular phosphoglycoprotein | MEPE | CV III | Q9NQ76 |
| Tyrosine-protein kinase Mer | MERTK | CV II | Q12866 |
| Matrix metalloproteinase-1 | MMP1 | INF | P03956 |
| Matrix metalloproteinase-10 | MMP10 | INF | P09238 |
| Matrix metalloproteinase-12 | MMP12 | CV II | P39900 |
| Matrix metalloproteinase-2 | MMP2 | CV III | P08253 |
| Matrix metalloproteinase-3 | MMP3 | CV III | P08254 |
| Matrix metalloproteinase-7 | MMP7 | CV II | P09237 |
| Matrix metalloproteinase-9 | MMP9 | CV III | P14780 |
| Myeloperoxidase | MPO | CV III | P05164 |
| NF-kappa-B essential modulator | NEMO | CV II | Q9Y6K9 |
| Neurogenic locus notch homolog protein 3 | NOTCH3 | CV III | Q9UM47 |
| Neurotrophin-3 | NT3 | INF | P20783 |
| N-terminal prohormone brain natriuretic peptide | NTPROBNP | CVII | P16860 |
| Osteoprotegerin | OPG_CV3 | CV III | O00300 |
| Osteopontin | OPN | CV III | P10451 |
| Oncostatin-M | OSM | INF | P13725 |
| Plasminogen activator inhibitor 1 | PAI | CV III | P05121 |
| Pappalysin-1 | PAPPA | CV II | Q13219 |
| Proteinase-activated receptor 1 | PAR1 | CV II | P25116 |
| Poly [ADP-ribose] polymerase 1 | PARP1 | CV II | P09874 |
| Proprotein convertase subtilisin/kexin type 9 | PCSK9 | CV III | Q8NBP7 |
| Platelet-derived growth factor subunit A | PDGFSUBUNITA | CV III | P04085 |
| Platelet-derived growth factor subunit B | PDGFSUBUNITB | CV II | P01127 |
| Programmed cell death 1 ligand 1 | PDL1 | INF | Q9NZQ7 |
| Programmed cell death 1 ligand 2 | PDL2 | CV II | Q9BQ51 |
| Platelet endothelial cell adhesion molecule | PECAM1 | CV III | P16284 |
| Peptidoglycan recognition protein 1 | PGLYRP1 | CV III | O75594 |
| Elafin | PI3 | CV III | P19957 |
| Polymeric immunoglobulin receptor | PIGR | CV II | P01833 |
| Perlecan | PLC | CV III | P98160 |
| Placenta growth factor | PLGF | CV II | P49763 |
| Paraoxonase (PON 3) | PON3 | CV III | Q15166 |
| Prolargin | PRELP | CV II | P51888 |
| Brother of CDO | PROTEINBOC | CV II | Q9BWV1 |
| Serine protease 27 | PRSS27 | CV II | Q9BQR3 |
| Prostasin | PRSS8 | CV II | Q16651 |
| Myeloblastin | PRTN3 | CV III | P24158 |
| P-selectin glycoprotein ligand 1 | PSGL1 | CV II | Q14242 |
| Pulmonary surfactant-associated protein D | PSPD | CV III | P35247 |
| Pentraxin-related protein PTX3 | PTX3 | CV II | P26022 |
| Receptor for advanced glycosylation end products | RAGE | CV II | Q15109 |
| Retinoic acid receptor responder protein 2 | RARRES2 | CV III | Q99969 |
| Renin | REN | CV II | P00797 |
| Resistin | RETN | CV III | Q9HD89 |
| Stem cell factor (CV2) | SCF | CV II | P21583 |
| Secretoglobin family 3A member 2 | SCGB3A2 | CV III | Q96PL1 |
| E-selectin | SELE | CV III | P16581 |
| P-selectin | SELP | CV III | P16109 |
| Serpin A12 | SERPINA12 | CV II | Q8IW75 |
| Tyrosine-protein phosphatase non-receptor type substrate 1 | SHPS1 | CV III | P78324 |
| SIR2-like protein 2 | SIRT2 | INF | Q8IXJ6 |
| Signaling lymphocytic activation molecule | SLAMF1 | INF | Q13291 |
| SLAM family member 7 | SLAMF7 | CV II | Q9NQ25 |
| Superoxide dismutase [Mn], mitochondrial | SOD2 | CV II | P04179 |
| Sortilin | SORT1 | CV II | Q99523 |
| Spondin-1 | SPON1 | CV III | Q9HCB6 |
| Spondin-2 | SPON2 | CV II | Q9BUD6 |
| Proto-oncogene tyrosine-protein kinase Src | SRC | CV II | P12931 |
| Sulfotransferase 1A1 | ST1A1 | INF | P50225 |
| ST2 protein | ST2 | CV III | Q01638 |
| STAM-binding protein | STAMPB | INF | O95630 |
| Serine/threonine-protein kinase 4 | STK4 | CV II | Q13043 |
| Tissue factor | TF | CV II | P13726 |
| Trefoil factor 3 | TFF3 | CV III | Q07654 |
| Tissue factor pathway inhibitor | TFPI | CV III | P10646 |
| Transforming growth factor alpha | TGFALPHA | INF | P01135 |
| Protein-glutamine gamma-glutamyltransferase 2 | TGM2 | CV II | P21980 |
| Thrombospondin-2 | THBS2 | CV II | P35442 |
| Thrombopoietin | THPO | CV II | P40225 |
| Angiopoietin-1 receptor | TIE2 | CV II | Q02763 |
| Metalloproteinase inhibitor 4 | TIMP4 | CV III | Q99727 |
| Trem-like transcript 2 protein | TLT2 | CV III | Q5T2D2 |
| Thrombomodulin | TM | CV II | P07204 |
| Tumor necrosis factor | TNF | INF | P01375 |
| TNF-beta | TNFB | INF | P01374 |
| Tumor necrosis factor receptor 1 | TNFR1 | CV III | P19438 |
| Tumor necrosis factor receptor 2 | TNFR2 | CV III | P20333 |
| Tumor necrosis factor receptor superfamily member 10A | TNFRSF10A | CV II | O00220 |
| Tumor necrosis factor receptor superfamily member 10C | TNFRSF10C | CV III | O14798 |
| Tumor necrosis factor receptor superfamily member 11A | TNFRSF11A | CV II | Q9Y6Q6 |
| Tumor necrosis factor receptor superfamily member 13B | TNFRSF13B | CV II | O14836 |
| Tumor necrosis factor receptor superfamily member 14 | TNFRSF14 | CV III | Q92956 |
| Tumor necrosis factor receptor superfamily member 9 | TNFRSF9 | INF | Q07011 |
| Tumor necrosis factor ligand superfamily member 13B | TNFSF13B | CV III | Q9Y275 |
| Tumor necrosis factor ligand superfamily member 14 | TNFSF14 | INF | O43557 |
| Tissue-type plasminogen activator | TPA | CV III | P00750 |
| Transferrin receptor protein 1 | TR | CV III | P02786 |
| TNF-related apoptosis-inducing ligand | TRAIL | INF | P50591 |
| TNF-related apoptosis-inducing ligand receptor 2 | TRAILR2 | CV II | O14763 |
| TNF-related activation-induced cytokine | TRANCE | INF | O14788 |
| Tartrate-resistant acid phosphatase type 5 | TRAP | CV III | P13686 |
| Tumor necrosis factor (Ligand) superfamily, member 12 | TWEAK | INF | O43508 |
| Urokinase-type plasminogen activator | UPA_CV3 | CV III | P00749 |
| Urokinase plasminogen activator surface receptor | UPAR | CV III | Q03405 |
| Vascular endothelial growth factor A | VEGFA | INF | P15692 |
| Vascular endothelial growth factor D | VEGFD | CV II | O43915 |
| V-set and immunoglobulin domain-containing protein 2 | VSIG2 | CV II | Q96IQ7 |
| von Willebrand factor | VWF | CV III | P04275 |
| Lymphotactin | XCL1 | CV II | P47992 |

CV means cardiovascular; INF, inflammation

**Supplemental table S2. Baseline protein expression by quartiles of the PICP fold-change at 1 and 9 months in patients from the HOMAGE trial**

|  | **1 Month** | | | | P trend | **9 Months** | | | | P trend |
| --- | --- | --- | --- | --- | --- | --- | --- | --- | --- | --- |
|  | Q1 (n=121) | Q2 (n=120) | Q3 (n=120) | Q4 (n=120) |  | Q1 (n=122) | Q2 (n=122) | Q3 (n=122) | Q4 (n=122) |  |
| ACE2 | 3.5 (3.1-4.0) | 3.6 (3.2-4.1) | 3.5 (3.2-3.8) | 3.4 (3.1-3.8) | 0.076 | 3.5 (3.1-4.0) | 3.6 (3.1-4.1) | 3.5 (3.2-3.8) | 3.5 (3.2-3.8) | 0.473 |
| ADA | 4.2 (3.9-4.5) | 4.1 (3.9-4.4) | 4.2 (3.9-4.4) | 4.2 (3.9-4.5) | 0.556 | 4.1 (3.8-4.3) | 4.2 (3.9-4.5) | 4.2 (4.0-4.5) | 4.2 (3.9-4.5) | 0.007 |
| ADAMTS13 | 5.7 (5.6-5.8) | 5.7 (5.5-5.8) | 5.7 (5.6-5.8) | 5.7 (5.6-5.8) | 0.868 | 5.7 (5.5-5.8) | 5.7 (5.6-5.9) | 5.7 (5.6-5.8) | 5.7 (5.6-5.8) | 0.873 |
| ADM | 7.2 (6.9-7.5) | 7.2 (6.9-7.5) | 7.2 (7.0-7.5) | 7.1 (6.9-7.3) | 0.038 | 7.1 (6.9-7.4) | 7.2 (6.9-7.5) | 7.1 (6.9-7.4) | 7.2 (7.0-7.6) | 0.312 |
| AGRP | 4.0 (3.7-4.3) | 4.0 (3.7-4.3) | 4.1 (3.8-4.4) | 3.9 (3.6-4.3) | 0.462 | 4.0 (3.7-4.3) | 4.0 (3.8-4.4) | 4.1 (3.7-4.4) | 4.0 (3.7-4.3) | 0.774 |
| ALCAM | 7.1 (6.9-7.3) | 7.1 (7.0-7.2) | 7.0 (6.9-7.3) | 7.1 (6.9-7.2) | 0.156 | 7.1 (6.9-7.2) | 7.1 (6.9-7.3) | 7.1 (6.9-7.3) | 7.1 (6.9-7.3) | 0.597 |
| AMBP | 7.5 (7.4-7.7) | 7.5 (7.4-7.7) | 7.6 (7.4-7.7) | 7.5 (7.4-7.7) | 0.293 | 7.5 (7.4-7.7) | 7.6 (7.4-7.7) | 7.5 (7.4-7.7) | 7.6 (7.4-7.7) | 0.196 |
| ANG1 | 5.9 (5.2-6.7) | 6.4 (5.6-7.2) | 6.2 (5.2-7.1) | 6.1 (5.2-6.9) | 0.886 | 6.0 (5.2-6.8) | 6.4 (5.7-7.2) | 6.0 (5.2-6.8) | 6.2 (5.2-7.1) | 0.728 |
| APN | 4.5 (4.3-4.7) | 4.5 (4.3-4.6) | 4.5 (4.3-4.7) | 4.5 (4.3-4.7) | 0.318 | 4.5 (4.3-4.6) | 4.5 (4.3-4.6) | 4.5 (4.3-4.7) | 4.5 (4.3-4.7) | 0.489 |
| ARTN | 1.0 (1.0-1.0) | 1.0 (1.0-1.0) | 1.0 (1.0-1.0) | 1.0 (1.0-1.0) | 0.098 | 1.0 (1.0-1.0) | 1.0 (1.0-1.0) | 1.0 (1.0-1.0) | 1.0 (1.0-1.0) | 0.594 |
| AXIN1 | 3.3 (2.5-4.2) | 3.7 (2.6-4.5) | 3.5 (2.4-4.4) | 3.1 (2.3-4.5) | 0.342 | 3.1 (2.3-4.1) | 3.6 (2.6-4.5) | 3.2 (2.3-4.5) | 3.3 (2.4-4.5) | 0.433 |
| AXL | 8.8 (8.6-9.0) | 8.7 (8.5-8.9) | 8.7 (8.6-9.0) | 8.7 (8.5-9.0) | 0.076 | 8.8 (8.6-9.0) | 8.7 (8.5-8.9) | 8.8 (8.5-9.0) | 8.7 (8.6-9.0) | 0.747 |
| AZU1 | 2.8 (2.8-2.8) | 2.8 (2.8-2.8) | 2.8 (2.8-2.8) | 2.8 (2.8-2.8) | 0.64 | 2.8 (2.8-2.8) | 2.8 (2.8-2.8) | 2.8 (2.8-2.8) | 2.8 (2.8-2.8) | 0.152 |
| BETANGF | 1.1 (0.9-1.3) | 1.0 (0.9-1.2) | 1.0 (0.9-1.1) | 1.1 (0.9-1.2) | 0.358 | 1.0 (0.9-1.2) | 1.1 (0.9-1.2) | 1.0 (0.9-1.2) | 1.0 (0.9-1.2) | 0.768 |
| BLMHYDRO | 1.9 (1.6-2.2) | 1.9 (1.6-2.2) | 2.0 (1.8-2.2) | 1.9 (1.7-2.2) | 0.237 | 1.9 (1.6-2.1) | 2.0 (1.7-2.3) | 1.9 (1.7-2.2) | 1.9 (1.7-2.2) | 0.155 |
| BMP6 | 4.6 (4.3-4.8) | 4.5 (4.3-4.8) | 4.5 (4.3-4.8) | 4.5 (4.3-4.8) | 0.618 | 4.6 (4.3-4.8) | 4.6 (4.3-4.9) | 4.5 (4.3-4.8) | 4.5 (4.3-4.8) | 0.154 |
| BOC | 3.9 (3.8-4.1) | 3.8 (3.7-4.0) | 3.9 (3.7-4.1) | 3.9 (3.7-4.1) | 0.914 | 3.9 (3.8-4.1) | 3.9 (3.7-4.1) | 3.9 (3.7-4.0) | 3.8 (3.7-4.1) | 0.166 |
| BP1_4E | 8.0 (7.4-8.5) | 7.9 (7.4-8.5) | 7.9 (7.4-8.6) | 8.0 (7.4-8.5) | 0.725 | 7.8 (7.3-8.3) | 8.0 (7.6-8.5) | 7.9 (7.4-8.7) | 8.1 (7.5-8.6) | 0.029 |
| CA5A | 1.9 (1.4-2.7) | 1.9 (1.3-2.4) | 1.8 (1.3-2.2) | 1.8 (1.2-2.2) | 0.010 | 1.8 (1.3-2.4) | 1.8 (1.3-2.4) | 1.9 (1.4-2.4) | 1.8 (1.3-2.4) | 0.920 |
| CASP3 | 6.0 (4.9-7.0) | 6.4 (5.3-7.3) | 6.1 (5.2-7.2) | 5.7 (4.9-7.1) | 0.474 | 5.8 (4.8-7.0) | 6.3 (5.3-7.1) | 5.8 (4.8-7.2) | 6.0 (5.1-7.3) | 0.268 |
| CASP8 | 2.1 (1.9-2.4) | 2.1 (1.9-2.5) | 2.1 (1.8-2.4) | 2.1 (1.8-2.4) | 0.236 | 2.1 (1.8-2.4) | 2.1 (1.8-2.4) | 2.1 (1.8-2.4) | 2.1 (1.8-2.5) | 0.518 |
| CCL11 | 7.0 (6.7-7.3) | 7.0 (6.7-7.3) | 7.0 (6.7-7.3) | 6.9 (6.7-7.2) | 0.214 | 7.0 (6.7-7.3) | 7.0 (6.7-7.3) | 6.9 (6.7-7.3) | 7.0 (6.7-7.3) | 0.581 |
| CCL15 | 6.7 (6.5-7.1) | 6.7 (6.5-7.0) | 6.7 (6.5-7.1) | 6.6 (6.4-7.0) | 0.156 | 6.7 (6.5-7.1) | 6.7 (6.4-7.0) | 6.7 (6.4-7.0) | 6.8 (6.5-7.1) | 0.627 |
| CCL16 | 6.4 (6.0-6.7) | 6.4 (6.0-6.7) | 6.4 (6.0-6.6) | 6.3 (5.9-6.7) | 0.165 | 6.3 (6.0-6.6) | 6.4 (6.0-6.8) | 6.3 (5.9-6.6) | 6.5 (6.1-6.8) | 0.501 |
| CCL17 | 6.9 (6.4-7.6) | 7.0 (6.5-7.8) | 6.9 (6.4-7.7) | 6.9 (6.3-7.6) | 0.722 | 6.9 (6.2-7.6) | 7.1 (6.6-7.8) | 6.7 (6.2-7.5) | 6.9 (6.5-7.8) | 0.829 |
| CCL19 | 9.3 (8.8-9.9) | 9.2 (8.6-9.8) | 9.0 (8.7-9.6) | 9.0 (8.7-9.7) | 0.017 | 9.2 (8.7-9.9) | 9.1 (8.7-9.6) | 9.1 (8.7-9.6) | 9.1 (8.7-9.7) | 0.501 |
| CCL20 | 6.1 (5.6-6.8) | 6.2 (5.6-7.0) | 6.1 (5.6-6.6) | 6.1 (5.6-6.7) | 0.451 | 6.1 (5.5-6.9) | 6.1 (5.7-6.8) | 6.2 (5.5-6.7) | 6.1 (5.5-6.7) | 0.514 |
| CCL23 | 9.1 (8.7-9.4) | 9.0 (8.8-9.2) | 9.1 (8.8-9.4) | 9.0 (8.7-9.4) | 0.817 | 9.0 (8.7-9.3) | 9.1 (8.8-9.4) | 9.0 (8.8-9.3) | 9.1 (8.7-9.4) | 0.798 |
| CCL24 | 5.0 (4.4-5.6) | 5.0 (4.3-5.6) | 4.9 (4.2-5.7) | 4.9 (4.2-5.6) | 0.580 | 5.0 (4.4-5.7) | 4.9 (4.3-5.4) | 4.9 (4.2-5.6) | 4.9 (4.3-5.5) | 0.37 |
| CCL25 | 6.4 (5.9-6.7) | 6.5 (6.0-6.9) | 6.3 (5.9-6.6) | 6.4 (5.9-6.8) | 0.356 | 6.3 (6.0-6.7) | 6.4 (6.0-6.8) | 6.4 (5.9-6.8) | 6.4 (6.0-6.6) | 0.996 |
| CCL28 | 2.6 (2.4-2.8) | 2.5 (2.3-2.7) | 2.5 (2.3-2.7) | 2.5 (2.3-2.7) | 0.107 | 2.6 (2.3-2.7) | 2.5 (2.3-2.8) | 2.5 (2.4-2.8) | 2.5 (2.3-2.7) | 0.491 |
| CCL3 | 5.6 (5.3-5.9) | 5.6 (5.2-5.9) | 5.5 (5.2-6.0) | 5.6 (5.3-6.0) | 0.971 | 5.6 (5.2-6.0) | 5.5 (5.1-5.9) | 5.7 (5.3-5.9) | 5.5 (5.2-6.0) | 0.862 |
| CCL4 | 5.7 (5.3-5.9) | 5.5 (5.2-6.0) | 5.5 (5.2-5.8) | 5.6 (5.2-6.0) | 0.559 | 5.6 (5.3-5.9) | 5.5 (5.2-5.9) | 5.6 (5.3-6.0) | 5.5 (5.2-5.9) | 0.851 |
| CD_40 | 11.0 (10.7-11.4) | 11.0 (10.7-11.3) | 11.0 (10.7-11.3) | 10.9 (10.7-11.3) | 0.564 | 11.0 (10.6-11.2) | 11.0 (10.8-11.3) | 10.9 (10.7-11.3) | 11.0 (10.7-11.3) | 0.386 |
| CD163 | 7.9 (7.7-8.2) | 7.7 (7.4-8.0) | 7.7 (7.5-8.1) | 7.7 (7.5-8.0) | 0.013 | 7.9 (7.6-8.1) | 7.7 (7.5-8.1) | 7.8 (7.4-8.1) | 7.7 (7.5-8.1) | 0.556 |
| CD244 | 6.7 (6.4-7.0) | 6.6 (6.4-6.9) | 6.6 (6.4-6.9) | 6.6 (6.4-6.9) | 0.097 | 6.6 (6.3-6.9) | 6.7 (6.4-7.0) | 6.6 (6.4-6.9) | 6.7 (6.4-6.9) | 0.717 |
| CD4 | 4.2 (4.1-4.4) | 4.2 (4.0-4.4) | 4.2 (4.1-4.4) | 4.2 (4.0-4.4) | 0.089 | 4.2 (4.0-4.4) | 4.2 (4.1-4.4) | 4.2 (4.0-4.3) | 4.2 (4.0-4.5) | 0.788 |
| CD40L | 4.2 (3.6-5.3) | 4.6 (3.9-5.9) | 4.6 (3.8-5.6) | 4.1 (3.4-5.5) | 0.61 | 4.2 (3.6-5.2) | 4.7 (3.8-5.8) | 4.2 (3.4-5.7) | 4.3 (3.8-5.6) | 0.614 |
| CD5 | 4.5 (4.2-4.8) | 4.5 (4.2-4.7) | 4.5 (4.2-4.7) | 4.5 (4.2-4.7) | 0.107 | 4.5 (4.2-4.7) | 4.5 (4.2-4.8) | 4.5 (4.2-4.8) | 4.5 (4.2-4.8) | 0.809 |
| CD6 | 5.6 (5.2-5.8) | 5.5 (5.2-5.7) | 5.4 (5.1-5.7) | 5.4 (5.1-5.8) | 0.164 | 5.5 (5.2-5.7) | 5.4 (5.2-5.8) | 5.5 (5.2-5.8) | 5.4 (5.2-5.7) | 0.469 |
| CD84 | 4.5 (4.2-4.9) | 4.6 (4.3-5.0) | 4.5 (4.3-4.9) | 4.5 (4.1-4.8) | 0.339 | 4.5 (4.2-4.9) | 4.6 (4.3-5.0) | 4.5 (4.2-4.8) | 4.5 (4.2-4.8) | 0.51 |
| CD8A | 9.5 (9.1-10.0) | 9.4 (8.8-9.9) | 9.5 (9.1-10.2) | 9.4 (9.0-9.9) | 0.654 | 9.5 (9.0-10.1) | 9.3 (8.9-10.0) | 9.4 (9.1-10.0) | 9.5 (9.1-9.9) | 0.72 |
| CD93 | 10.6 (10.3-10.8) | 10.6 (10.4-10.8) | 10.5 (10.3-10.7) | 10.5 (10.3-10.7) | 0.109 | 10.5 (10.3-10.7) | 10.6 (10.3-10.7) | 10.5 (10.3-10.7) | 10.6 (10.4-10.8) | 0.289 |
| CDCP1 | 4.4 (4.0-4.8) | 4.4 (4.1-4.8) | 4.3 (3.9-4.9) | 4.4 (4.0-4.8) | 0.583 | 4.4 (4.0-4.8) | 4.4 (3.9-4.9) | 4.4 (4.1-4.9) | 4.4 (3.9-4.9) | 0.582 |
| CDH5 | 4.1 (3.9-4.4) | 4.1 (3.9-4.3) | 4.1 (3.8-4.3) | 4.0 (3.7-4.3) | 0.174 | 4.1 (3.9-4.3) | 4.0 (3.8-4.4) | 4.1 (3.8-4.3) | 4.0 (3.8-4.3) | 0.862 |
| CEACAM8 | 3.9 (3.6-4.4) | 3.9 (3.5-4.3) | 3.9 (3.6-4.2) | 3.9 (3.5-4.4) | 0.977 | 3.9 (3.6-4.4) | 3.9 (3.5-4.2) | 3.9 (3.6-4.1) | 4.0 (3.6-4.4) | 0.496 |
| CHI3L1 | 4.5 (3.7-5.1) | 4.3 (3.8-5.2) | 4.2 (3.5-5.0) | 4.2 (3.7-4.9) | 0.146 | 4.4 (3.8-5.0) | 4.2 (3.6-5.1) | 4.2 (3.6-5.0) | 4.4 (3.9-5.3) | 0.183 |
| CHIT1 | 5.3 (4.7-6.0) | 5.3 (4.8-6.1) | 5.2 (4.6-5.8) | 5.3 (4.6-5.9) | 0.393 | 5.5 (4.7-6.1) | 5.2 (4.7-5.8) | 5.2 (4.6-6.0) | 5.3 (4.7-6.1) | 0.801 |
| CNTN1 | 4.6 (4.4-4.8) | 4.5 (4.4-4.8) | 4.5 (4.3-4.8) | 4.5 (4.3-4.8) | 0.115 | 4.6 (4.4-4.8) | 4.5 (4.3-4.8) | 4.6 (4.3-4.8) | 4.5 (4.3-4.8) | 0.609 |
| COL1A1 | 2.9 (2.6-3.1) | 2.8 (2.6-3.0) | 2.8 (2.5-3.0) | 2.7 (2.4-3.0) | 0.002 | 3.0 (2.6-3.2) | 2.8 (2.6-3.0) | 2.8 (2.4-3.0) | 2.7 (2.4-2.9) | <0.001 |
| CPA1 | 5.7 (5.2-6.2) | 5.6 (5.1-6.2) | 5.6 (5.3-6.1) | 5.7 (5.2-6.2) | 0.764 | 5.7 (5.0-6.2) | 5.6 (5.3-6.1) | 5.7 (5.3-6.2) | 5.7 (5.1-6.2) | 0.612 |
| CPB1 | 5.3 (4.9-5.8) | 5.3 (4.7-5.8) | 5.3 (5.0-5.8) | 5.3 (4.8-5.8) | 0.591 | 5.3 (4.8-5.9) | 5.3 (4.9-5.7) | 5.4 (4.9-5.8) | 5.3 (4.8-5.8) | 0.929 |
| CSF1 | 9.9 (9.7-10.1) | 9.8 (9.7-10.0) | 9.8 (9.7-10.0) | 9.8 (9.7-10.0) | 0.172 | 9.9 (9.7-10.1) | 9.8 (9.7-10.0) | 9.8 (9.7-10.0) | 9.9 (9.7-10.1) | 0.909 |
| CST5 | 6.0 (5.8-6.3) | 6.0 (5.6-6.4) | 6.1 (5.6-6.5) | 6.0 (5.6-6.3) | 0.398 | 6.1 (5.8-6.4) | 6.0 (5.8-6.4) | 5.9 (5.6-6.4) | 6.0 (5.7-6.4) | 0.632 |
| CSTB | 4.2 (3.9-4.5) | 4.2 (3.8-4.6) | 4.1 (3.8-4.6) | 4.1 (3.7-4.4) | 0.252 | 4.1 (3.7-4.5) | 4.1 (3.8-4.5) | 4.1 (3.8-4.6) | 4.2 (3.9-4.6) | 0.075 |
| CTRC | 9.7 (9.1-10.2) | 9.6 (9.2-10.1) | 9.7 (9.2-10.2) | 9.8 (9.3-10.1) | 0.298 | 9.7 (9.1-10.2) | 9.6 (9.2-10.2) | 9.8 (9.3-10.1) | 9.7 (9.1-10.1) | 0.996 |
| CTSD | 2.2 (2.0-2.6) | 2.3 (2.0-2.7) | 2.2 (2.0-2.4) | 2.2 (1.9-2.5) | 0.119 | 2.2 (1.9-2.6) | 2.2 (2.0-2.5) | 2.3 (2.0-2.5) | 2.2 (1.9-2.5) | 0.393 |
| CTSL1 | 6.4 (6.2-6.7) | 6.4 (6.2-6.6) | 6.4 (6.2-6.6) | 6.4 (6.2-6.7) | 0.566 | 6.4 (6.2-6.7) | 6.3 (6.2-6.6) | 6.4 (6.2-6.6) | 6.4 (6.2-6.7) | 0.077 |
| CTSZ | 5.3 (5.0-5.5) | 5.2 (4.9-5.5) | 5.2 (4.9-5.6) | 5.2 (4.9-5.4) | 0.096 | 5.2 (4.9-5.5) | 5.2 (4.9-5.5) | 5.2 (4.9-5.5) | 5.2 (5.0-5.6) | 0.368 |
| CX3CL1 | 6.6 (6.3-6.9) | 6.4 (6.2-6.8) | 6.5 (6.2-6.8) | 6.5 (6.3-6.9) | 0.159 | 6.5 (6.2-6.8) | 6.5 (6.2-6.9) | 6.6 (6.3-6.9) | 6.6 (6.2-6.9) | 0.538 |
| CXCL_10 | 10.4 (10.0-11.0) | 10.3 (9.9-10.8) | 10.2 (9.8-10.7) | 10.3 (10.0-10.7) | 0.273 | 10.4 (10.1-10.9) | 10.3 (9.8-10.9) | 10.3 (10.0-10.9) | 10.3 (9.9-10.7) | 0.281 |
| CXCL_16 | 5.0 (4.8-5.2) | 5.0 (4.8-5.2) | 5.0 (4.8-5.2) | 4.9 (4.7-5.1) | 0.045 | 4.9 (4.8-5.2) | 4.9 (4.8-5.2) | 5.0 (4.7-5.1) | 5.0 (4.8-5.2) | 0.29 |
| CXCL1 | 8.2 (7.5-9.1) | 8.9 (7.6-9.6) | 8.7 (7.7-9.5) | 7.9 (7.4-9.3) | 0.663 | 8.3 (7.5-9.1) | 8.6 (7.8-9.6) | 8.3 (7.4-9.5) | 8.6 (7.4-9.5) | 0.612 |
| CXCL11 | 7.1 (6.4-7.8) | 7.2 (6.8-7.7) | 7.1 (6.6-7.5) | 7.1 (6.6-7.6) | 0.522 | 7.0 (6.4-7.6) | 7.2 (6.7-7.7) | 7.1 (6.6-7.7) | 7.2 (6.7-7.6) | 0.241 |
| CXCL5 | 8.9 (8.0-10.3) | 9.9 (8.4-10.8) | 9.2 (7.8-10.3) | 8.6 (7.5-10.2) | 0.12 | 9.1 (7.9-9.9) | 9.5 (8.4-10.6) | 9.2 (7.5-10.4) | 8.7 (7.7-10.3) | 0.607 |
| CXCL6 | 7.2 (6.8-8.1) | 7.4 (6.9-7.9) | 7.3 (6.8-7.8) | 7.2 (6.8-7.8) | 0.449 | 7.1 (6.7-7.8) | 7.3 (6.9-8.0) | 7.3 (6.8-7.9) | 7.2 (6.8-7.8) | 0.593 |
| CXCL9 | 7.8 (7.4-8.4) | 7.8 (7.2-8.2) | 7.7 (7.3-8.2) | 7.7 (7.4-8.3) | 0.639 | 7.8 (7.3-8.4) | 7.8 (7.2-8.4) | 7.7 (7.3-8.2) | 7.7 (7.4-8.3) | 0.919 |
| DCN | 4.4 (4.2-4.7) | 4.4 (4.1-4.7) | 4.4 (4.2-4.6) | 4.3 (4.1-4.5) | 0.035 | 4.4 (4.2-4.6) | 4.3 (4.1-4.6) | 4.4 (4.2-4.6) | 4.3 (4.1-4.6) | 0.291 |
| DECR1 | 5.6 (4.7-6.7) | 6.0 (4.9-7.2) | 5.9 (4.8-6.9) | 5.5 (4.4-6.6) | 0.339 | 5.6 (4.5-6.5) | 5.8 (4.9-7.0) | 5.6 (4.6-6.8) | 5.8 (4.6-6.8) | 0.511 |
| DKK1 | 7.2 (6.8-7.7) | 7.4 (7.0-7.8) | 7.3 (7.0-7.7) | 7.2 (6.7-7.6) | 0.955 | 7.2 (6.8-7.7) | 7.2 (7.0-7.8) | 7.2 (6.9-7.6) | 7.3 (6.8-7.7) | 0.376 |
| DLK1 | 6.0 (5.6-6.4) | 6.0 (5.7-6.4) | 6.1 (5.6-6.4) | 5.9 (5.4-6.3) | 0.114 | 6.1 (5.5-6.5) | 6.0 (5.6-6.3) | 6.0 (5.6-6.4) | 6.0 (5.5-6.4) | 0.878 |
| DNER | 8.5 (8.3-8.7) | 8.5 (8.3-8.7) | 8.5 (8.3-8.7) | 8.6 (8.4-8.8) | 0.179 | 8.5 (8.3-8.7) | 8.5 (8.3-8.8) | 8.6 (8.3-8.7) | 8.5 (8.3-8.8) | 0.325 |
| EGFR | 2.7 (2.5-2.8) | 2.7 (2.5-2.8) | 2.7 (2.5-2.8) | 2.7 (2.5-2.8) | 0.958 | 2.7 (2.5-2.8) | 2.7 (2.5-2.9) | 2.7 (2.5-2.8) | 2.7 (2.5-2.9) | 0.833 |
| ENRAGE | 2.5 (2.1-3.1) | 2.5 (2.1-3.0) | 2.5 (2.1-3.0) | 2.5 (2.1-3.0) | 0.844 | 2.5 (2.1-3.0) | 2.5 (2.1-2.9) | 2.5 (2.1-3.2) | 2.6 (2.2-3.1) | 0.121 |
| EPCAM | 5.1 (4.3-5.7) | 4.9 (4.3-5.9) | 5.1 (4.5-5.6) | 5.0 (4.5-5.7) | 0.601 | 5.2 (4.4-5.8) | 5.0 (4.4-5.7) | 5.0 (4.4-5.7) | 5.0 (4.5-5.7) | 0.602 |
| EPHB4 | 5.4 (5.2-5.7) | 5.4 (5.2-5.7) | 5.4 (5.2-5.6) | 5.4 (5.1-5.6) | 0.077 | 5.4 (5.2-5.7) | 5.4 (5.2-5.6) | 5.4 (5.2-5.7) | 5.4 (5.2-5.7) | 0.419 |
| FABP2 | 8.4 (7.9-9.0) | 8.3 (7.9-8.9) | 8.6 (8.1-8.9) | 8.4 (7.7-9.0) | 0.799 | 8.4 (7.9-9.0) | 8.4 (8.0-8.9) | 8.4 (7.9-8.9) | 8.4 (7.8-8.8) | 0.389 |
| FABP4 | 5.9 (5.4-6.4) | 5.8 (5.3-6.1) | 5.8 (5.2-6.1) | 5.6 (5.2-6.2) | 0.086 | 5.7 (5.2-6.4) | 5.8 (5.3-6.3) | 5.7 (5.1-6.1) | 5.8 (5.5-6.2) | 0.759 |
| FAS | 5.9 (5.7-6.1) | 5.9 (5.7-6.1) | 5.8 (5.6-6.1) | 5.8 (5.6-6.0) | 0.051 | 5.8 (5.7-6.1) | 5.8 (5.6-6.0) | 5.8 (5.6-6.0) | 5.9 (5.7-6.1) | 0.528 |
| FCGR2B | 3.5 (3.0-4.1) | 3.3 (2.7-3.8) | 3.4 (2.7-4.1) | 3.5 (2.8-4.0) | 0.892 | 3.3 (2.7-4.1) | 3.5 (2.8-4.0) | 3.5 (2.8-4.0) | 3.4 (2.8-3.9) | 0.822 |
| FGF19 | 7.7 (7.2-8.2) | 7.5 (6.9-8.1) | 7.4 (6.8-8.0) | 7.5 (6.9-7.9) | 0.023 | 7.6 (7.0-8.2) | 7.5 (6.8-8.0) | 7.4 (6.9-8.1) | 7.5 (7.0-8.1) | 0.983 |
| FGF21 | 5.5 (4.8-6.0) | 5.4 (4.8-6.4) | 5.3 (4.6-6.2) | 5.4 (4.5-6.1) | 0.299 | 5.5 (4.8-6.1) | 5.5 (4.7-6.1) | 5.4 (4.6-6.2) | 5.4 (4.8-6.2) | 0.761 |
| FGF23 | 3.3 (3.1-3.7) | 3.3 (3.0-3.6) | 3.3 (3.0-3.6) | 3.3 (3.0-3.6) | 0.112 | 3.3 (3.0-3.6) | 3.3 (3.1-3.6) | 3.3 (3.0-3.6) | 3.3 (3.0-3.7) | 0.874 |
| FGF5 | 0.5 (0.4-0.6) | 0.5 (0.4-0.6) | 0.4 (0.4-0.5) | 0.5 (0.4-0.6) | 0.008 | 0.5 (0.4-0.6) | 0.5 (0.4-0.6) | 0.4 (0.4-0.6) | 0.5 (0.4-0.6) | 0.195 |
| FLT3L | 9.5 (9.2-9.8) | 9.4 (9.1-9.6) | 9.4 (9.1-9.6) | 9.4 (9.1-9.6) | 0.029 | 9.4 (9.2-9.7) | 9.4 (9.2-9.7) | 9.4 (9.2-9.6) | 9.4 (9.1-9.6) | 0.554 |
| FS | 10.8 (10.6-11.1) | 10.8 (10.5-11.1) | 10.8 (10.4-11.0) | 10.8 (10.4-11.0) | 0.294 | 10.8 (10.6-11.0) | 10.8 (10.5-11.2) | 10.8 (10.5-11.0) | 10.8 (10.4-11.1) | 0.921 |
| GAL4 | 4.0 (3.6-4.5) | 4.0 (3.6-4.5) | 4.0 (3.6-4.3) | 3.9 (3.5-4.2) | 0.020 | 4.0 (3.6-4.4) | 4.0 (3.7-4.4) | 3.9 (3.5-4.3) | 3.9 (3.5-4.3) | 0.130 |
| GAL9 | 7.4 (7.2-7.6) | 7.3 (7.1-7.6) | 7.4 (7.2-7.6) | 7.3 (7.2-7.6) | 0.851 | 7.4 (7.2-7.6) | 7.4 (7.2-7.6) | 7.4 (7.2-7.6) | 7.4 (7.2-7.6) | 0.874 |
| GDF2 | 7.4 (7.2-7.6) | 7.4 (7.1-7.6) | 7.4 (7.1-7.6) | 7.3 (7.1-7.6) | 0.150 | 7.4 (7.1-7.6) | 7.4 (7.1-7.7) | 7.4 (7.0-7.6) | 7.3 (7.1-7.6) | 0.344 |
| GDNF | 2.2 (2.0-2.5) | 2.2 (1.9-2.4) | 2.1 (1.9-2.4) | 2.1 (1.9-2.4) | 0.091 | 2.1 (1.9-2.4) | 2.2 (1.9-2.5) | 2.1 (1.9-2.4) | 2.2 (1.9-2.4) | 0.759 |
| GH | 7.4 (6.4-9.0) | 7.2 (5.7-8.3) | 6.9 (5.5-8.7) | 7.5 (6.2-9.2) | 0.921 | 7.6 (6.4-8.9) | 7.2 (5.7-8.4) | 7.1 (5.7-8.7) | 7.1 (6.0-8.9) | 0.209 |
| GIF | 8.4 (7.8-9.2) | 8.4 (7.7-8.9) | 8.5 (7.9-9.4) | 8.3 (7.9-9.2) | 0.920 | 8.5 (7.6-9.4) | 8.5 (7.9-9.2) | 8.4 (7.8-9.1) | 8.3 (7.8-9.0) | 0.261 |
| GLO1 | 5.7 (5.2-6.3) | 5.7 (5.2-6.3) | 5.7 (5.4-6.4) | 5.7 (5.2-6.2) | 0.976 | 5.6 (5.1-6.1) | 5.8 (5.3-6.4) | 5.7 (5.2-6.1) | 5.8 (5.4-6.3) | 0.183 |
| GP6 | 2.1 (1.6-2.7) | 2.3 (1.8-2.9) | 2.1 (1.7-2.8) | 1.9 (1.6-2.6) | 0.347 | 2.1 (1.5-2.6) | 2.1 (1.7-2.8) | 2.0 (1.6-2.7) | 2.2 (1.7-2.8) | 0.377 |
| GRN | 5.1 (4.9-5.4) | 5.2 (4.9-5.3) | 5.1 (4.9-5.4) | 5.1 (4.9-5.3) | 0.224 | 5.1 (4.9-5.4) | 5.2 (4.9-5.4) | 5.1 (4.9-5.3) | 5.1 (4.9-5.3) | 0.619 |
| GT | 1.8 (1.3-2.3) | 1.8 (1.4-2.2) | 1.8 (1.4-2.2) | 1.7 (1.2-2.3) | 0.892 | 1.8 (1.5-2.3) | 1.8 (1.4-2.2) | 1.7 (1.3-2.3) | 1.7 (1.3-2.2) | 0.184 |
| HAOX1 | 4.6 (3.9-5.6) | 4.8 (3.9-5.7) | 4.5 (3.9-5.2) | 4.4 (3.8-5.4) | 0.309 | 4.4 (3.7-5.3) | 4.8 (4.0-5.7) | 4.4 (3.8-5.5) | 4.5 (4.0-5.5) | 0.548 |
| HBEGF | 4.3 (3.9-4.7) | 4.4 (4.1-4.9) | 4.4 (4.1-4.8) | 4.3 (4.0-4.7) | 0.650 | 4.3 (4.0-4.7) | 4.4 (4.1-5.0) | 4.3 (3.9-4.7) | 4.4 (4.1-4.8) | 0.356 |
| HGF | 8.6 (8.3-8.8) | 8.5 (8.3-8.8) | 8.5 (8.2-8.7) | 8.5 (8.2-8.8) | 0.038 | 8.6 (8.3-8.8) | 8.6 (8.3-8.8) | 8.5 (8.2-8.7) | 8.6 (8.2-8.9) | 0.555 |
| HO1 | 11.2 (10.9-11.5) | 11.2 (11.0-11.5) | 11.3 (11.0-11.5) | 11.3 (11.0-11.6) | 0.064 | 11.2 (11.0-11.5) | 11.2 (10.9-11.5) | 11.3 (11.0-11.5) | 11.3 (11.1-11.6) | 0.006 |
| HOSCAR | 10.9 (10.7-11.0) | 10.8 (10.6-11.0) | 10.8 (10.6-11.0) | 10.8 (10.6-11.0) | 0.399 | 10.8 (10.7-11.0) | 10.8 (10.6-11.0) | 10.8 (10.6-10.9) | 10.8 (10.6-11.0) | 0.482 |
| HSP27 | 8.7 (8.4-8.9) | 8.8 (8.4-9.0) | 8.8 (8.4-9.0) | 8.7 (8.3-9.0) | 0.936 | 8.7 (8.3-8.9) | 8.8 (8.4-9.0) | 8.7 (8.4-9.0) | 8.8 (8.4-9.0) | 0.223 |
| ICAM2 | 5.0 (4.8-5.2) | 5.0 (4.8-5.2) | 4.9 (4.6-5.3) | 5.0 (4.6-5.2) | 0.408 | 5.0 (4.7-5.2) | 5.0 (4.8-5.3) | 5.0 (4.7-5.3) | 4.9 (4.6-5.3) | 0.718 |
| IDUA | 5.5 (5.2-5.8) | 5.4 (5.1-5.8) | 5.4 (5.1-5.7) | 5.4 (5.1-5.6) | 0.039 | 5.4 (5.2-5.7) | 5.4 (5.1-5.7) | 5.4 (5.1-5.8) | 5.4 (5.0-5.6) | 0.497 |
| IGFBP1 | 5.2 (4.3-5.7) | 5.1 (4.3-5.9) | 5.0 (4.1-5.6) | 5.0 (4.5-5.6) | 0.436 | 5.1 (4.2-5.7) | 5.1 (4.4-5.9) | 5.1 (4.1-5.6) | 5.0 (4.4-5.7) | 0.864 |
| IGFBP2 | 7.6 (7.2-8.0) | 7.6 (7.2-7.9) | 7.5 (7.2-8.0) | 7.7 (7.3-8.1) | 0.431 | 7.6 (7.1-8.0) | 7.6 (7.3-8.0) | 7.6 (7.2-8.0) | 7.6 (7.3-8.2) | 0.120 |
| IGFBP7 | 7.7 (7.5-8.0) | 7.7 (7.5-7.9) | 7.7 (7.5-7.9) | 7.6 (7.4-7.8) | 0.002 | 7.7 (7.4-7.9) | 7.7 (7.5-7.9) | 7.6 (7.5-7.9) | 7.7 (7.5-8.0) | 0.462 |
| IL_20 | 0.5 (0.5-0.5) | 0.5 (0.5-0.5) | 0.5 (0.5-0.5) | 0.5 (0.5-0.5) | 0.339 | 0.5 (0.5-0.5) | 0.5 (0.5-0.5) | 0.5 (0.5-0.5) | 0.5 (0.5-0.5) | 0.195 |
| IL10 | 3.9 (3.6-4.2) | 3.8 (3.5-4.1) | 3.8 (3.6-4.1) | 3.8 (3.5-4.1) | 0.127 | 3.8 (3.6-4.1) | 3.8 (3.6-4.2) | 3.8 (3.5-4.1) | 3.8 (3.5-4.1) | 0.641 |
| IL10RA | 0.5 (0.4-0.8) | 0.5 (0.4-0.9) | 0.5 (0.4-0.9) | 0.5 (0.4-0.8) | 0.907 | 0.4 (0.4-0.7) | 0.5 (0.4-1.0) | 0.5 (0.4-0.9) | 0.5 (0.4-0.7) | 0.947 |
| IL10RB | 6.1 (5.9-6.3) | 6.1 (5.9-6.3) | 6.1 (5.9-6.3) | 6.0 (5.8-6.2) | 0.076 | 6.1 (5.9-6.3) | 6.1 (5.9-6.2) | 6.1 (5.8-6.3) | 6.1 (5.9-6.3) | 0.874 |
| IL12B | 6.3 (5.8-6.8) | 6.2 (5.8-6.7) | 6.2 (5.8-6.6) | 6.1 (5.7-6.5) | 0.044 | 6.3 (5.8-6.9) | 6.2 (5.8-6.7) | 6.2 (5.9-6.7) | 6.1 (5.7-6.5) | 0.014 |
| IL13 | 1.1 (1.1-1.1) | 1.1 (1.1-1.2) | 1.1 (1.1-1.1) | 1.1 (1.1-1.1) | 0.475 | 1.1 (1.1-1.1) | 1.1 (1.1-1.1) | 1.1 (1.1-1.1) | 1.1 (1.1-1.1) | 0.823 |
| IL15RA | 0.7 (0.5-1.0) | 0.7 (0.6-0.8) | 0.7 (0.5-0.9) | 0.7 (0.5-1.0) | 0.324 | 0.7 (0.5-0.9) | 0.7 (0.5-1.0) | 0.7 (0.6-0.9) | 0.7 (0.6-1.0) | 0.216 |
| IL16 | 5.9 (5.6-6.1) | 5.8 (5.6-6.2) | 6.0 (5.6-6.2) | 5.8 (5.5-6.1) | 0.615 | 5.9 (5.6-6.0) | 6.0 (5.6-6.2) | 5.8 (5.6-6.1) | 5.9 (5.6-6.3) | 0.607 |
| IL17A | 1.2 (0.7-1.7) | 1.1 (0.8-1.6) | 1.1 (0.7-1.5) | 1.1 (0.7-1.5) | 0.267 | 1.1 (0.7-1.5) | 1.3 (0.8-1.7) | 1.1 (0.8-1.6) | 1.1 (0.7-1.5) | 0.811 |
| IL17C | 1.7 (1.3-2.0) | 1.6 (1.2-2.1) | 1.7 (1.3-2.0) | 1.7 (1.3-2.1) | 0.771 | 1.6 (1.3-2.2) | 1.6 (1.3-2.3) | 1.6 (1.3-1.9) | 1.7 (1.3-2.0) | 0.719 |
| IL17D | 2.6 (2.5-2.9) | 2.6 (2.4-2.9) | 2.7 (2.4-2.8) | 2.6 (2.4-2.8) | 0.297 | 2.6 (2.4-2.8) | 2.7 (2.5-2.9) | 2.6 (2.4-2.8) | 2.6 (2.4-2.8) | 0.901 |
| IL17RA | 3.9 (3.5-4.3) | 3.8 (3.5-4.2) | 3.9 (3.6-4.2) | 3.8 (3.4-4.2) | 0.390 | 3.9 (3.5-4.2) | 3.9 (3.6-4.1) | 3.8 (3.5-4.2) | 3.9 (3.5-4.3) | 0.712 |
| IL18 | 8.3 (8.1-8.7) | 8.2 (7.9-8.5) | 8.2 (7.9-8.6) | 8.3 (7.9-8.6) | 0.495 | 8.3 (8.0-8.7) | 8.2 (7.8-8.5) | 8.3 (7.9-8.6) | 8.3 (7.9-8.7) | 0.641 |
| IL18BP | 5.9 (5.7-6.3) | 5.9 (5.6-6.1) | 5.9 (5.7-6.1) | 5.8 (5.6-6.1) | 0.064 | 5.9 (5.7-6.2) | 5.9 (5.6-6.1) | 5.9 (5.6-6.2) | 5.9 (5.7-6.2) | 0.862 |
| IL18R1 | 8.0 (7.7-8.2) | 7.8 (7.5-8.1) | 7.8 (7.5-8.1) | 7.9 (7.6-8.2) | 0.134 | 7.9 (7.7-8.2) | 7.9 (7.7-8.2) | 7.9 (7.6-8.2) | 7.9 (7.6-8.1) | 0.074 |
| IL1RA | 4.5 (4.2-4.9) | 4.5 (4.1-5.0) | 4.5 (4.3-4.8) | 4.5 (4.3-4.9) | 0.937 | 4.5 (4.3-5.1) | 4.5 (4.3-5.0) | 4.4 (4.1-4.8) | 4.5 (4.3-4.9) | 0.484 |
| IL1RL2 | 4.4 (4.1-4.6) | 4.3 (4.1-4.6) | 4.3 (4.0-4.6) | 4.3 (4.0-4.6) | 0.388 | 4.4 (4.1-4.6) | 4.3 (4.0-4.6) | 4.3 (4.1-4.6) | 4.3 (4.0-4.5) | 0.183 |
| IL1RT1 | 6.5 (6.3-6.7) | 6.4 (6.2-6.5) | 6.4 (6.2-6.7) | 6.4 (6.2-6.6) | 0.160 | 6.4 (6.2-6.6) | 6.4 (6.2-6.6) | 6.4 (6.2-6.6) | 6.4 (6.2-6.6) | 0.415 |
| IL1RT2 | 5.1 (5.0-5.4) | 5.1 (4.9-5.3) | 5.1 (4.9-5.4) | 5.1 (4.9-5.4) | 0.438 | 5.2 (5.0-5.4) | 5.1 (4.9-5.4) | 5.1 (4.9-5.3) | 5.1 (4.9-5.3) | 0.044 |
| IL20RA | 0.7 (0.7-0.8) | 0.7 (0.7-0.7) | 0.7 (0.7-0.7) | 0.7 (0.7-0.8) | 0.245 | 0.7 (0.7-0.7) | 0.7 (0.7-0.8) | 0.7 (0.7-0.8) | 0.7 (0.7-0.7) | 0.927 |
| IL22RA1 | 2.5 (2.5-2.5) | 2.5 (2.5-2.5) | 2.5 (2.5-2.5) | 2.5 (2.5-2.5) | 0.822 | 2.5 (2.5-2.5) | 2.5 (2.5-2.5) | 2.5 (2.5-2.5) | 2.5 (2.5-2.5) | 0.304 |
| IL24 | 2.0 (2.0-2.0) | 2.0 (2.0-2.0) | 2.0 (2.0-2.0) | 2.0 (2.0-2.0) | 0.967 | 2.0 (2.0-2.0) | 2.0 (2.0-2.0) | 2.0 (2.0-2.0) | 2.0 (2.0-2.0) | 0.873 |
| IL27 | 6.4 (6.1-6.6) | 6.3 (6.1-6.5) | 6.3 (6.2-6.6) | 6.4 (6.1-6.6) | 0.608 | 6.3 (6.1-6.6) | 6.3 (6.1-6.6) | 6.3 (6.1-6.6) | 6.4 (6.2-6.6) | 0.320 |
| IL2RA | 3.7 (3.4-4.1) | 3.6 (3.3-3.9) | 3.7 (3.4-3.9) | 3.6 (3.4-3.9) | 0.209 | 3.6 (3.4-4.0) | 3.6 (3.3-3.9) | 3.6 (3.3-3.9) | 3.7 (3.4-4.0) | 0.822 |
| IL2RB | 1.1 (1.1-1.1) | 1.1 (1.1-1.1) | 1.1 (1.1-1.1) | 1.1 (1.1-1.1) | 0.737 | 1.1 (1.1-1.1) | 1.1 (1.1-1.1) | 1.1 (1.1-1.1) | 1.1 (1.1-1.1) | 0.297 |
| IL4RA | 2.1 (2.0-2.4) | 2.1 (1.9-2.4) | 2.1 (1.9-2.3) | 2.1 (1.8-2.3) | 0.013 | 2.1 (1.9-2.4) | 2.1 (1.9-2.3) | 2.1 (1.9-2.3) | 2.1 (1.9-2.4) | 0.968 |
| IL5 | 1.3 (1.3-1.4) | 1.3 (1.3-1.3) | 1.3 (1.3-1.3) | 1.3 (1.3-1.4) | 0.850 | 1.3 (1.3-1.5) | 1.3 (1.3-1.5) | 1.3 (1.3-1.3) | 1.3 (1.3-1.3) | 0.136 |
| IL6 | 3.9 (3.4-4.5) | 3.8 (3.3-4.2) | 3.7 (3.2-4.2) | 3.8 (3.3-4.3) | 0.133 | 3.8 (3.4-4.3) | 3.8 (3.4-4.2) | 3.7 (3.2-4.2) | 3.7 (3.3-4.5) | 0.448 |
| IL6RA | 11.5 (11.3-11.8) | 11.6 (11.2-11.8) | 11.5 (11.3-11.8) | 11.6 (11.4-11.9) | 0.273 | 11.5 (11.3-11.8) | 11.6 (11.3-11.8) | 11.5 (11.3-11.7) | 11.6 (11.4-11.9) | 0.495 |
| IL7 | 1.9 (1.6-2.5) | 2.2 (1.7-2.6) | 2.0 (1.6-2.5) | 1.8 (1.5-2.4) | 0.108 | 1.8 (1.6-2.4) | 2.1 (1.7-2.7) | 2.0 (1.5-2.5) | 2.0 (1.5-2.5) | 0.885 |
| IL8 | 5.5 (5.1-5.9) | 5.5 (5.0-5.9) | 5.4 (5.0-5.8) | 5.5 (5.2-5.8) | 0.905 | 5.5 (5.0-5.7) | 5.5 (5.2-5.9) | 5.4 (4.9-5.8) | 5.5 (5.1-5.8) | 0.786 |
| ITGB1BP2 | 3.8 (3.0-4.9) | 4.3 (3.0-5.3) | 4.1 (3.1-5.2) | 3.7 (2.7-5.2) | 0.483 | 3.6 (2.7-4.7) | 4.3 (3.0-5.2) | 3.8 (2.8-5.3) | 3.8 (3.1-5.2) | 0.237 |
| ITGB2 | 5.1 (4.8-5.4) | 5.1 (4.8-5.4) | 5.0 (4.8-5.3) | 5.0 (4.8-5.3) | 0.050 | 5.1 (4.8-5.3) | 5.1 (4.8-5.4) | 5.1 (4.8-5.3) | 5.1 (4.8-5.4) | 0.802 |
| JAMA | 5.0 (4.3-5.8) | 5.2 (4.5-6.0) | 5.0 (4.5-6.0) | 4.8 (4.3-5.9) | 0.78 | 4.9 (4.2-5.7) | 5.2 (4.5-6.0) | 4.8 (4.3-5.9) | 4.9 (4.4-6.0) | 0.372 |
| KIM1 | 8.4 (7.9-8.9) | 8.2 (7.8-8.7) | 8.3 (7.8-8.9) | 8.1 (7.7-8.7) | 0.073 | 8.3 (7.8-8.7) | 8.2 (7.8-8.9) | 8.2 (7.8-8.8) | 8.2 (7.7-8.9) | 0.93 |
| KLK6 | 2.3 (2.0-2.6) | 2.3 (2.0-2.7) | 2.3 (2.1-2.6) | 2.3 (1.9-2.5) | 0.288 | 2.3 (2.0-2.6) | 2.3 (2.1-2.6) | 2.3 (2.0-2.6) | 2.3 (2.0-2.7) | 0.785 |
| LAPTGFBE1 | 6.8 (6.5-7.1) | 6.7 (6.5-7.1) | 6.7 (6.4-6.9) | 6.6 (6.4-6.9) | 0.008 | 6.7 (6.5-6.9) | 6.7 (6.5-7.1) | 6.7 (6.4-7.0) | 6.7 (6.4-7.0) | 0.801 |
| LDLRECEP | 4.2 (3.9-4.5) | 4.2 (3.9-4.5) | 4.3 (4.0-4.6) | 4.1 (3.8-4.4) | 0.312 | 4.2 (3.9-4.4) | 4.2 (4.0-4.5) | 4.2 (3.9-4.5) | 4.2 (3.8-4.4) | 0.494 |
| LEP | 6.6 (5.9-7.1) | 6.4 (5.6-6.9) | 6.3 (5.8-6.9) | 6.2 (5.3-6.9) | 0.004 | 6.5 (5.7-6.9) | 6.5 (5.6-7.0) | 6.2 (5.5-6.9) | 6.4 (5.8-7.0) | 0.644 |
| LIFR | 3.7 (3.5-3.9) | 3.6 (3.5-3.9) | 3.6 (3.4-3.8) | 3.6 (3.5-3.8) | 0.045 | 3.6 (3.5-3.8) | 3.7 (3.5-3.9) | 3.7 (3.5-3.9) | 3.6 (3.4-3.8) | 0.321 |
| LOX1 | 5.9 (5.6-6.2) | 5.8 (5.5-6.2) | 5.8 (5.5-6.2) | 5.8 (5.4-6.2) | 0.516 | 5.8 (5.5-6.2) | 5.9 (5.5-6.2) | 5.8 (5.4-6.0) | 5.8 (5.5-6.2) | 0.615 |
| LPL | 9.4 (9.1-9.7) | 9.3 (9.0-9.7) | 9.4 (9.1-9.7) | 9.5 (9.1-9.9) | 0.295 | 9.4 (9.1-9.7) | 9.4 (9.1-9.8) | 9.4 (8.9-9.7) | 9.5 (9.1-9.8) | 0.452 |
| LTBR | 3.8 (3.6-4.0) | 3.8 (3.6-4.0) | 3.8 (3.5-4.1) | 3.7 (3.5-3.9) | 0.066 | 3.7 (3.5-4.1) | 3.8 (3.5-4.0) | 3.8 (3.5-4.0) | 3.8 (3.6-4.1) | 0.362 |
| MARCO | 6.5 (6.3-6.6) | 6.4 (6.3-6.5) | 6.5 (6.3-6.6) | 6.5 (6.3-6.6) | 0.979 | 6.4 (6.3-6.6) | 6.5 (6.3-6.6) | 6.4 (6.3-6.6) | 6.5 (6.3-6.7) | 0.330 |
| MB | 8.1 (7.7-8.5) | 8.1 (7.8-8.6) | 8.2 (7.8-8.5) | 8.1 (7.7-8.4) | 0.778 | 8.1 (7.6-8.5) | 8.1 (7.8-8.4) | 8.2 (7.8-8.6) | 8.2 (7.8-8.7) | 0.021 |
| MCP1 | 10.5 (10.2-10.7) | 10.4 (10.2-10.7) | 10.4 (10.2-10.7) | 10.4 (10.2-10.7) | 0.620 | 10.4 (10.2-10.7) | 10.5 (10.3-10.7) | 10.4 (10.2-10.6) | 10.4 (10.2-10.7) | 0.256 |
| MCP2 | 9.3 (8.9-9.6) | 9.2 (8.7-9.6) | 9.2 (8.9-9.5) | 9.2 (8.9-9.5) | 0.179 | 9.2 (8.9-9.6) | 9.2 (8.9-9.7) | 9.2 (8.8-9.5) | 9.2 (8.8-9.5) | 0.534 |
| MCP3 | 1.4 (1.1-1.9) | 1.4 (1.1-1.8) | 1.4 (1.1-1.8) | 1.4 (1.1-1.8) | 0.813 | 1.4 (1.0-1.9) | 1.4 (1.1-1.8) | 1.5 (1.1-1.8) | 1.4 (1.2-1.8) | 0.180 |
| MCP4 | 12.9 (12.4-13.4) | 13.0 (12.6-13.4) | 12.9 (12.5-13.3) | 12.8 (12.5-13.3) | 0.435 | 12.8 (12.4-13.3) | 12.9 (12.6-13.4) | 12.9 (12.5-13.3) | 12.9 (12.6-13.3) | 0.481 |
| MEPE | 5.7 (5.4-6.0) | 5.7 (5.4-6.1) | 5.7 (5.4-6.0) | 5.6 (5.3-6.0) | 0.375 | 5.7 (5.4-6.0) | 5.6 (5.4-5.9) | 5.7 (5.4-5.9) | 5.7 (5.4-6.1) | 0.526 |
| MERTK | 5.9 (5.7-6.1) | 5.9 (5.6-6.2) | 5.9 (5.7-6.1) | 5.8 (5.6-6.2) | 0.730 | 5.9 (5.7-6.2) | 5.9 (5.6-6.1) | 5.9 (5.7-6.1) | 5.9 (5.6-6.2) | 0.629 |
| MMP10 | 6.7 (6.3-7.1) | 6.7 (6.3-7.0) | 6.7 (6.3-7.1) | 6.6 (6.4-7.1) | 0.742 | 6.7 (6.3-7.1) | 6.6 (6.4-7.0) | 6.7 (6.3-7.0) | 6.7 (6.3-7.1) | 0.640 |
| MMP1 | 9.2 (8.5-10.1) | 9.2 (8.7-10.0) | 9.3 (8.7-9.9) | 9.0 (8.4-10.0) | 0.510 | 9.2 (8.6-10.1) | 9.4 (8.6-10.1) | 9.1 (8.4-9.9) | 9.1 (8.5-9.9) | 0.213 |
| MMP12 | 7.4 (6.9-7.8) | 7.3 (6.8-7.9) | 7.4 (7.0-7.9) | 7.2 (6.7-7.8) | 0.527 | 7.3 (6.7-7.9) | 7.4 (6.9-7.8) | 7.3 (6.9-7.7) | 7.3 (6.8-7.9) | 0.917 |
| MMP2 | 3.9 (3.6-4.1) | 3.8 (3.6-4.0) | 3.8 (3.6-4.0) | 3.8 (3.6-4.0) | 0.050 | 3.8 (3.6-4.1) | 3.8 (3.6-4.0) | 3.8 (3.6-4.0) | 3.8 (3.6-4.0) | 0.797 |
| MMP3 | 7.9 (7.3-8.3) | 7.7 (7.3-8.1) | 7.8 (7.4-8.2) | 7.8 (7.4-8.2) | 0.843 | 7.7 (7.3-8.2) | 7.7 (7.2-8.0) | 7.8 (7.4-8.2) | 7.9 (7.5-8.3) | 0.012 |
| MMP7 | 8.7 (8.4-9.0) | 8.7 (8.3-9.0) | 8.7 (8.5-8.9) | 8.6 (8.4-9.0) | 0.758 | 8.7 (8.4-8.9) | 8.7 (8.4-9.0) | 8.7 (8.4-9.0) | 8.7 (8.5-9.0) | 0.543 |
| MMP9 | 5.1 (5.1-5.4) | 5.1 (5.1-5.2) | 5.1 (5.1-5.4) | 5.1 (5.1-5.4) | 0.654 | 5.1 (5.1-5.2) | 5.1 (5.1-5.3) | 5.1 (5.1-5.2) | 5.1 (5.1-5.5) | 0.293 |
| MPO | 3.4 (3.4-3.4) | 3.4 (3.4-3.4) | 3.4 (3.4-3.4) | 3.4 (3.4-3.4) | 0.281 | 3.4 (3.4-3.4) | 3.4 (3.4-3.4) | 3.4 (3.4-3.4) | 3.4 (3.4-3.4) | 0.382 |
| NEMO | 4.6 (3.8-5.6) | 5.0 (4.2-6.1) | 5.0 (3.9-5.7) | 4.5 (3.6-5.7) | 0.524 | 4.5 (3.6-5.5) | 5.0 (4.0-5.8) | 4.6 (3.8-5.9) | 4.8 (3.9-5.8) | 0.272 |
| NOTCH3 | 5.3 (5.1-5.7) | 5.3 (5.1-5.6) | 5.3 (5.1-5.5) | 5.3 (5.0-5.5) | 0.045 | 5.3 (5.1-5.6) | 5.3 (5.0-5.5) | 5.2 (5.0-5.5) | 5.3 (5.1-5.6) | 0.867 |
| NRTN | 0.6 (0.6-0.6) | 0.6 (0.6-0.6) | 0.6 (0.6-0.6) | 0.6 (0.6-0.6) | 0.951 | 0.6 (0.6-0.6) | 0.6 (0.6-0.6) | 0.6 (0.6-0.6) | 0.6 (0.6-0.6) | 0.580 |
| NT3 | 2.2 (1.9-2.5) | 2.2 (2.0-2.6) | 2.1 (1.8-2.4) | 2.3 (1.9-2.5) | 0.813 | 2.2 (1.9-2.5) | 2.2 (1.9-2.6) | 2.2 (1.9-2.5) | 2.1 (1.8-2.5) | 0.457 |
| OPG | 9.7 (9.6-10.0) | 9.7 (9.4-9.9) | 9.6 (9.4-9.8) | 9.6 (9.3-9.8) | 0.001 | 9.7 (9.5-10.0) | 9.7 (9.4-10.0) | 9.6 (9.4-9.8) | 9.6 (9.4-9.9) | 0.271 |
| OPN | 7.4 (7.1-7.7) | 7.4 (7.0-7.7) | 7.4 (7.0-7.7) | 7.3 (7.0-7.7) | 0.252 | 7.5 (7.1-7.7) | 7.3 (7.0-7.6) | 7.4 (6.9-7.7) | 7.4 (7.1-7.7) | 0.466 |
| OSM | 4.3 (3.6-4.9) | 4.2 (3.7-4.7) | 4.2 (3.6-4.8) | 4.3 (3.7-4.9) | 0.890 | 4.2 (3.7-4.9) | 4.3 (3.5-4.7) | 4.1 (3.6-4.8) | 4.3 (3.7-4.8) | 0.647 |
| PAI | 5.1 (4.5-5.8) | 5.3 (4.6-6.1) | 5.2 (4.5-6.0) | 5.1 (4.4-5.7) | 0.313 | 5.2 (4.4-5.9) | 5.2 (4.8-5.9) | 5.1 (4.4-5.8) | 5.2 (4.4-6.0) | 0.911 |
| PAPPA | 3.9 (3.5-4.2) | 4.0 (3.5-4.3) | 3.9 (3.6-4.2) | 3.9 (3.5-4.4) | 0.703 | 3.9 (3.4-4.3) | 3.9 (3.6-4.2) | 3.9 (3.4-4.2) | 3.9 (3.5-4.4) | 0.497 |
| PAR1 | 7.7 (7.4-8.0) | 7.7 (7.4-8.1) | 7.7 (7.5-7.9) | 7.6 (7.3-8.0) | 0.404 | 7.6 (7.3-7.9) | 7.7 (7.5-8.0) | 7.6 (7.4-7.9) | 7.8 (7.3-8.0) | 0.369 |
| PARP1 | 3.0 (2.6-3.4) | 2.9 (2.5-3.3) | 2.8 (2.5-3.2) | 2.7 (2.4-3.2) | 0.006 | 2.9 (2.5-3.2) | 2.9 (2.5-3.4) | 2.7 (2.4-3.1) | 2.8 (2.6-3.2) | 0.631 |
| PCSK9 | 3.2 (3.0-3.4) | 3.1 (2.9-3.4) | 3.2 (3.0-3.4) | 3.1 (2.9-3.4) | 0.637 | 3.1 (2.9-3.4) | 3.2 (3.0-3.5) | 3.1 (2.9-3.4) | 3.2 (2.9-3.4) | 0.983 |
| PDGFSUBa | 2.4 (1.7-3.0) | 2.7 (2.0-3.5) | 2.5 (1.8-3.1) | 2.3 (1.7-3.2) | 0.809 | 2.4 (1.7-3.1) | 2.7 (2.0-3.1) | 2.3 (1.6-3.0) | 2.5 (1.9-3.2) | 0.840 |
| PDGFSUBUb | 8.2 (7.3-9.1) | 8.8 (7.7-9.6) | 8.5 (6.9-9.3) | 8.2 (7.1-9.3) | 0.835 | 8.3 (7.2-9.3) | 8.4 (7.6-9.5) | 8.2 (6.9-9.1) | 8.5 (7.3-9.6) | 0.750 |
| PDL1 | 6.5 (6.3-6.8) | 6.5 (6.2-6.8) | 6.5 (6.2-6.7) | 6.5 (6.2-6.8) | 0.360 | 6.5 (6.2-6.8) | 6.5 (6.2-6.8) | 6.5 (6.2-6.8) | 6.4 (6.2-6.9) | 0.653 |
| PDL2 | 3.0 (2.8-3.2) | 3.0 (2.7-3.2) | 3.0 (2.8-3.3) | 3.0 (2.8-3.3) | 0.901 | 3.0 (2.8-3.2) | 3.0 (2.8-3.3) | 3.0 (2.8-3.3) | 3.0 (2.8-3.3) | 0.587 |
| PECAM1 | 4.6 (4.1-5.2) | 4.8 (4.2-5.4) | 4.6 (4.3-5.2) | 4.6 (4.2-5.1) | 0.914 | 4.6 (4.1-5.1) | 4.7 (4.3-5.2) | 4.5 (4.2-5.3) | 4.6 (4.2-5.2) | 0.534 |
| PGF | 8.3 (8.1-8.6) | 8.2 (8.0-8.5) | 8.3 (8.2-8.5) | 8.2 (8.0-8.4) | 0.031 | 8.3 (8.1-8.5) | 8.3 (8.1-8.5) | 8.2 (8.1-8.5) | 8.3 (8.1-8.6) | 0.859 |
| PGLYRP1 | 6.9 (6.6-7.2) | 6.9 (6.5-7.2) | 6.9 (6.6-7.3) | 6.9 (6.6-7.3) | 0.922 | 6.9 (6.6-7.2) | 6.8 (6.6-7.2) | 6.9 (6.5-7.2) | 7.0 (6.7-7.4) | 0.198 |
| PI3 | 2.4 (2.0-2.8) | 2.3 (2.0-2.7) | 2.3 (2.0-3.0) | 2.2 (1.9-2.7) | 0.417 | 2.3 (2.0-2.7) | 2.3 (1.9-2.8) | 2.3 (2.0-2.8) | 2.4 (2.0-3.0) | 0.697 |
| PIGR | 2.4 (2.3-2.5) | 2.4 (2.3-2.5) | 2.4 (2.3-2.5) | 2.4 (2.3-2.5) | 0.305 | 2.4 (2.3-2.5) | 2.4 (2.3-2.5) | 2.4 (2.3-2.5) | 2.4 (2.3-2.5) | 0.777 |
| PLC | 7.9 (7.7-8.1) | 7.9 (7.7-8.2) | 7.9 (7.7-8.1) | 7.9 (7.7-8.0) | 0.131 | 7.9 (7.7-8.1) | 7.9 (7.8-8.1) | 7.9 (7.6-8.1) | 7.9 (7.7-8.2) | 0.500 |
| PON3 | 6.0 (5.6-6.4) | 5.8 (5.5-6.3) | 6.0 (5.6-6.4) | 6.0 (5.7-6.4) | 0.227 | 5.9 (5.5-6.3) | 6.0 (5.6-6.4) | 6.0 (5.5-6.3) | 6.0 (5.6-6.3) | 0.906 |
| PRELP | 7.4 (7.3-7.5) | 7.4 (7.2-7.5) | 7.4 (7.3-7.5) | 7.4 (7.2-7.5) | 0.217 | 7.4 (7.2-7.5) | 7.4 (7.3-7.5) | 7.4 (7.3-7.5) | 7.4 (7.2-7.5) | 0.786 |
| PRSS27 | 8.6 (8.2-8.9) | 8.4 (8.1-8.8) | 8.5 (8.2-8.9) | 8.5 (8.1-8.8) | 0.339 | 8.5 (8.2-8.9) | 8.5 (8.1-8.9) | 8.5 (8.1-8.8) | 8.5 (8.1-8.9) | 0.620 |
| PRSS8 | 8.6 (8.2-8.8) | 8.5 (8.3-8.8) | 8.6 (8.4-8.8) | 8.5 (8.3-8.7) | 0.753 | 8.6 (8.3-8.7) | 8.5 (8.3-8.7) | 8.6 (8.3-8.8) | 8.6 (8.3-8.8) | 0.183 |
| PSGL1 | 3.8 (3.7-4.0) | 3.8 (3.7-4.0) | 3.8 (3.7-4.0) | 3.8 (3.6-4.0) | 0.447 | 3.8 (3.7-4.0) | 3.8 (3.7-4.0) | 3.8 (3.6-4.0) | 3.8 (3.6-3.9) | 0.203 |
| PSPD | 3.2 (2.8-3.7) | 3.3 (2.8-3.8) | 3.4 (2.9-3.9) | 3.3 (2.7-3.9) | 0.727 | 3.2 (2.8-3.8) | 3.3 (2.8-3.7) | 3.3 (2.9-3.8) | 3.3 (2.9-3.8) | 0.215 |
| PTX3 | 4.0 (3.8-4.3) | 3.9 (3.6-4.2) | 4.0 (3.8-4.3) | 4.0 (3.8-4.3) | 0.300 | 4.0 (3.8-4.3) | 4.0 (3.8-4.3) | 4.0 (3.8-4.2) | 3.9 (3.7-4.2) | 0.451 |
| RAGE | 12.4 (12.1-12.6) | 12.3 (12.1-12.6) | 12.3 (12.1-12.6) | 12.2 (11.9-12.6) | 0.090 | 12.3 (12.1-12.6) | 12.3 (12.0-12.6) | 12.4 (12.1-12.6) | 12.3 (12.1-12.7) | 0.688 |
| RARRES2 | 11.2 (10.9-11.3) | 11.2 (10.9-11.4) | 11.1 (10.9-11.4) | 11.1 (10.9-11.3) | 0.209 | 11.1 (10.9-11.3) | 11.1 (10.9-11.4) | 11.1 (10.9-11.3) | 11.1 (11.0-11.3) | 0.241 |
| REN | 7.1 (6.4-7.7) | 7.0 (6.5-7.6) | 7.0 (6.5-7.6) | 7.0 (6.4-7.5) | 0.632 | 7.0 (6.4-7.5) | 7.1 (6.4-7.6) | 7.1 (6.6-7.8) | 7.0 (6.4-7.6) | 0.553 |
| RETN | 6.0 (5.7-6.4) | 6.1 (5.8-6.5) | 6.0 (5.8-6.4) | 6.0 (5.7-6.4) | 0.426 | 6.0 (5.7-6.4) | 6.0 (5.7-6.4) | 6.0 (5.7-6.4) | 6.1 (5.8-6.6) | 0.096 |
| SCF | 9.5 (9.1-9.7) | 9.4 (9.2-9.6) | 9.4 (9.2-9.6) | 9.4 (9.2-9.6) | 0.658 | 9.4 (9.2-9.6) | 9.4 (9.1-9.6) | 9.4 (9.3-9.6) | 9.5 (9.2-9.7) | 0.237 |
| SCGB3A2 | 2.4 (2.0-2.9) | 2.5 (2.0-2.9) | 2.5 (2.0-3.0) | 2.3 (1.8-3.0) | 0.567 | 2.4 (2.0-2.9) | 2.4 (1.9-2.7) | 2.4 (2.0-2.9) | 2.5 (2.0-2.9) | 0.826 |
| SELE | 11.6 (11.0-12.0) | 11.5 (11.2-11.9) | 11.5 (11.1-11.9) | 11.5 (10.9-11.9) | 0.343 | 11.5 (11.1-12.0) | 11.6 (11.1-12.0) | 11.5 (11.0-11.9) | 11.5 (11.2-11.9) | 0.562 |
| SELP | 9.7 (9.3-10.2) | 9.9 (9.3-10.5) | 9.8 (9.3-10.3) | 9.6 (9.1-10.4) | 0.643 | 9.7 (9.2-10.2) | 9.8 (9.4-10.3) | 9.6 (9.1-10.4) | 9.6 (9.2-10.4) | 0.980 |
| SERPINA12 | 3.3 (3.3-3.4) | 3.3 (3.3-3.3) | 3.3 (3.3-3.5) | 3.3 (3.3-3.4) | 0.662 | 3.3 (3.3-3.5) | 3.3 (3.3-3.3) | 3.3 (3.3-3.5) | 3.3 (3.3-3.5) | 0.642 |
| SHPS1 | 3.5 (3.2-3.8) | 3.6 (3.3-3.8) | 3.5 (3.2-3.8) | 3.5 (3.2-3.7) | 0.889 | 3.5 (3.2-3.8) | 3.6 (3.3-3.8) | 3.5 (3.3-3.8) | 3.6 (3.2-3.7) | 0.511 |
| SIRT2 | 4.0 (3.2-5.1) | 4.3 (3.4-5.3) | 4.4 (3.5-5.1) | 4.0 (3.1-5.2) | 0.490 | 3.9 (3.2-4.8) | 4.2 (3.6-5.2) | 4.2 (3.2-5.2) | 4.3 (3.3-5.1) | 0.157 |
| SLAMF1 | 3.0 (2.6-3.3) | 2.8 (2.5-3.3) | 2.8 (2.5-3.2) | 2.7 (2.4-3.0) | 0.001 | 2.9 (2.5-3.3) | 2.8 (2.5-3.2) | 2.8 (2.6-3.2) | 2.8 (2.5-3.2) | 0.432 |
| SLAMF7 | 3.2 (2.8-3.6) | 3.1 (2.7-3.6) | 3.0 (2.6-3.4) | 3.1 (2.6-3.4) | 0.015 | 3.2 (2.7-3.6) | 3.2 (2.9-3.6) | 3.0 (2.6-3.4) | 3.1 (2.6-3.5) | 0.045 |
| SOD2 | 8.6 (8.5-8.7) | 8.6 (8.5-8.7) | 8.6 (8.5-8.7) | 8.6 (8.5-8.7) | 0.132 | 8.6 (8.5-8.7) | 8.6 (8.5-8.7) | 8.6 (8.5-8.7) | 8.6 (8.5-8.7) | 0.418 |
| SORT1 | 8.2 (8.0-8.3) | 8.2 (8.0-8.4) | 8.2 (8.0-8.4) | 8.2 (8.0-8.3) | 0.453 | 8.2 (8.0-8.3) | 8.2 (8.0-8.4) | 8.2 (8.0-8.4) | 8.1 (8.0-8.4) | 0.310 |
| SPON1 | 1.8 (1.6-2.1) | 1.8 (1.6-1.9) | 1.7 (1.5-1.9) | 1.7 (1.5-1.9) | 0.005 | 1.7 (1.5-1.9) | 1.7 (1.5-1.9) | 1.7 (1.5-1.9) | 1.8 (1.6-2.0) | 0.473 |
| SPON2 | 8.6 (8.5-8.8) | 8.6 (8.5-8.8) | 8.6 (8.5-8.7) | 8.6 (8.5-8.7) | 0.180 | 8.6 (8.5-8.8) | 8.6 (8.5-8.7) | 8.6 (8.5-8.8) | 8.7 (8.5-8.8) | 0.408 |
| SRC | 6.4 (5.6-6.9) | 6.6 (5.8-7.0) | 6.6 (5.6-7.0) | 6.2 (5.2-6.8) | 0.296 | 6.3 (5.2-6.9) | 6.6 (5.7-7.0) | 6.3 (5.3-7.0) | 6.5 (5.5-7.0) | 0.376 |
| ST1A1 | 2.4 (2.0-3.3) | 2.6 (2.0-3.4) | 2.5 (2.0-3.5) | 2.2 (2.0-3.1) | 0.047 | 2.4 (2.0-3.1) | 2.5 (2.0-3.4) | 2.5 (2.0-3.3) | 2.3 (2.0-3.4) | 0.673 |
| ST2 | 4.3 (4.0-4.6) | 4.3 (4.0-4.7) | 4.2 (3.9-4.6) | 4.2 (4.0-4.5) | 0.629 | 4.2 (4.0-4.6) | 4.2 (4.0-4.6) | 4.3 (3.9-4.5) | 4.3 (4.0-4.7) | 0.854 |
| STAMBP | 4.9 (4.5-5.7) | 5.1 (4.6-5.9) | 5.1 (4.6-5.7) | 4.9 (4.4-5.7) | 0.713 | 4.9 (4.4-5.5) | 5.1 (4.6-5.7) | 5.0 (4.5-5.7) | 5.0 (4.5-5.8) | 0.143 |
| STK4 | 3.7 (2.8-4.9) | 4.3 (3.4-5.1) | 4.2 (3.1-5.0) | 3.7 (2.7-4.8) | 0.510 | 3.7 (2.5-4.7) | 4.3 (3.1-5.0) | 3.8 (2.7-5.1) | 3.9 (3.0-4.9) | 0.253 |
| TF | 5.2 (5.0-5.4) | 5.2 (4.9-5.4) | 5.2 (5.0-5.4) | 5.2 (4.9-5.4) | 0.280 | 5.2 (4.9-5.4) | 5.2 (4.9-5.4) | 5.2 (5.0-5.4) | 5.2 (5.1-5.4) | 0.206 |
| TFF3 | 5.2 (4.9-5.5) | 5.1 (4.8-5.4) | 5.1 (4.9-5.5) | 5.1 (4.8-5.3) | 0.220 | 5.1 (4.8-5.5) | 5.1 (4.9-5.4) | 5.0 (4.8-5.4) | 5.1 (4.9-5.5) | 0.780 |
| TFPI | 8.6 (8.3-8.8) | 8.6 (8.3-8.8) | 8.6 (8.4-8.8) | 8.6 (8.4-8.8) | 0.680 | 8.6 (8.3-8.8) | 8.6 (8.3-8.8) | 8.5 (8.3-8.8) | 8.6 (8.4-8.8) | 0.196 |
| TGFALPHA | 4.2 (4.0-4.4) | 4.1 (3.9-4.4) | 4.1 (3.9-4.3) | 4.0 (3.9-4.3) | 0.011 | 4.2 (3.9-4.4) | 4.1 (3.9-4.4) | 4.1 (3.9-4.3) | 4.1 (3.9-4.4) | 0.558 |
| TGM2 | 8.2 (7.7-8.8) | 8.2 (7.7-8.7) | 8.2 (7.6-8.8) | 8.2 (7.8-8.8) | 0.576 | 8.2 (7.7-8.8) | 8.3 (7.7-8.8) | 8.3 (7.9-8.7) | 8.1 (7.6-8.8) | 0.868 |
| THBS2 | 5.0 (4.9-5.2) | 5.0 (4.8-5.2) | 5.0 (4.8-5.1) | 5.0 (4.8-5.1) | 0.001 | 5.0 (4.9-5.2) | 5.0 (4.8-5.2) | 5.0 (4.8-5.1) | 5.0 (4.8-5.1) | <0.001 |
| THPO | 2.1 (1.9-2.4) | 2.1 (1.9-2.4) | 2.1 (1.9-2.4) | 2.1 (1.8-2.3) | 0.458 | 2.1 (1.9-2.3) | 2.1 (1.9-2.4) | 2.1 (1.9-2.4) | 2.1 (1.9-2.4) | 0.612 |
| TIE2 | 6.9 (6.8-7.1) | 6.9 (6.7-7.1) | 6.9 (6.7-7.1) | 6.9 (6.7-7.1) | 0.875 | 6.9 (6.7-7.1) | 6.9 (6.7-7.1) | 6.9 (6.7-7.1) | 6.9 (6.7-7.1) | 0.385 |
| TIMP4 | 3.3 (3.0-3.7) | 3.3 (3.0-3.7) | 3.2 (2.9-3.5) | 3.3 (3.0-3.5) | 0.171 | 3.2 (2.9-3.5) | 3.2 (3.0-3.7) | 3.1 (2.9-3.5) | 3.4 (3.1-3.7) | 0.038 |
| TLT2 | 5.0 (4.7-5.3) | 5.1 (4.6-5.3) | 5.0 (4.7-5.3) | 5.0 (4.5-5.2) | 0.372 | 5.0 (4.6-5.2) | 5.0 (4.7-5.3) | 5.0 (4.6-5.3) | 5.0 (4.7-5.4) | 0.341 |
| TM | 9.8 (9.6-10.1) | 9.8 (9.6-10.1) | 9.9 (9.6-10.1) | 9.8 (9.6-10.1) | 0.826 | 9.8 (9.6-10.1) | 9.8 (9.5-10.0) | 9.8 (9.7-10.0) | 9.9 (9.6-10.1) | 0.051 |
| TNFB | 4.8 (4.6-5.1) | 4.7 (4.4-5.0) | 4.7 (4.5-5.0) | 4.7 (4.4-4.9) | 0.003 | 4.8 (4.5-5.0) | 4.8 (4.6-5.0) | 4.7 (4.4-4.9) | 4.7 (4.4-4.9) | 0.014 |
| TNFR1 | 6.6 (6.4-6.9) | 6.6 (6.4-6.9) | 6.6 (6.3-6.8) | 6.5 (6.2-6.8) | 0.013 | 6.5 (6.3-6.8) | 6.5 (6.3-6.8) | 6.5 (6.3-6.8) | 6.6 (6.4-7.0) | 0.247 |
| TNFR2 | 5.8 (5.5-6.1) | 5.8 (5.5-6.1) | 5.8 (5.4-6.1) | 5.7 (5.5-6.0) | 0.183 | 5.8 (5.4-6.1) | 5.7 (5.4-6.0) | 5.7 (5.4-6.0) | 5.8 (5.5-6.2) | 0.316 |
| TNFRSF1 | 3.2 (3.0-3.5) | 3.3 (3.0-3.5) | 3.2 (3.1-3.5) | 3.2 (2.9-3.5) | 0.46 | 3.2 (3.0-3.4) | 3.3 (3.0-3.5) | 3.2 (3.0-3.5) | 3.2 (3.0-3.5) | 0.957 |
| TNFRSF10c | 6.7 (6.4-6.9) | 6.6 (6.3-6.8) | 6.7 (6.3-6.9) | 6.6 (6.3-7.0) | 0.982 | 6.7 (6.3-6.9) | 6.6 (6.3-6.8) | 6.6 (6.3-6.9) | 6.7 (6.3-7.1) | 0.195 |
| TNFRSF11a | 5.5 (5.2-6.0) | 5.5 (5.2-5.9) | 5.5 (5.3-5.8) | 5.4 (5.1-5.7) | 0.061 | 5.5 (5.2-5.8) | 5.5 (5.2-5.8) | 5.5 (5.2-5.8) | 5.5 (5.3-5.9) | 0.100 |
| TNFRSF13b | 8.3 (8.1-8.6) | 8.3 (8.1-8.7) | 8.3 (8.0-8.7) | 8.2 (8.0-8.5) | 0.015 | 8.3 (8.1-8.7) | 8.4 (8.1-8.7) | 8.2 (8.0-8.5) | 8.3 (8.0-8.6) | 0.200 |
| TNFRSF14 | 4.8 (4.5-5.2) | 4.8 (4.6-5.2) | 4.8 (4.5-5.1) | 4.7 (4.4-5.0) | 0.232 | 4.8 (4.5-5.1) | 4.8 (4.5-5.1) | 4.8 (4.5-5.1) | 4.8 (4.5-5.2) | 0.170 |
| TNFRSF9 | 6.9 (6.5-7.3) | 6.8 (6.4-7.1) | 6.8 (6.6-7.0) | 6.8 (6.5-7.0) | 0.047 | 6.8 (6.5-7.2) | 6.8 (6.5-7.1) | 6.8 (6.5-7.0) | 6.8 (6.4-7.1) | 0.296 |
| TNFSF13B | 6.9 (6.7-7.2) | 7.0 (6.8-7.2) | 6.9 (6.7-7.2) | 6.9 (6.6-7.1) | 0.217 | 6.9 (6.7-7.2) | 6.9 (6.7-7.2) | 6.9 (6.7-7.1) | 6.9 (6.7-7.2) | 0.867 |
| TNFSF14 | 3.3 (2.9-3.7) | 3.3 (3.0-3.7) | 3.2 (3.0-3.5) | 3.2 (2.8-3.6) | 0.101 | 3.2 (2.9-3.6) | 3.2 (2.9-3.6) | 3.1 (2.8-3.6) | 3.3 (3.0-3.6) | 0.957 |
| TPA | 6.8 (6.4-7.2) | 6.9 (6.5-7.3) | 6.8 (6.3-7.3) | 6.9 (6.5-7.5) | 0.429 | 6.7 (6.4-7.2) | 6.9 (6.4-7.4) | 6.8 (6.3-7.2) | 6.8 (6.4-7.5) | 0.582 |
| TR | 5.4 (4.9-5.8) | 5.3 (4.9-5.7) | 5.2 (4.9-5.8) | 5.3 (4.9-5.8) | 0.766 | 5.4 (4.9-5.8) | 5.3 (5.0-5.7) | 5.3 (4.9-5.6) | 5.3 (5.0-6.0) | 0.480 |
| TRAIL | 8.4 (8.1-8.6) | 8.3 (8.1-8.4) | 8.3 (8.1-8.5) | 8.4 (8.2-8.5) | 0.942 | 8.3 (8.1-8.5) | 8.4 (8.1-8.5) | 8.3 (8.1-8.5) | 8.3 (8.1-8.5) | 0.339 |
| TRAILR2 | 5.8 (5.5-6.0) | 5.8 (5.5-6.0) | 5.8 (5.6-6.0) | 5.7 (5.4-6.0) | 0.164 | 5.8 (5.5-6.0) | 5.8 (5.5-6.0) | 5.7 (5.5-6.0) | 5.8 (5.5-6.1) | 0.650 |
| TRANCE | 4.5 (4.1-4.8) | 4.4 (4.0-4.8) | 4.4 (4.0-4.9) | 4.5 (4.0-4.8) | 0.730 | 4.4 (4.0-4.8) | 4.5 (4.1-4.8) | 4.4 (4.0-4.8) | 4.5 (4.0-4.8) | 0.942 |
| TRAP | 3.6 (3.3-3.8) | 3.6 (3.4-3.8) | 3.6 (3.3-3.8) | 3.6 (3.4-3.8) | 0.257 | 3.6 (3.4-3.7) | 3.5 (3.3-3.7) | 3.6 (3.3-3.8) | 3.6 (3.4-3.8) | 0.121 |
| TWEAK | 9.1 (8.9-9.4) | 9.2 (9.0-9.4) | 9.1 (9.0-9.4) | 9.1 (8.9-9.4) | 0.757 | 9.1 (8.9-9.4) | 9.2 (9.0-9.4) | 9.2 (9.0-9.4) | 9.1 (8.9-9.4) | 0.855 |
| UPA | 9.3 (9.1-9.5) | 9.2 (9.0-9.5) | 9.2 (9.0-9.4) | 9.2 (9.1-9.4) | 0.083 | 9.3 (9.0-9.5) | 9.3 (9.0-9.5) | 9.2 (9.0-9.4) | 9.2 (9.1-9.4) | 0.285 |
| UPAR | 5.2 (5.1-5.5) | 5.2 (4.9-5.5) | 5.2 (5.0-5.5) | 5.2 (4.9-5.4) | 0.114 | 5.2 (5.0-5.5) | 5.2 (4.9-5.4) | 5.2 (4.9-5.5) | 5.3 (5.0-5.5) | 0.254 |
| VEGFA | 9.8 (9.5-10.0) | 9.7 (9.5-10.0) | 9.7 (9.5-9.9) | 9.7 (9.5-10.0) | 0.090 | 9.8 (9.5-10.0) | 9.7 (9.5-10.1) | 9.7 (9.5-9.9) | 9.8 (9.5-10.0) | 0.928 |
| VEGFD | 7.4 (7.1-7.7) | 7.3 (7.1-7.6) | 7.4 (7.1-7.6) | 7.3 (7.0-7.5) | 0.016 | 7.4 (7.1-7.7) | 7.3 (7.1-7.6) | 7.3 (7.1-7.5) | 7.3 (7.1-7.5) | 0.733 |
| VSIG2 | 4.4 (4.0-5.0) | 4.4 (3.9-4.8) | 4.6 (4.2-4.9) | 4.3 (3.9-4.9) | 0.744 | 4.4 (4.0-4.9) | 4.4 (4.0-5.0) | 4.5 (4.2-4.9) | 4.4 (4.0-4.9) | 0.802 |
| VWF | 7.3 (6.4-8.3) | 7.6 (6.9-8.3) | 7.5 (6.7-8.3) | 7.7 (6.8-8.6) | 0.134 | 7.5 (6.7-8.2) | 7.4 (6.6-8.4) | 7.5 (6.9-8.1) | 7.5 (6.5-8.5) | 0.745 |
| XCL1 | 5.1 (4.7-5.3) | 5.0 (4.6-5.3) | 5.0 (4.6-5.4) | 4.9 (4.6-5.2) | 0.085 | 5.0 (4.6-5.3) | 5.0 (4.6-5.3) | 5.0 (4.7-5.3) | 5.0 (4.6-5.3) | 0.925 |

Abreviations as in table S1. Values are expressed as median (interquartile range)

**Supplemental table 3. Demographic, clinical and biochemical characteristics at baseline in the training and test sets at 1 and 9 months in patients from the HOMAGE trial**

|  | **1 month** | | |  | **9 months** | | |
| --- | --- | --- | --- | --- | --- | --- | --- |
|  | **Train (n=390)** | **Test (n=91)** | **P value** |  | **Train (n=397)** | **Test (n=91)** | **P value** |
| Demographics |  |  |  |  |  |  |  |
| Age, years | 72 (68-78) | 71 (66-77) | 0.097 |  | 72 (68-78) | 72 (67-78) | 0.416 |
| Men, n (%) | 285 (73.1) | 76 (83.5) | 0.038 |  | 300 (75.6) | 67 (73.6) | 0.699 |
| Current smoker, n (%) | 35 (9) | 5 (5.5) | 0.266 |  | 33 (8.3) | 3 (3.3) | 0.100 |
| Prior Medical History, n (%) |  |  |  |  |  |  |  |
| Hypertension | 309 (79.2) | 66 (72.5) | 0.165 |  | 307 (77.3) | 74 (81.3) | 0.407 |
| Diabetes mellitus | 150 (38.5) | 40 (44) | 0.334 |  | 155 (39) | 38 (41.8) | 0.633 |
| Coronary artery disease | 282 (72.3) | 68 (74.7) | 0.641 |  | 289 (72.8) | 65 (71.4) | 0.792 |
| Myocardial infarction | 158 (40.5) | 38 (41.8) | 0.828 |  | 166 (41.8) | 36 (39.6) | 0.694 |
| Percutaneous Coronary Intervention | 199 (51) | 46 (50.5) | 0.935 |  | 205 (51.6) | 45 (49.5) | 0.707 |
| Coronary Artery Bypass Graft | 99 (25.4) | 32 (35.2) | 0.059 |  | 105 (26.4) | 25 (27.5) | 0.842 |
| Stroke/Transient Ischemic Attack | 16 (4.1) | 7 (7.7) | 0.148 |  | 19 (4.8) | 2 (2.2) | 0.272 |
| COPD | 27 (6.9) | 4 (4.4) | 0.377 |  | 27 (6.8) | 2 (2.2) | 0.094 |
| Randomized treatment, n (%) |  |  |  |  |  |  |  |
| Spironolactone | 195 (50) | 44 (48.4) | 0.777 |  | 195 (49.1) | 51 (56) | 0.233 |
| Other baseline medications, n (%) |  |  |  |  |  |  |  |
| ACE inhibitor | 200 (51.3) | 51 (56) | 0.413 |  | 208 (52.4) | 46 (50.5) | 0.751 |
| Angiotensin Receptor Blockers | 114 (29.2) | 17 (18.7) | 0.042 |  | 108 (27.2) | 27 (29.7) | 0.635 |
| Beta-blockers | 264 (67.7) | 69 (75.8) | 0.130 |  | 274 (69) | 68 (74.7) | 0.284 |
| Thiazide Diuretics | 68 (17.4) | 10 (11) | 0.133 |  | 66 (16.6) | 15 (16.5) | 0.974 |
| Calcium channel blocker | 91 (23.3) | 11 (12.1) | 0.018 |  | 77 (19.4) | 24 (26.4) | 0.138 |
| Lipid-lowering therapy | 317 (81.3) | 78 (85.7) | 0.320 |  | 322 (81.1) | 81 (89) | 0.073 |
| Aspirin | 278 (71.3) | 66 (72.5) | 0.813 |  | 274 (69) | 71 (78) | 0.089 |
| Any antiplatelet therapy | 306 (78.5) | 73 (80.2) | 0.712 |  | 308 (77.6) | 72 (79.1) | 0.750 |
| Physical examination |  |  |  |  |  |  |  |
| BMI, kg/m^2^ | 28.1 (25.4-31.6) | 28.1 (25.1-31.2) | 0.675 |  | 28.0 (25.4-31.2) | 28.7 (25.2-32.5) | 0.294 |
| HR, beats/min | 61.0 (54.0-67.0) | 59.0 (55.0-66.0) | 0.581 |  | 60.0 (54.0-67.0) | 61.0 (55.0-68.0) | 0.909 |
| SBP, mmHg | 141 (128-156) | 139 (128-152) | 0.441 |  | 140 (128-154) | 144 (127-154) | 0.660 |
| DBP, mmHg | 78 (71-85) | 78 (72-84) | 0.920 |  | 78 (71-84) | 77 (71-85) | 0.694 |
| Breathlessness scale | 5.0 (3.0-7.0) | 5.0 (4.0-7.0) | 0.114 |  | 5.0 (3.0-7.0) | 5.0 (3.0-7.0) | 0.872 |
| Blood tests |  |  |  |  |  |  |  |
| Hemoglobin, g/dL | 14.0 (13.1-14.9) | 14.3 (13.3-15.1) | 0.129 |  | 14.0 (13.2-14.9) | 14.0 (13.0-14.8) | 0.689 |
| Sodium, mmol/L | 140 (138-141) | 139 (138-141) | 0.696 |  | 140 (138-141) | 139 (137-141) | 0.349 |
| Potassium, mmol/L | 4.3 (4.1-4.6) | 4.4 (4.1-4.6) | 0.105 |  | 4.3 (4.1-4.6) | 4.2 (4.0-4.5) | 0.094 |
| eGFR, mL/min/1.73 m^2^ | 76 (62-86) | 70 (62-84) | 0.221 |  | 74 (62-85) | 77 (63-87) | 0.323 |
| Electrocardiography |  |  |  |  |  |  |  |
| QRS duration (ms) | 92 (84-106) | 90 (82-104) | 0.505 |  | 92 (84-106) | 96 (82-107) | 0.632 |
| Echocardiography |  |  |  |  |  |  |  |
| LVEDVi, mL/m^2^ | 42.5 (35.7-49.1) | 40.5 (34.9-47.0) | 0.353 |  | 41.7 (35.5-48.7) | 42.8 (36.5-48.9) | 0.698 |
| LVEF, % | 63.1 (58.1-66.6) | 62.9 (57.4-66.4) | 0.864 |  | 63.3 (58.4-66.9) | 61.7 (56.5-65.3) | 0.203 |
| LVMI, g/m^2^ | 94.3 (80.6-113.6) | 93.2 (79.3-108.1) | 0.539 |  | 94.0 (81.5-113.1) | 94.7 (78.7-108.4) | 0.583 |
| LAVI, mL/m^2^ | 30.8 (26.2-36.6) | 29.6 (25.6-34.8) | 0.139 |  | 30.5 (26.0-36.6) | 31.5 (26.6-36.1) | 0.543 |
| E:A ratio | 0.8 (0.7-1.0) | 0.8 (0.6-1.0) | 0.446 |  | 0.8 (0.7-1.0) | 0.9 (0.7-1.1) | 0.019 |
| E:e’ ratio | 9.3 (7.6-11.5) | 8.9 (7.4-10.9) | 0.403 |  | 9.3 (7.5-11.4) | 9.7 (8.0-11.9) | 0.272 |
| TAPSE | 22.3 (16.7-26.7) | 20.0 (17.6-25.8) | 0.544 |  | 21.6 (16.7-26.5) | 23.0 (19.0-26.9) | 0.381 |
| Blood biomarkers |  |  |  |  |  |  |  |
| NT-proBNP, ng/L | 210 (134-338) | 194 (130-339) | 0.592 |  | 207 (130-337) | 219 (154-350) | 0.259 |
| Hs-TnT, ng/L | 12.8 (8.9-17.7) | 12.4 (8.2-16.6) | 0.178 |  | 12.4 (8.7-17.3) | 13.4 (8.5-18.6) | 0.565 |
| PICP, µg/L | 80.0 (64.5-96.1) | 80.6 (67.2-97.1) | 0.501 |  | 80.5 (65.1-96.8) | 80.5 (67.5-96.7) | 0.689 |
| PIIINP, µg/L | 3.9 (3.1-4.9) | 4.0 (3.1-5.2) | 0.614 |  | 3.9 (3.1-5.0) | 4.0 (3.1-5.0) | 0.912 |
| Galectin-3, µg/L | 15.9 (13.5-19.7) | 15.8 (13.0-18.8) | 0.557 |  | 16.2 (13.6-19.9) | 15.5 (12.8-18.9) | 0.103 |
| GDF-15, µg/L | 1415 (1036-2147) | 1459 (1047-1954) | 0.925 |  | 1412(1032-2115) | 1597(1051-2078) | 0.478 |

PICP means procollagen type I C-terminal propeptide; PCI, percutaneous coronary intervention; CABG, coronary artery bypass graft; STIA, stroke/transient ischemic attach; COPD, chronic obstructive pulmonary disease; ACE, angiotensin converting enzyme; ARB, angiotensin II receptor blocker; BMI, body mass index; HR, heart rate; SBP, systolic blood pressure; DBP, diastolic blood pressure; eGFR, estimated glomerular filtration rate; LVEDVi, left ventricular (LV) end-diastolic volume index; LVEF, LV ejection fraction; LVMI, LV mass index; LAVI, left atrial volume index; E, early mitral flow velocity; A, late (atrial) mitral flow velocity; e’, early diastolic tissue velocity; TAPSE: tricuspid annular plane systolic excursion; NT-proBNP; N-terminal pro-brain natriuretic peptide; Hs-TnT, high sensitivity troponin T; PIIINP, procollagen type III N-terminal propeptide; GDF-15, growth differentiation factor 15.Values are expressed as median (interquartile range) and categorical variables as numbers and percentages.

**Supplemental table 4. Difference (versus baseline) in the ML-selected proteins that changed in parallel with PICP at 1 and 9 months, in control and spironolactone-treated patients from the HOMAGE trial**

|  | **1 month** | | |  | **9 months** | | |
| --- | --- | --- | --- | --- | --- | --- | --- |
|  | **Marginal mean** | **95%CI** | **P value** |  | **Marginal mean** | **95%CI** | **P value** |
| **Proteins selected at 1 and 9 months** |  |  |  |  |  |  |  |
| **CCL24** |  |  |  |  |  |  |  |
| Control | -0.07 | -0.12 to -0.03 | 0.002 |  | -0.07 | -0.14 to -0.01 | 0.024 |
| Spironolactone | -0.02 | -0.07 to 0.03 | 0.42 |  | -0.04 | -0.10 to 0.02 | 0.21 |
| **DNER** |  |  |  |  |  |  |  |
| Control | -0.001 | -0.04 to 0.04 | 0.96 |  | 0.02 | -0.02 to 0.05 | 0.40 |
| Spironolactone | 0.01 | -0.02 to 0.05 | 0.44 |  | 0.001 | -0.04 to 0.04 | 0.97 |
| **FABP4** |  |  |  |  |  |  |  |
| Control | 0.008 | -0.05 to 0.07 | 0.80 |  | 0.03 | -0.05 to 0.12 | 0.44 |
| Spironolactone | 0.10 | 0.04 to 0.17 | 0.001 |  | 0.13 | 0.04 to 0.21 | 0.003 |
| **FLT3L** |  |  |  |  |  |  |  |
| Control | -0.01 | -0.06 to 0.03 | 0.52 |  | 0.009 | -0.03 to 0.05 | 0.67 |
| Spironolactone | -0.002 | -0.05 to 0.05 | 0.93 |  | 0.003 | -0.05 to 0.05 | 0.90 |
| **GAL9** |  |  |  |  |  |  |  |
| Control | -0.01 | -0.05 to 0.02 | 0.39 |  | 0.009 | -0.03 to 0.05 | 0.65 |
| Spironolactone | 0.01 | -0.02 to 0.04 | 0.52 |  | 0.04 | 0.003 to 0.09 | **0.035** |
| **IL6RA** |  |  |  |  |  |  |  |
| Control | -0.06 | -0.10 to -0.01 | 0.014 |  | -0.03 | -0.07 to 0.02 | 0.22 |
| Spironolactone | -0.02 | -0.06 to 0.02 | 0.38 |  | 0.05 | 0.003 to 0.09 | 0.037 |
| **THBS2** |  |  |  |  |  |  |  |
| Control | -0.009 | -0.03 to 0.02 | 0.51 |  | 0.001 | -0.03 to 0.04 | 0.96 |
| Spironolactone | -0.05 | -0.07 to -0.02 | 0.001 |  | -0.04 | -0.08 to -0.01 | 0.011 |
| **Proteins selected at 1 month** |  |  |  |  |  |  |  |
| **CCL25** |  |  |  |  |  |  |  |
| Control | -0.01 | -0.05 to 0.02 | 0.40 |  | 0.04 | -0.004 to 0.09 | 0.08 |
| Spironolactone | 0.09 | 0.05 to 0.12 | <0.001 |  | 0.12 | 0.08 to 0.17 | <0.001 |
| **CDCP1** |  |  |  |  |  |  |  |
| Control | 0.01 | -0.04 to 0.05 | 0.76 |  | 0.04 | -0.02 to 0.10 | 0.19 |
| Spironolactone | -0.01 | -0.06 to 0.03 | 0.52 |  | -0.03 | -0.09 to 0.03 | 0.40 |
| **FGF21** |  |  |  |  |  |  |  |
| Control | 0.06 | -0.03 to 0.15 | 0.19 |  | 0.12 | -0.01 to 0.24 | 0.06 |
| Spironolactone | -0.02 | -0.11 to 0.07 | 0.66 |  | 0.09 | -0.03 to 0.21 | 0.15 |
| **LEP** |  |  |  |  |  |  |  |
| Control | -0.03 | -0.09 to 0.03 | 0.28 |  | 0.05 | -0.03 to 0.13 | 0.19 |
| Spironolactone | 0.11 | 0.05 to 0.17 | <0.001 |  | 0.12 | 0.04 to 0.19 | 0.004 |
| **MB** |  |  |  |  |  |  |  |
| Control | 0.05 | -0.01 to 0.11 | 0.12 |  | 0.006 | -0.08 to 0.09 | 0.89 |
| Spironolactone | -0.05 | -0.12 to 0.01 | 0.09 |  | 0.003 | -0.08 to 0.09 | 0.94 |
| **MEPE** |  |  |  |  |  |  |  |
| Control | -0.02 | -0.07 to 0.03 | 0.42 |  | 0.004 | -0.04 to 0.05 | 0.85 |
| Spironolactone | 0.03 | -0.01 to 0.08 | 0.15 |  | 0.03 | -0.02 to 0.08 | 0.19 |
| **MERTK** |  |  |  |  |  |  |  |
| Control | -0.04 | -0.09 to 0.01 | 0.10 |  | 0.007 | -0.04 to 0.06 | 0.78 |
| Spironolactone | -0.05 | -0.10 to -0.004 | 0.031 |  | -0.02 | -0.07 to 0.03 | 0.35 |
| **NOTCH3** |  |  |  |  |  |  |  |
| Control | -0.01 | -0.05 to 0.03 | 0.59 |  | 0.03 | -0.02 to 0.08 | 0.24 |
| Spironolactone | -0.09 | -0.13 to -0.05 | <0.001 |  | -0.07 | -0.13 to -0.02 | 0.005 |
| **PGLYRP1** |  |  |  |  |  |  |  |
| Control | -0.03 | -0.09 to 0.02 | 0.21 |  | -0.01 | -0.08 to 0.06 | 0.72 |
| Spironolactone | 0.002 | -0.05 to 0.06 | 0.95 |  | 0.09 | 0.02 to 0.16 | 0.015 |
| **PON3** |  |  |  |  |  |  |  |
| Control | -0.007 | -0.06 to 0.04 | 0.80 |  | -0.004 | -0.06 to 0.05 | 0.88 |
| Spironolactone | 0.03 | -0.02 to 0.08 | 0.24 |  | 0.02 | -0.03 to 0.08 | 0.40 |
| **REN** |  |  |  |  |  |  |  |
| Control | -0.04 | -0.11 to 0.03 | 0.24 |  | -0.02 | -0.12 to 0.07 | 0.61 |
| Spironolactone | 0.41 | 0.35 to 0.48 | <0.001 |  | 0.48 | 0.38 to 0.57 | <0.001 |
| **SIRT2** |  |  |  |  |  |  |  |
| Control | 0.01 | -0.17 to 0.20 | 0.88 |  | 0.04 | -0.18 to 0.27 | 0.70 |
| Spironolactone | 0.005 | -0.18 to 0.19 | 0.96 |  | -0.06 | -0.28 to 0.17 | 0.63 |
| **VEGFD** |  |  |  |  |  |  |  |
| Control | -0.007 | -0.04 to -0.03 | 0.68 |  | 0.008 | -0.03 to 0.05 | 0.70 |
| Spironolactone | -0.09 | -0.12 to -0.06 | <0.001 |  | -0.06 | -0.11 to -0.02 | 0.004 |
| **Proteins selected at 9 months** |  |  |  |  |  |  |  |
| **BMP6** |  |  |  |  |  |  |  |
| Control | -0.006 | -0.06 to 0.05 | 0.82 |  | -0.02 | -0.09 to 0.05 | 0.56 |
| Spironolactone | -0.07 | -0.12 to -0.01 | 0.013 |  | -0.07 | -0.14 to -0.001 | 0.046 |
| **CCL16** |  |  |  |  |  |  |  |
| Control | -0.02 | -0.07 to 0.02 | 0.29 |  | -0.02 | -0.07 to 0.03 | 0.48 |
| Spironolactone | 0.03 | -0.02 to 0.08 | 0.30 |  | 0.09 | 0.03 to 0.15 | 0.002 |
| **CSTB** |  |  |  |  |  |  |  |
| Control | -0.04 | -0.09 to 0.02 | 0.19 |  | 0.02 | -0.06 to 0.09 | 0.67 |
| Spironolactone | 0.02 | -0.03 to 0.08 | 0.42 |  | 0.06 | -0.01 to 0.13 | 0.12 |
| **EGFR** |  |  |  |  |  |  |  |
| Control | -0.02 | -0.06 to 0.01 | 0.24 |  | -0.02 | -0.06 to 0.02 | 0.28 |
| Spironolactone | -0.04 | -0.08 to -0.002 | 0.038 |  | -0.05 | -0.09 to -0.01 | 0.011 |
| **FCGR2B** |  |  |  |  |  |  |  |
| Control | -0.02 | -0.07 to 0.03 | 0.41 |  | 0.03 | -0.03 to 0.09 | 0.34 |
| Spironolactone | -0.05 | -0.09 to -0.01 | 0.019 |  | -0.01 | -0.06 to 0.03 | 0.58 |
| **FS** |  |  |  |  |  |  |  |
| Control | -0.02 | -0.07 to 0.03 | 0.50 |  | 0.01 | -0.05 to 0.08 | 0.66 |
| Spironolactone | -0.02 | -0.07 to 0.03 | 0.37 |  | 0.03 | -0.03 to 0.09 | 0.33 |
| **GDF15** |  |  |  |  |  |  |  |
| Control | 0.05 | 0.02 to 0.08 | 0.004 |  | 0.10 | 0.05 to 0.15 | <0.001 |
| Spironolactone | 0.04 | 0.007 to 0.07 | 0.017 |  | 0.13 | 0.09 to 0.18 | <0.001 |
| **GDF2** |  |  |  |  |  |  |  |
| Control | -0.02 | -0.06 to 0.03 | 0.46 |  | -0.01 | -0.06 to 0.04 | 0.66 |
| Spironolactone | -0.02 | -0.07 to 0.02 | 0.33 |  | -0.01 | -0.06 to 0.04 | 0.64 |
| **IDUA** |  |  |  |  |  |  |  |
| Control | -0.03 | -0.07 to 0.02 | 0.25 |  | -0.007 | -0.06 to 0.05 | 0.79 |
| Spironolactone | 0.005 | -0.04 to 0.05 | 0.81 |  | 0.003 | -0.05 to 0.06 | 0.90 |
| **IGFBP1** |  |  |  |  |  |  |  |
| Control | -0.02 | -0.12 to 0.08 | 0.70 |  | 0.08 | -0.05 to 0.22 | 0.23 |
| Spironolactone | -0.11 | -0.21 to -0.01 | 0.026 |  | 0.02 | -0.11 to 0.15 | 0.77 |
| **IL17C** |  |  |  |  |  |  |  |
| Control | -0.03 | -0.12 to 0.06 | 0.53 |  | 0.03 | -0.07 to 0.14 | 0.53 |
| Spironolactone | 0.07 | -0.02 to 0.16 | 0.11 |  | 0.06 | -0.04 to 0.15 | 0.23 |
| **KLK6** |  |  |  |  |  |  |  |
| Control | -0.04 | -0.10 to 0.02 | 0.24 |  | -0.04 | -0.10 to 0.02 | 0.19 |
| Spironolactone | -0.01 | -0.07 to 0.05 | 0.72 |  | 0.03 | -0.03 to 0.09 | 0.32 |
| **MCP1** |  |  |  |  |  |  |  |
| Control | 0.02 | -0.02 to 0.06 | 0.40 |  | 0.03 | -0.02 to 0.09 | 0.24 |
| Spironolactone | 0.02 | -0.02 to 0.07 | 0.30 |  | 0.03 | -0.03 to 0.09 | 0.29 |
| **MMP2** |  |  |  |  |  |  |  |
| Control | -0.03 | -0.08 to 0.02 | 0.19 |  | 0.005 | -0.05 to 0.06 | 0.85 |
| Spironolactone | -0.13 | -0.18 to -0.08 | <0.001 |  | -0.10 | -0.15 to -0.05 | <0.001 |
| **PLAU** |  |  |  |  |  |  |  |
| Control | 0.007 | -0.03 to 0.05 | 0.72 |  | 0.02 | -0.03 to 0.05 | 0.47 |
| Spironolactone | 0.05 | 0.01 to 0.08 | 0.010 |  | 0.04 | -0.003 to 0.08 | 0.07 |
| **SERPINA12** |  |  |  |  |  |  |  |
| Control | 0.01 | -0.02 to 0.05 | 0.44 |  | -0.004 | -0.04 to 0.04 | 0.86 |
| Spironolactone | -0.03 | -0.07 to 0.02 | 0.25 |  | 0.02 | -0.03 to 0.06 | 0.51 |

Estimated marginal means represent the adjusted mean difference versus baseline, derived from linear mixed-effects models accounting for treatment group, baseline protein levels, and eGFR fold change. CI, confidence interval. The remaining abbreviations as in table S1

**Supplemental table 5. Associations of GAL9 and THBS2 slopes with longitudinal echocardiographic trajectories**

|  | THBS2 | |  | GAL9 | | Omnibus P-value |
| --- | --- | --- | --- | --- | --- | --- |
|  | Estimate | 95% CI |  | Estimate | 95% CI |  |
| LVMI | 0.06 | -1.06 to 1.19 |  | -1.30 | -2.66 to 0.07 | 0.174 |
| LVEDVi | -0.42 | -1.40 to 0.57 |  | 0.32 | -0.90 to 1.55 | 0.287 |
| LVEF | 0.11 | -0.05 to 0.27 |  | -0.03 | -0.23 to 0.17 | 0.265 |
| E:e’ | 0.39 | -0.94 to 1.71 |  | 0.54 | -1.20 to 2.28 | 0.520 |
| TAPSE | -1.12 | -5.89 to 3.65 |  | 2.12 | -4.89 to 9.12 | 0.735 |

Abbreviations as in table S3

**Supplemental table 6. Baseline protein expression by quartiles of the PICP fold-change in patients from the Aldo-DHF trial**

|  | PICP fold-change quartiles | | | | P for trend |
| --- | --- | --- | --- | --- | --- |
|  | Q1 (n=92) | Q2 (n=92) | Q3 (n=92) | Q4 (n=92) |  |
| ACE2 | 4.3 (3.9-4.9) | 4.3 (3.9-4.8) | 4.3 (3.8-4.6) | 4.2 (3.8-4.6) | 0.432 |
| ADAMTS13 | 7.8 (7.7-7.8) | 7.8 (7.7-7.9) | 7.8 (7.7-7.9) | 7.8 (7.7-7.9) | 0.629 |
| ADM | 7.9 (7.6-8.1) | 7.9 (7.5-8.2) | 7.9 (7.5-8.2) | 7.9 (7.5-8.2) | 0.942 |
| AGRP | 6.2 (5.9-6.5) | 6.2 (6.0-6.4) | 6.2 (6.0-6.4) | 6.2 (6.0-6.5) | 0.643 |
| AMBP | 8.0 (7.9-8.1) | 8.0 (7.9-8.2) | 8.0 (7.9-8.1) | 8.0 (7.9-8.1) | 0.899 |
| ANG1 | 7.0 (6.6-7.6) | 7.0 (6.5-7.5) | 7.0 (6.5-7.7) | 7.0 (6.6-7.8) | 0.678 |
| BMP6 | 3.9 (3.5-4.1) | 3.8 (3.5-4.1) | 3.7 (3.3-4.1) | 3.7 (3.2-3.9) | 0.013 |
| BNP | 2.5 (1.4-3.6) | 2.0 (1.3-3.0) | 2.2 (1.5-3.4) | 2.0 (1.4-3.2) | 0.475 |
| BOC | 5.0 (4.9-5.2) | 5.0 (4.8-5.1) | 5.0 (4.8-5.1) | 4.9 (4.8-5.1) | <0.001 |
| CA5A | 2.9 (2.5-3.5) | 2.9 (2.3-3.9) | 3.0 (2.3-3.6) | 2.6 (2.2-3.4) | 0.183 |
| CCL17 | 8.5 (8.1-9.0) | 8.5 (8.1-9.0) | 8.6 (8.2-9.0) | 8.6 (8.1-9.0) | 0.273 |
| CCL3 | 7.2 (6.8-7.6) | 7.2 (6.9-7.6) | 7.2 (6.9-7.5) | 7.2 (6.9-7.6) | 0.838 |
| CD4 | 5.7 (5.5-5.9) | 5.7 (5.5-5.9) | 5.6 (5.4-5.9) | 5.7 (5.5-5.8) | 0.12 |
| CD40L | 4.2 (3.7-4.7) | 4.1 (3.6-4.7) | 4.2 (3.9-4.9) | 4.1 (3.6-4.7) | 0.941 |
| CD84 | 5.2 (5.0-5.5) | 5.2 (5.0-5.4) | 5.3 (5.0-5.6) | 5.2 (5.0-5.5) | 0.438 |
| CEACAM8 | 4.8 (4.5-5.2) | 4.9 (4.6-5.4) | 4.9 (4.5-5.5) | 5.0 (4.7-5.3) | 0.056 |
| CTRC | 10.8 (10.3-11.3) | 11.0 (10.5-11.3) | 10.8 (10.4-11.3) | 10.9 (10.5-11.3) | 0.572 |
| CTSL1 | 7.4 (7.2-7.7) | 7.4 (7.2-7.7) | 7.5 (7.3-7.7) | 7.5 (7.3-7.7) | 0.447 |
| CXCL1 | 8.7 (8.3-9.2) | 8.7 (8.3-9.2) | 8.7 (8.1-9.3) | 8.7 (8.3-9.3) | 0.995 |
| DCN | 5.4 (5.3-5.6) | 5.4 (5.3-5.6) | 5.4 (5.3-5.5) | 5.4 (5.3-5.5) | 0.566 |
| DECR1 | 4.6 (4.0-5.2) | 4.6 (3.9-5.2) | 4.8 (4.1-5.5) | 4.3 (3.7-4.9) | 0.111 |
| Dkk1 | 8.1 (7.7-8.4) | 8.1 (7.8-8.3) | 7.9 (7.8-8.4) | 8.1 (7.8-8.3) | 0.703 |
| FABP2 | 8.9 (8.4-9.3) | 9.2 (8.5-9.7) | 9.0 (8.4-9.4) | 8.7 (8.4-9.3) | 0.304 |
| FGF21 | 7.7 (7.0-9.2) | 7.9 (7.1-8.9) | 7.7 (6.9-8.5) | 7.6 (6.8-8.7) | 0.166 |
| FGF23 | 3.8 (3.5-4.2) | 3.8 (3.6-4.4) | 3.8 (3.5-4.1) | 3.9 (3.5-4.4) | 0.498 |
| FS | 9.7 (9.3-10.1) | 9.7 (9.4-10.1) | 9.7 (9.3-10.2) | 9.8 (9.5-10.1) | 0.621 |
| Gal9 | 8.5 (8.3-8.7) | 8.5 (8.3-8.7) | 8.6 (8.3-8.7) | 8.5 (8.3-8.7) | 0.816 |
| GDF2 | 8.2 (8.0-8.4) | 8.3 (8.0-8.5) | 8.2 (7.9-8.4) | 8.2 (7.9-8.4) | 0.55 |
| GH | 8.6 (7.5-10.4) | 8.7 (7.3-10.1) | 8.7 (7.0-10.2) | 8.6 (7.0-10.2) | 0.63 |
| GIF | 8.7 (8.3-9.5) | 9.0 (8.3-9.6) | 8.8 (8.2-9.4) | 8.8 (8.2-9.3) | 0.46 |
| GLO1 | 7.3 (7.0-7.7) | 7.3 (6.9-7.7) | 7.4 (7.0-7.7) | 7.3 (6.9-7.8) | 0.331 |
| GT | 2.1 (1.8-2.5) | 2.3 (2.0-2.7) | 2.2 (1.8-2.5) | 2.1 (1.8-2.5) | 0.439 |
| HAOX1 | 6.0 (5.0-6.8) | 5.5 (4.8-6.5) | 6.0 (5.1-6.8) | 5.6 (4.8-6.4) | 0.271 |
| HBEGF | 4.6 (4.4-4.7) | 4.6 (4.5-4.7) | 4.5 (4.4-4.7) | 4.5 (4.4-4.7) | 0.25 |
| HO1 | 12.5 (12.2-12.7) | 12.5 (12.2-12.7) | 12.5 (12.2-12.8) | 12.5 (12.2-12.7) | 0.907 |
| hOSCAR | 11.8 (11.7-11.9) | 11.8 (11.6-11.9) | 11.8 (11.6-12.0) | 11.8 (11.6-11.9) | 0.914 |
| HSP27 | 9.5 (9.1-9.9) | 9.4 (9.1-9.8) | 9.6 (9.3-10.1) | 9.5 (9.0-10.0) | 0.581 |
| IDUA | 6.3 (5.9-6.7) | 6.3 (6.0-6.6) | 6.3 (6.0-6.6) | 6.3 (6.0-6.6) | 0.472 |
| IgGFcreceptorIIb | 4.5 (3.7-5.1) | 4.4 (3.5-5.0) | 4.5 (3.8-5.0) | 4.5 (3.6-4.9) | 0.725 |
| IL16 | 7.6 (7.4-7.9) | 7.5 (7.2-7.8) | 7.6 (7.3-7.9) | 7.5 (7.2-7.8) | 0.695 |
| IL17D | 3.4 (3.2-3.6) | 3.4 (3.1-3.5) | 3.3 (3.1-3.5) | 3.3 (3.1-3.6) | 0.19 |
| IL18 | 8.9 (8.6-9.3) | 8.8 (8.4-9.2) | 8.9 (8.4-9.4) | 8.8 (8.5-9.1) | 0.271 |
| IL1ra | 5.7 (5.4-6.5) | 5.8 (5.4-6.4) | 6.0 (5.4-6.5) | 5.9 (5.6-6.4) | 0.284 |
| IL1RL2 | 5.6 (5.4-6.0) | 5.6 (5.3-5.9) | 5.6 (5.2-5.8) | 5.6 (5.3-5.9) | 0.298 |
| IL27 | 6.5 (6.2-6.6) | 6.5 (6.2-6.6) | 6.4 (6.2-6.6) | 6.5 (6.2-6.6) | 0.78 |
| IL4RA | 2.5 (2.3-2.7) | 2.5 (2.4-2.7) | 2.5 (2.3-2.8) | 2.5 (2.3-2.7) | 0.914 |
| IL6 | 4.3 (4.0-4.8) | 4.3 (4.0-4.9) | 4.5 (4.1-4.9) | 4.4 (3.8-5.1) | 0.742 |
| ITGB1BP2 | 2.3 (1.9-2.8) | 2.2 (1.7-2.8) | 2.5 (2.1-3.2) | 2.3 (1.8-2.9) | 0.246 |
| KIM1 | 8.9 (8.5-9.6) | 9.0 (8.5-9.6) | 8.8 (8.5-9.5) | 8.8 (8.3-9.6) | 0.255 |
| LEP | 8.1 (7.3-8.7) | 8.0 (7.4-8.6) | 7.9 (7.2-8.9) | 8.0 (7.3-8.7) | 0.998 |
| LOX1 | 6.8 (6.5-7.5) | 6.9 (6.6-7.5) | 6.9 (6.6-7.7) | 7.0 (6.6-7.6) | 0.257 |
| LPL | 10.4 (9.9-10.5) | 10.4 (10.1-10.6) | 10.2 (9.9-10.5) | 10.3 (9.8-10.5) | 0.06 |
| MARCO | 7.6 (7.4-7.7) | 7.5 (7.4-7.7) | 7.5 (7.4-7.7) | 7.5 (7.4-7.7) | 0.604 |
| MERTK | 7.0 (6.7-7.2) | 6.9 (6.8-7.2) | 6.8 (6.6-7.1) | 6.9 (6.8-7.2) | 0.64 |
| MMP12 | 8.2 (7.6-8.5) | 8.1 (7.6-8.7) | 8.2 (7.7-8.6) | 8.1 (7.4-8.6) | 0.759 |
| MMP7 | 11.5 (11.4-11.7) | 11.6 (11.4-11.8) | 11.5 (11.3-11.7) | 11.5 (11.3-11.7) | 0.373 |
| NEMO | 4.3 (4.0-4.9) | 4.2 (4.0-4.7) | 4.5 (4.1-5.0) | 4.4 (4.0-4.9) | 0.414 |
| PAPPA | 1.8 (1.5-2.1) | 1.8 (1.5-2.1) | 1.7 (1.4-2.0) | 1.7 (1.3-2.0) | 0.155 |
| PAR1 | 8.5 (8.3-8.8) | 8.5 (8.3-8.7) | 8.6 (8.3-8.8) | 8.5 (8.3-8.8) | 0.423 |
| PARP1 | 1.4 (1.0-1.7) | 1.3 (1.0-1.6) | 1.4 (1.1-1.7) | 1.4 (1.1-1.7) | 0.376 |
| PDGFsubunitB | 8.5 (8.1-9.3) | 8.6 (8.0-9.2) | 8.6 (7.9-9.3) | 8.8 (8.2-9.5) | 0.16 |
| PDL2 | 4.0 (3.8-4.2) | 4.1 (3.8-4.2) | 4.0 (3.7-4.2) | 4.0 (3.8-4.2) | 0.452 |
| PGF | 9.1 (8.9-9.3) | 9.1 (8.8-9.4) | 9.1 (8.8-9.2) | 9.0 (8.9-9.4) | 0.361 |
| PIgR | 3.3 (3.2-3.4) | 3.4 (3.3-3.5) | 3.3 (3.3-3.4) | 3.3 (3.3-3.4) | 0.601 |
| PRELP | 8.4 (8.2-8.5) | 8.3 (8.2-8.5) | 8.3 (8.2-8.4) | 8.3 (8.2-8.5) | 0.049 |
| PRSS27 | 9.6 (9.3-9.8) | 9.7 (9.4-10.0) | 9.6 (9.3-9.9) | 9.5 (9.3-9.8) | 0.325 |
| PRSS8 | 9.5 (9.3-9.8) | 9.6 (9.3-9.8) | 9.6 (9.3-9.8) | 9.6 (9.3-9.8) | 0.674 |
| PSGL1 | 4.7 (4.6-4.9) | 4.7 (4.5-4.9) | 4.7 (4.5-4.9) | 4.7 (4.6-4.8) | 0.336 |
| PTX3 | 4.2 (4.0-4.6) | 4.2 (4.0-4.5) | 4.3 (4.0-4.5) | 4.3 (4.1-4.5) | 0.479 |
| RAGE | 13.1 (12.8-13.4) | 13.2 (12.9-13.4) | 13.0 (12.7-13.3) | 13.0 (12.8-13.3) | 0.045 |
| REN | 7.8 (7.0-8.7) | 8.2 (7.4-8.9) | 7.8 (7.2-8.5) | 8.1 (7.3-8.6) | 0.707 |
| SCF | 10.3 (10.1-10.5) | 10.4 (10.2-10.6) | 10.2 (10.1-10.4) | 10.3 (10.0-10.5) | 0.166 |
| SERPINA12 | 3.5 (3.0-4.2) | 3.4 (2.9-4.0) | 3.4 (2.8-4.2) | 3.3 (2.6-4.1) | 0.183 |
| SLAMF7 | 4.3 (4.0-4.8) | 4.3 (4.0-4.7) | 4.4 (4.0-4.8) | 4.3 (4.0-4.7) | 0.63 |
| SOD2 | 9.7 (9.6-9.7) | 9.7 (9.6-9.7) | 9.7 (9.6-9.7) | 9.7 (9.6-9.7) | 0.982 |
| SORT1 | 9.1 (9.0-9.3) | 9.1 (9.0-9.3) | 9.2 (9.0-9.3) | 9.1 (9.0-9.3) | 0.807 |
| SPON2 | 9.8 (9.7-9.9) | 9.8 (9.7-9.9) | 9.8 (9.7-9.8) | 9.8 (9.7-9.9) | 0.667 |
| SRC | 6.2 (5.5-6.8) | 5.9 (5.4-6.8) | 6.6 (6.0-7.2) | 6.2 (5.5-6.9) | 0.18 |
| STK4 | 2.5 (1.9-3.3) | 2.3 (1.8-3.0) | 2.7 (2.2-3.7) | 2.5 (1.8-3.4) | 0.171 |
| TF | 6.3 (6.2-6.5) | 6.4 (6.2-6.6) | 6.3 (6.0-6.5) | 6.4 (6.1-6.5) | 0.715 |
| TGM2 | 5.9 (5.5-6.4) | 5.8 (5.5-6.3) | 5.8 (5.5-6.2) | 5.9 (5.6-6.6) | 0.291 |
| THBS2 | 6.0 (5.8-6.1) | 5.9 (5.8-6.1) | 6.0 (5.9-6.1) | 5.9 (5.8-6.0) | 0.025 |
| THPO | 3.0 (2.9-3.2) | 3.1 (2.9-3.3) | 3.0 (2.8-3.2) | 3.1 (2.9-3.3) | 0.862 |
| TIE2 | 8.1 (8.0-8.3) | 8.1 (8.0-8.2) | 8.1 (8.0-8.3) | 8.1 (8.0-8.3) | 0.554 |
| TM | 11.2 (11.0-11.4) | 11.1 (11.0-11.4) | 11.1 (10.9-11.4) | 11.1 (10.9-11.3) | 0.019 |
| TNFRSF10c | 3.8 (3.6-4.1) | 3.8 (3.6-4.0) | 3.8 (3.5-4.0) | 3.8 (3.6-4.1) | 0.852 |
| TNFRSF11a | 6.3 (6.0-6.6) | 6.3 (6.0-6.6) | 6.3 (6.0-6.6) | 6.3 (6.0-6.6) | 0.807 |
| TNFRSF13b | 9.3 (9.1-9.5) | 9.2 (8.9-9.4) | 9.2 (9.1-9.5) | 9.2 (9.0-9.5) | 0.284 |
| TRAILR2 | 6.4 (6.2-6.7) | 6.5 (6.2-6.8) | 6.5 (6.2-6.7) | 6.5 (6.2-6.7) | 0.898 |
| VEGFD | 8.4 (8.1-8.5) | 8.3 (8.1-8.5) | 8.3 (8.1-8.5) | 8.3 (8.1-8.6) | 0.928 |
| VSIG2 | 4.8 (4.4-5.1) | 4.8 (4.5-5.2) | 4.7 (4.5-5.2) | 4.7 (4.5-5.1) | 0.549 |
| XCL1 | 5.6 (5.3-6.0) | 5.7 (5.3-6.0) | 5.7 (5.4-6.1) | 5.6 (5.3-6.1) | 0.587 |

Abreviations as in table S1. Values are expressed as median (interquartile range)

**Supplemental table 7. Optimized hyperparameters for the machine learning models**

|  | **Meaning** | **Search range** | **1 month** | **9 months** |
| --- | --- | --- | --- | --- |
| **Artificial Neural Network** |  |  |  |  |
| Hidden Layer 1 |  |  |  |  |
| Units | Number of neurons in the hidden layer | (100, 540) | 540 | 525 |
| L1 regularization | L1 penalty | (1x10^-6^, 1x10^-2^) | 3.5x10^-5^ | 4x10^-4^ |
| L2 regularization | L2 penalty | (1x10^-7^, 1x10^-3^) |  | 2.2x10^-6^ |
| Hidden Layer 2 |  |  |  |  |
| Units | Number of neurons in the hidden layer | (100, 500) |  | 220 |
| L1 regularization | L1 penalty | (1x10^-6^, 1x10^-2^) |  | 9.5x10^-6^ |
| Layer batch normalization |  |  | yes | yes |
| Learning rate | Size of the step for updating the weights | (1x10^-5^, 1x10^-1^) | 1x10^-2^ | 7x10^-3^ |
| Optimizer | Optimization algorithm |  | rmsprop | rmsprop |
| Batch size | Number of training samples | (4,32) | 4 | 4 |
| Nº of epochs | Number of complete passes through the training data set | (200,600) | 400 | 300 |
| **Conditional random Forest** |  |  |  |  |
| Nº of input features (mtry) | Number of features randomly selected for each decision tree | (10,200) | 177 | 142 |
| Nº of trees | Number of decision trees | (500,9000) | 2600 | 6000 |
| **Extreme gradient boosting** |  |  |  |  |
| Nº boosting interations | Number of boosting rounds or trees to build | (1,100) | 7 | 33 |
| Maximum depth | Maximum tree Depth for base learners | (1,15) | 2 | 1 |
| Colsample by tree | Subsample ratio of columns when constructing each tree | (0.01,1) | 0.5 | 0.5 |
| Learning rate (eta) | Boosting learning rate | (0.01,1) | 0.93 | 1 |
| Gamma | Required to further partitioning on a leaf node of the tree | (0,50) | 0 | 0 |
| Minimum child weight | Minimum weight to create a new node in the tree | (0,20) | 0.1 | 14 |
| Subsample | Subsample ratio of the training instance | (0.1,1) | 0.9 | 1 |
| L1 regularization | L1 penalty | (0.01,5) | 0.01 | 0.02 |
| L2 regularization | L2 penalty | (0.01,5) | 1.1 | 0.44 |
| **Elastic net regression** |  |  |  |  |
| Alpha | Weight of the L1 penalty relative to the L2 penalty | (0.01,1) | 0.6 | 0.1 |
| Lambda | Regularization parameter | (0.01,10) | 0.016 | 0.064 |

**
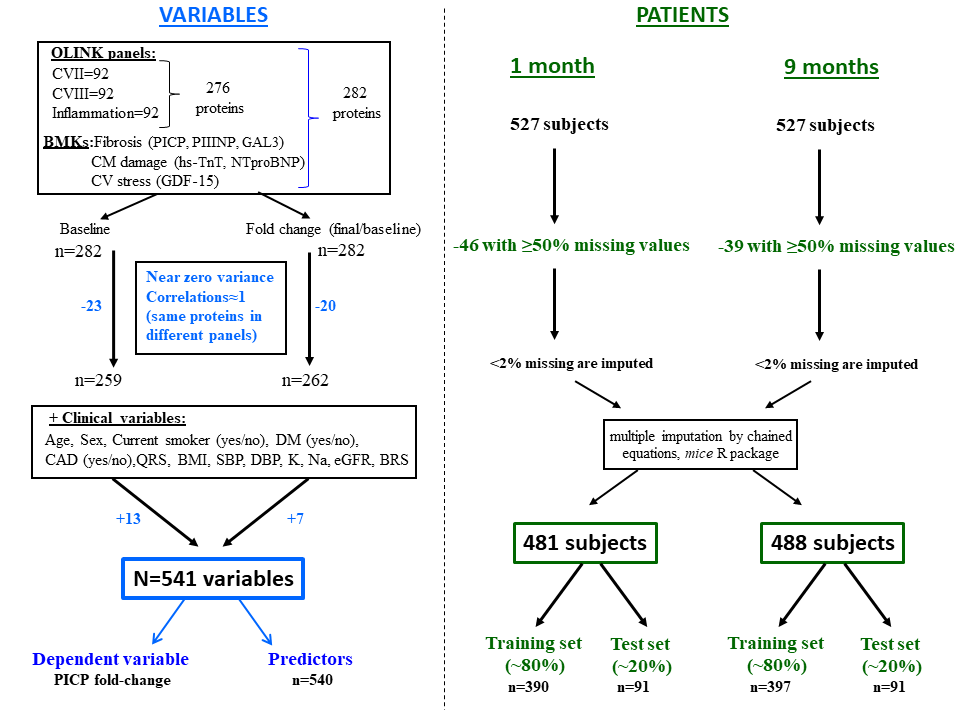
**

**Supplemental Figure 1.** Variables and participants flow diagram. CV means cardiovascular; PICP, procollagen type I C-terminal propeptide; PIIINP, procollagen type III N-terminal propeptide; GDF-15, growth differentiation factor 15; GAL3, galectin 3; NT-proBNP; N-terminal pro-brain natriuretic peptide; Hs-TnT, high sensitivity troponin T; DM, diabetes mellitus; CAD, coronary artery disease; BMI, body mass index; SBP, systolic blood pressure; DBP, diastolic blood pressure; eGFR, estimated glomerular filtration rate; BRS, breathlessness scale.


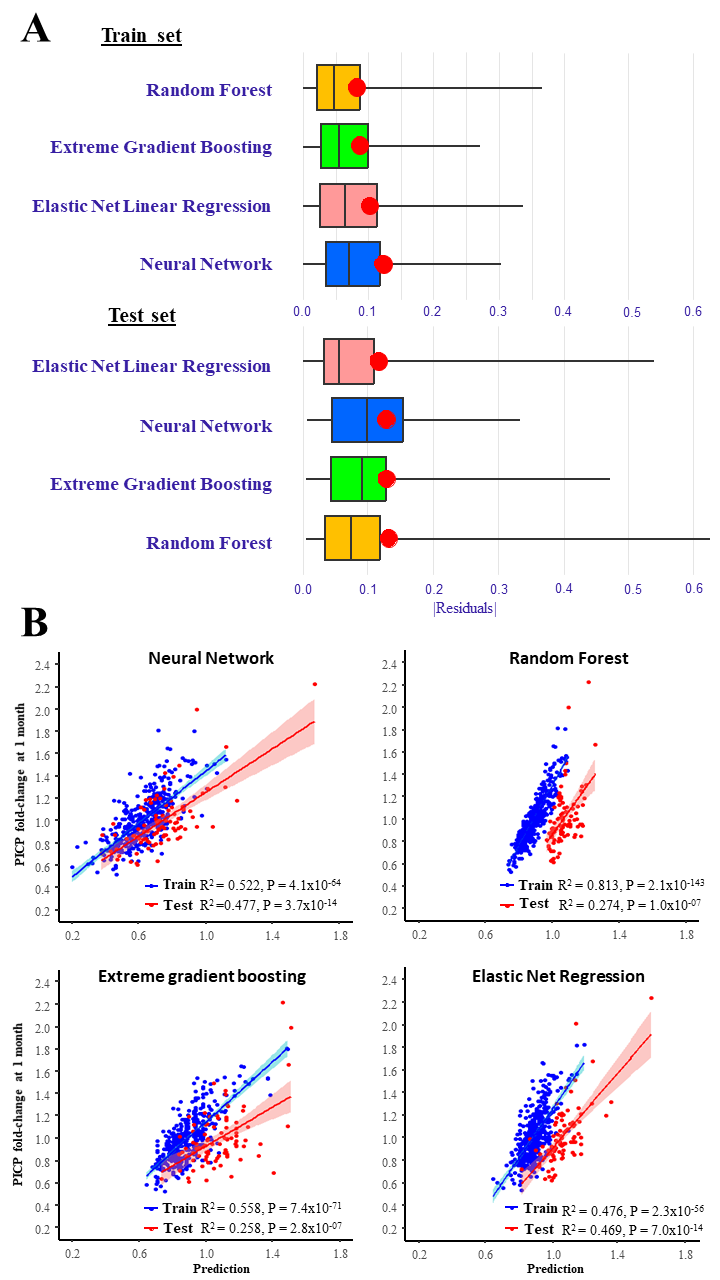


**Supplemental Figure 2**. Machine learning models’ performances at 1 month. Box plots of the residual values (A) and scatter plots with fitted regression lines and 95% confidence intervals (B) for the prediction of the PICP change by each ML model in the train and test sets. The red dots in panel A stand for root mean square of residuals (RMSE).


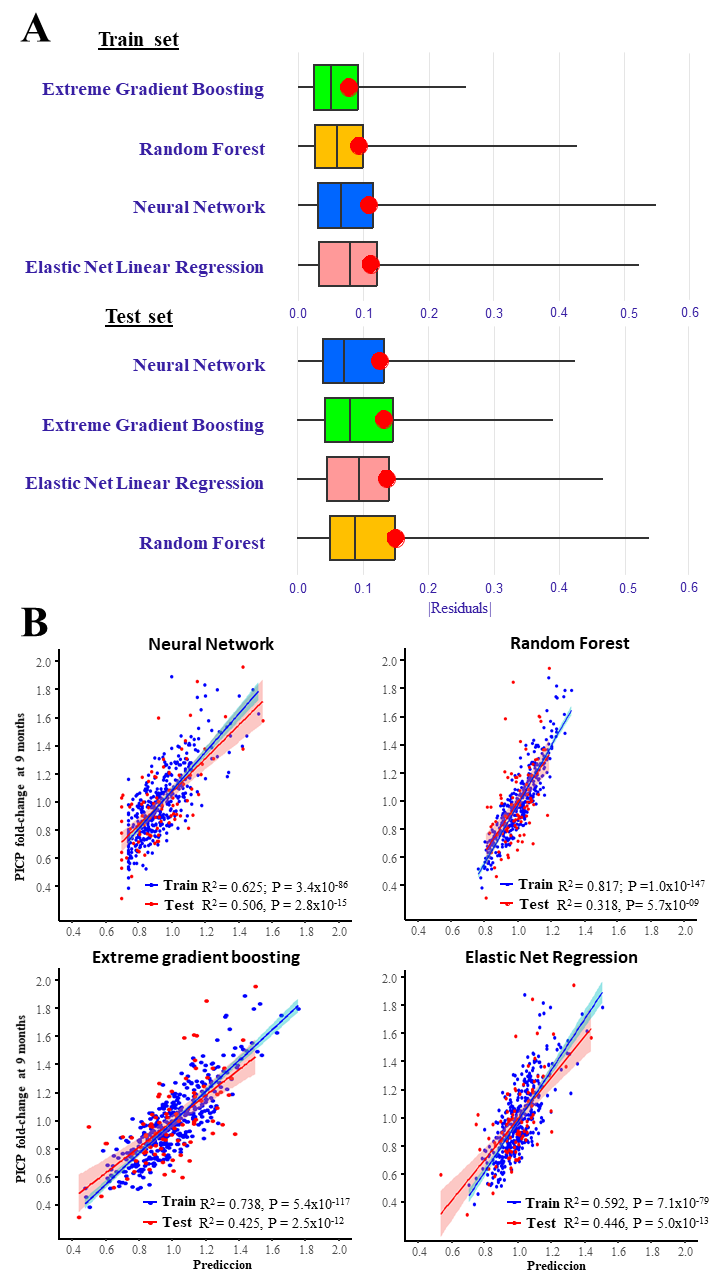


**Supplemental figure 3**. Machine learning (ML) models’ performances at 9 months. Box plots of the residual values (A) and scatter plots with fitted regression lines and 95% confidence intervals (B) for the prediction of the PICP change by each ML model in the train and test sets. The red dots in panel A stand for root mean square of residuals (RMSE).


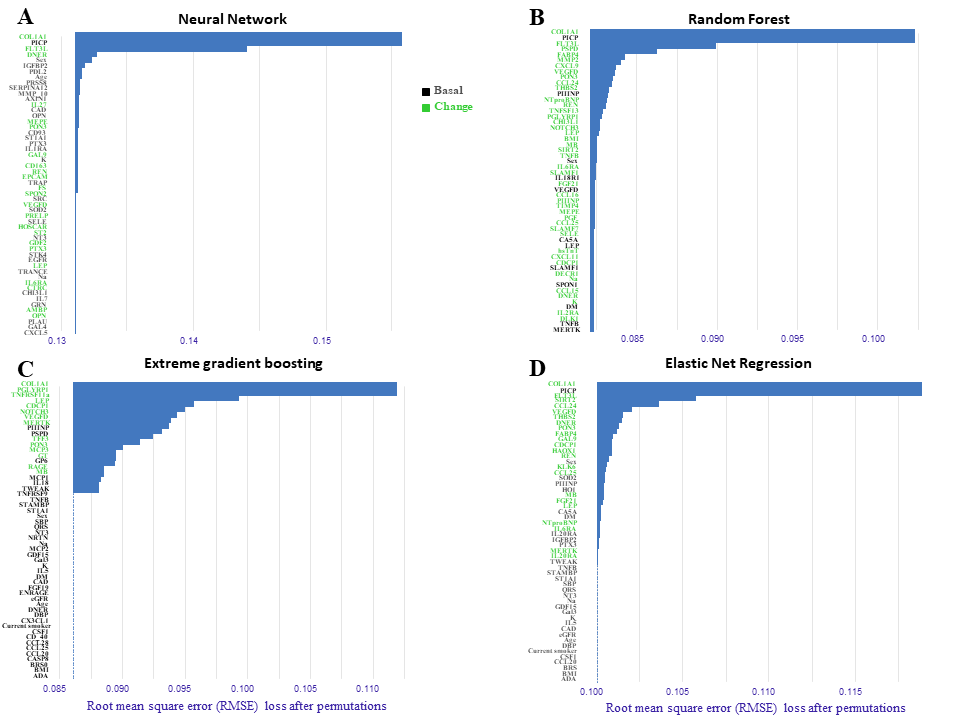


**Supplemental figure 4**. Variable importance charts for baseline (black) and fold-change (green) expression of the 10% more important proteins as selected in the artificial neural network (A), conditional random forest (B), extreme gradient boosting (C) and elastic-net regression (D) models at 1 month. The longer the bar chart, the higher the importance of the feature is in each model.


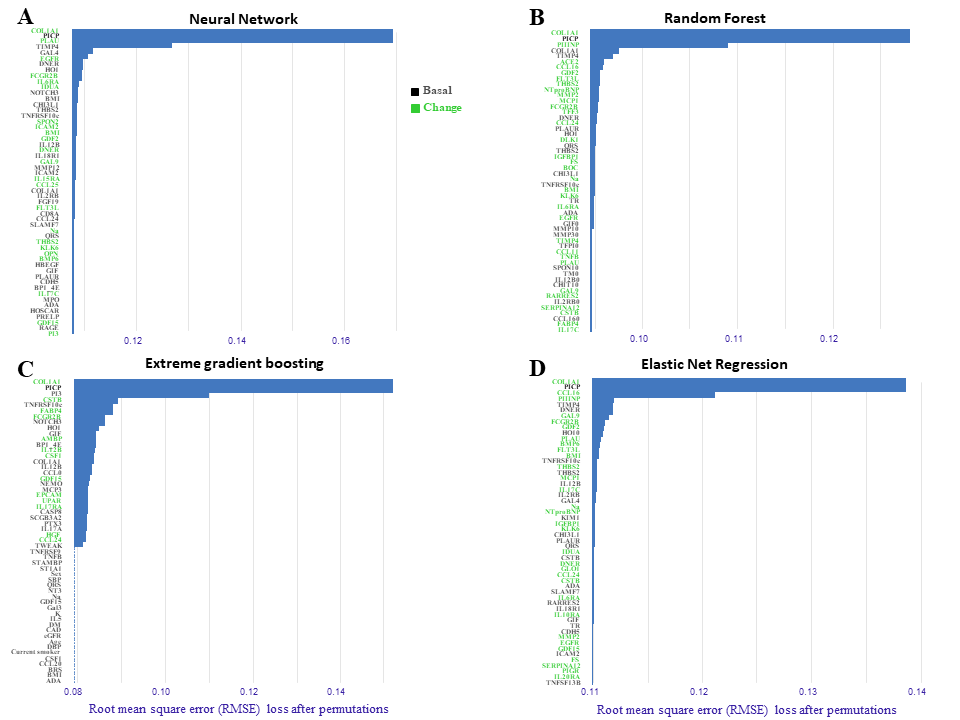


**Supplemental figure 5**. Variable importance charts for baseline (black) and fold-change (green) expression of the 10% more important proteins as selected in the artificial neural network (A), conditional random forest (B), extreme gradient boosting (C) and elastic-net regression (D) models at 9 months. The longer the bar chart, the higher the importance of the feature is in each model.


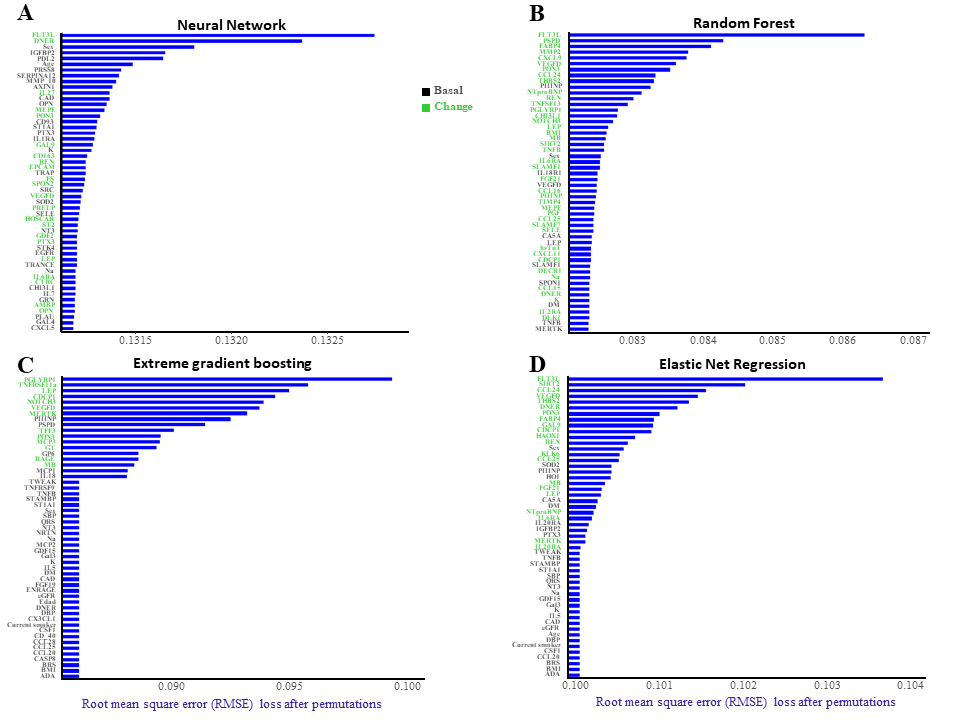


**Supplemental figure 6**. Variable importance charts for baseline (black) and fold-change (green) expression of the 10% more important proteins, except for COL1A1 and baseline PICP, as selected in the artificial neural network (A), conditional random forest (B), extreme gradient boosting (C) and elastic-net regression (D) models at 1 month. The longer the bar chart, the higher the importance of the feature is in each model.


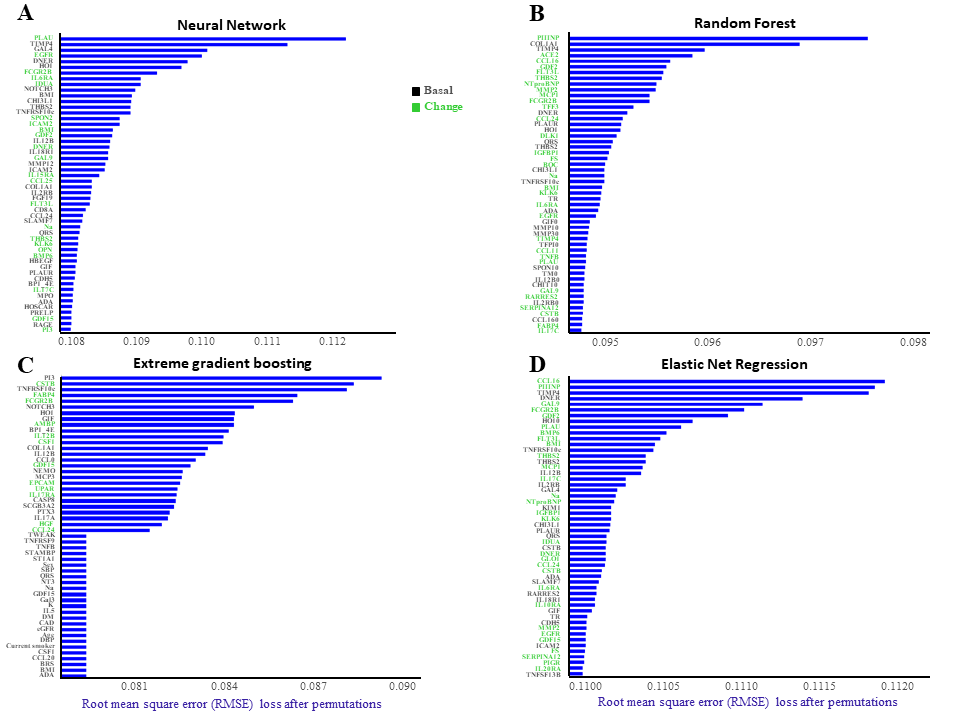


**Supplemental figure 7**. Variable importance charts for baseline (black) and fold-change (green) expression of the 10% more important proteins, except for COL1A1 and baseline PICP, as selected in the artificial neural network (A), conditional random forest (B), extreme gradient boosting (C) and elastic-net regression (D) models at 9 months. The longer the bar chart, the higher the importance of the feature is in each model.


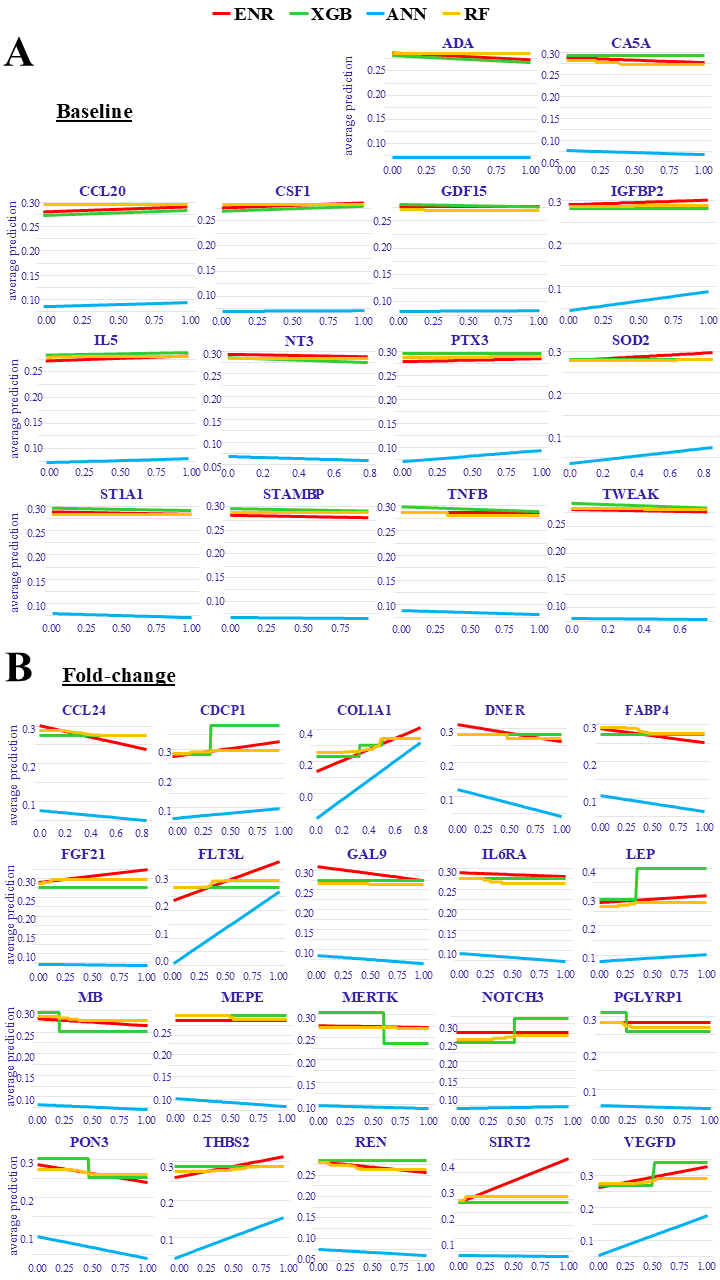


**Supplemental figure 8.** Partial dependence profiles of the machine learning (ML) PICP fold-change predictions at 1 month, as functions of the ML-selected proteins according to the elastic net regression (ENR, red line), extreme gradient boosting (XGB, green line), artificial neural network (ANN, blue line) and conditional random forest (RF, yellow line) algorithms.


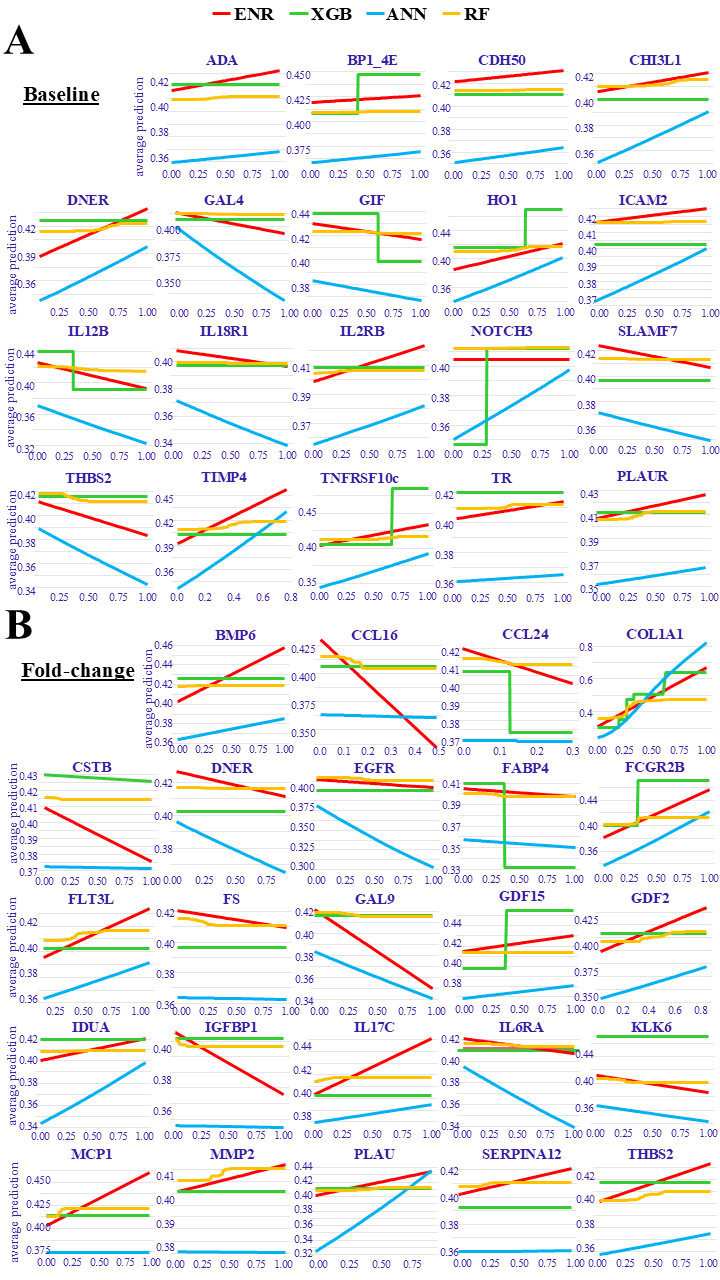


**Supplemental figure 9.** Partial dependence profiles of the machine learning (ML) PICP changes prediction at 9 months, as functions of the ML-selected proteins according to the elastic net regression (ENR, red line), extreme gradient boosting (XGB, green line), artificial neural network (ANN, blue line) and conditional random forest (RF, yellow line) algorithms.


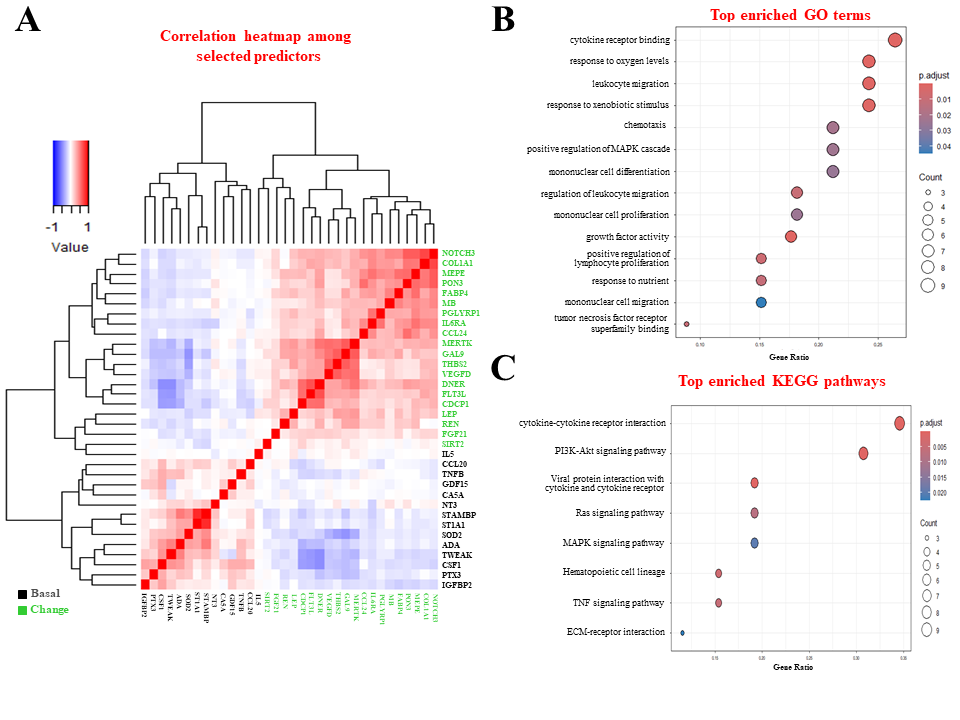


**Supplemental figure 10.** Correlations, GO and KEGG terms for the ML-selected proteins associated with PICP fold-changes at 1 month. A) Heatmap plot showing the correlations (pearson) among the baseline levels (black) and fold-changes (green) of the ML-selected proteins. Red, positively related; blue, negatively related; and white, nonrelated. Dot plots depict significantly enriched GO terms (B) and KEGG pathways (C) based on the background of the ML-selected proteins at 1 month, showing p-value scores (coloured), gene ratio and count (dot size). GO, gene ontology; KEGG, Kyoto encyclopedia genes and genomes; ML, machine learning.


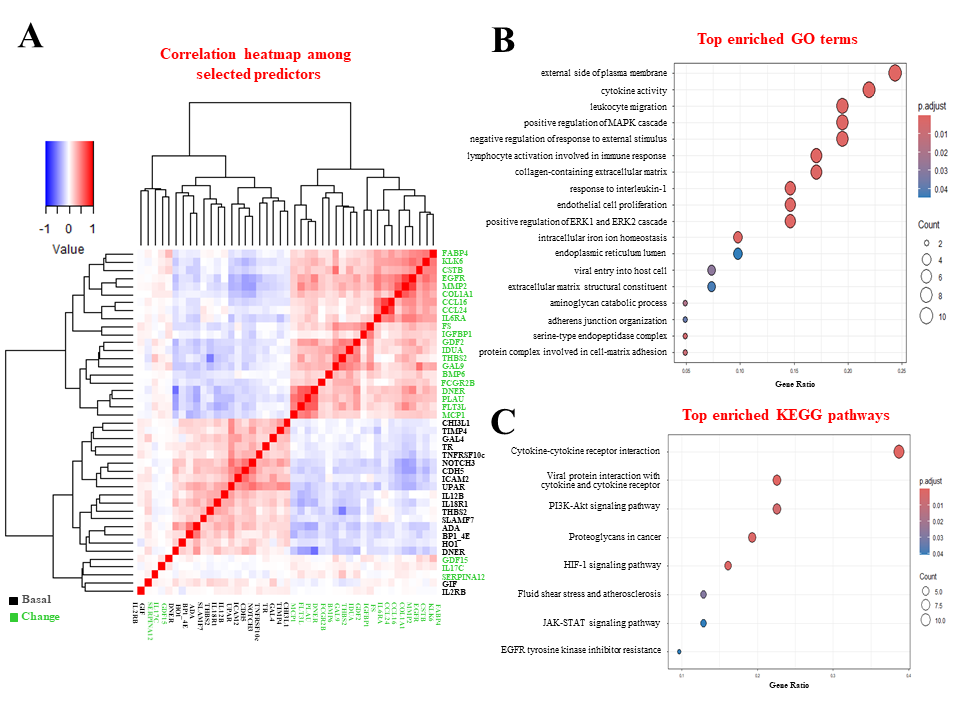


**Supplemental figure 11.** Correlations, GO and KEGG terms for the ML-selected proteins associated with PICP fold-changes at 9 months. A) Heatmap plot showing the correlations (pearson) among the baseline levels (black) and fold-changes (green) of the ML-selected proteins. Red, positively related; blue, negatively related; and white, nonrelated. Dot plots depicts significantly enriched GO terms (B) and KEGG pathways (C) based on the background of the ML-selected proteins at 1 month, showing p-value scores (colored), gene ratio and count (dot size). GO, gene ontology; KEGG, Kyoto encyclopedia genes and genomes; ML, machine learning.


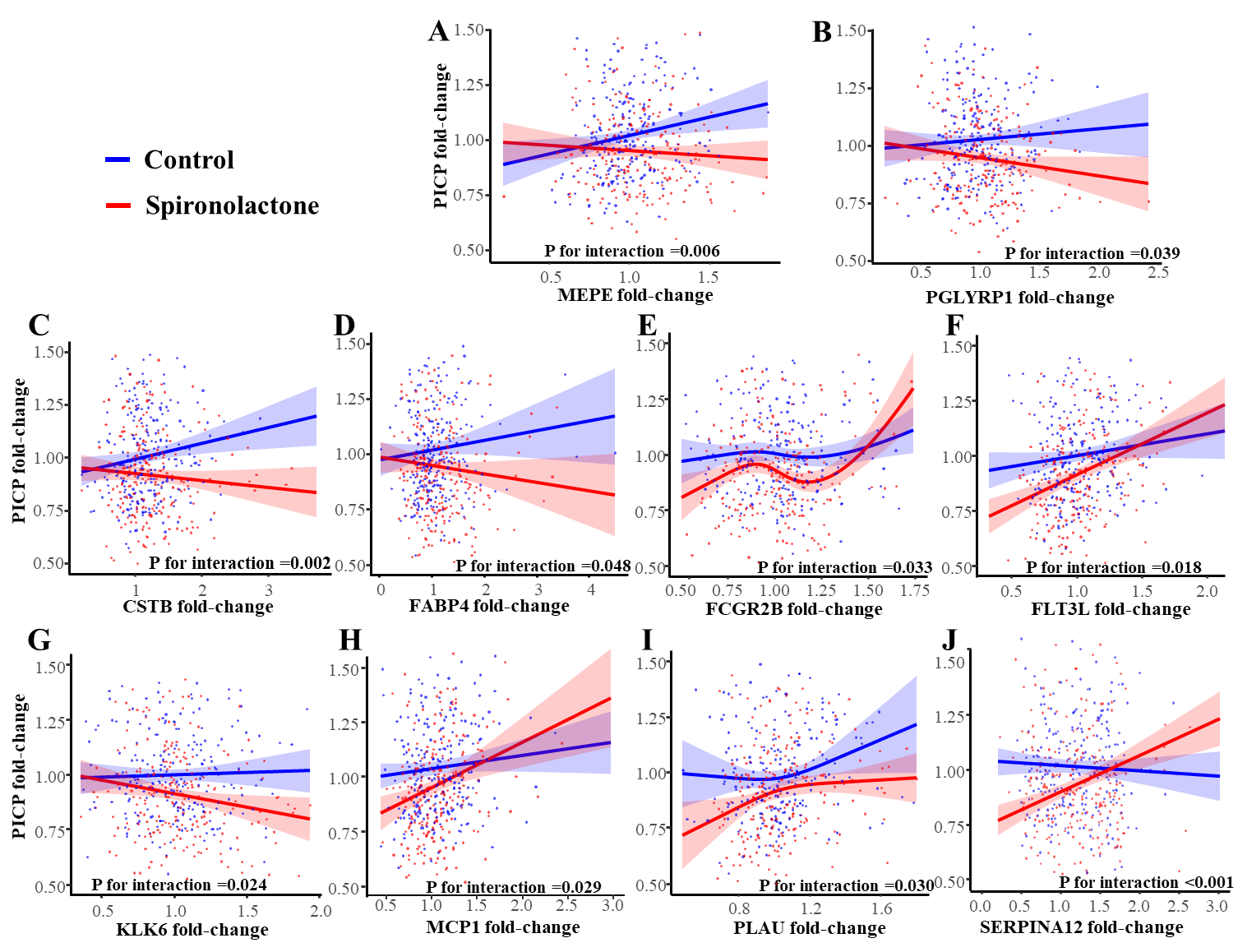


**Supplemental figure 12.** Regression lines showing PICP fold-change (final/baseline values) at 1 month as a function of MEPE (A) and PGLYRP1 fold-changes (2^final/2^baseline NPX) (B), and PICP fold-change at 9 months as a function of CSTB (C), FABP4 (D), FCGR2B (E), FLT3L (F), KLK6 (G), MCP1 (H), PLAU (I) and SERPINA12 (J) fold-changes for control (blue) and spironolactone(red)-treated patients after linear regression analyses including an interaction term between each protein and the randomization variable, and adjusted by baseline values of PICP, the respective protein and eGFR changes. Color areas show the 95% confidence intervals. FCGR2B and PLAU were modeled with restricted cubic splines with 4 and 3 knots, respectively.


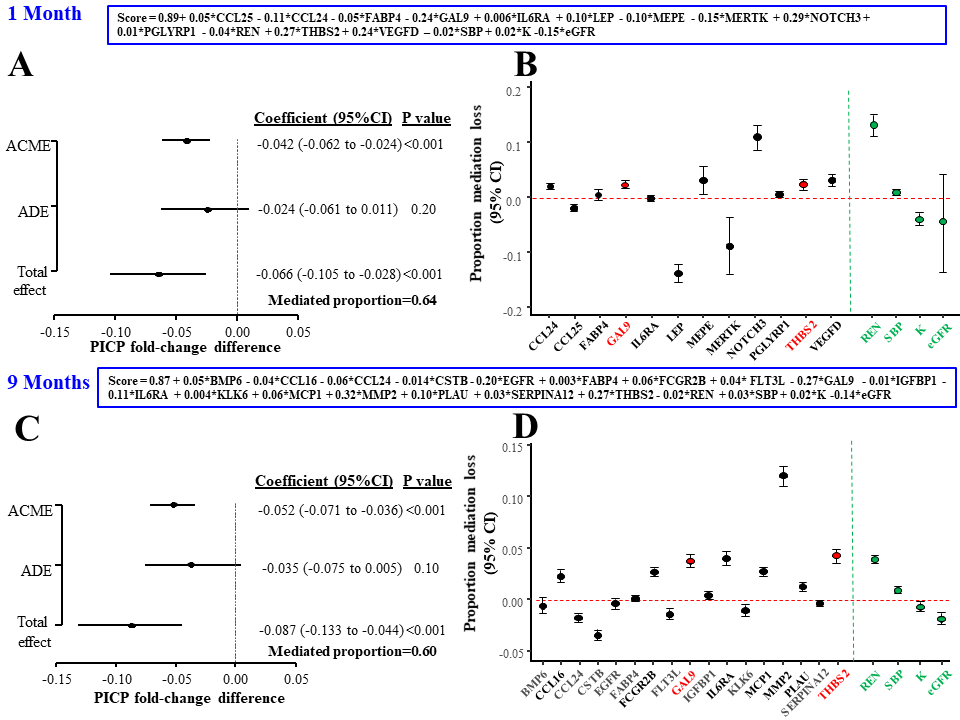


**Supplemental figure 13.** Mediation analyses in HOMAGE clinical trial. Effect decomposition plots for the combination of the selected candidates and clinical variables (highlighted in green) in mediator signature scores derived from linear regression models using PICP fold-change as the continuous target and including all selected variable fold-changes as independent variables at 1 (A) and 9 months (C). Graphs depicting mediation loss after removal of each protein from the mediator scores estimated at 1 and 9 months of spironolactone are shown in panels B and D, respectively. GAL9 and THBS2 are highlighted in red. Estimates are accompanied by 95% percentile bootstrap confidence intervals based on 1000 bootstrap resamples. Variables in red are those with higher importance in the mediation. ACME, average causal mediation effect; ADE, average direct effect.


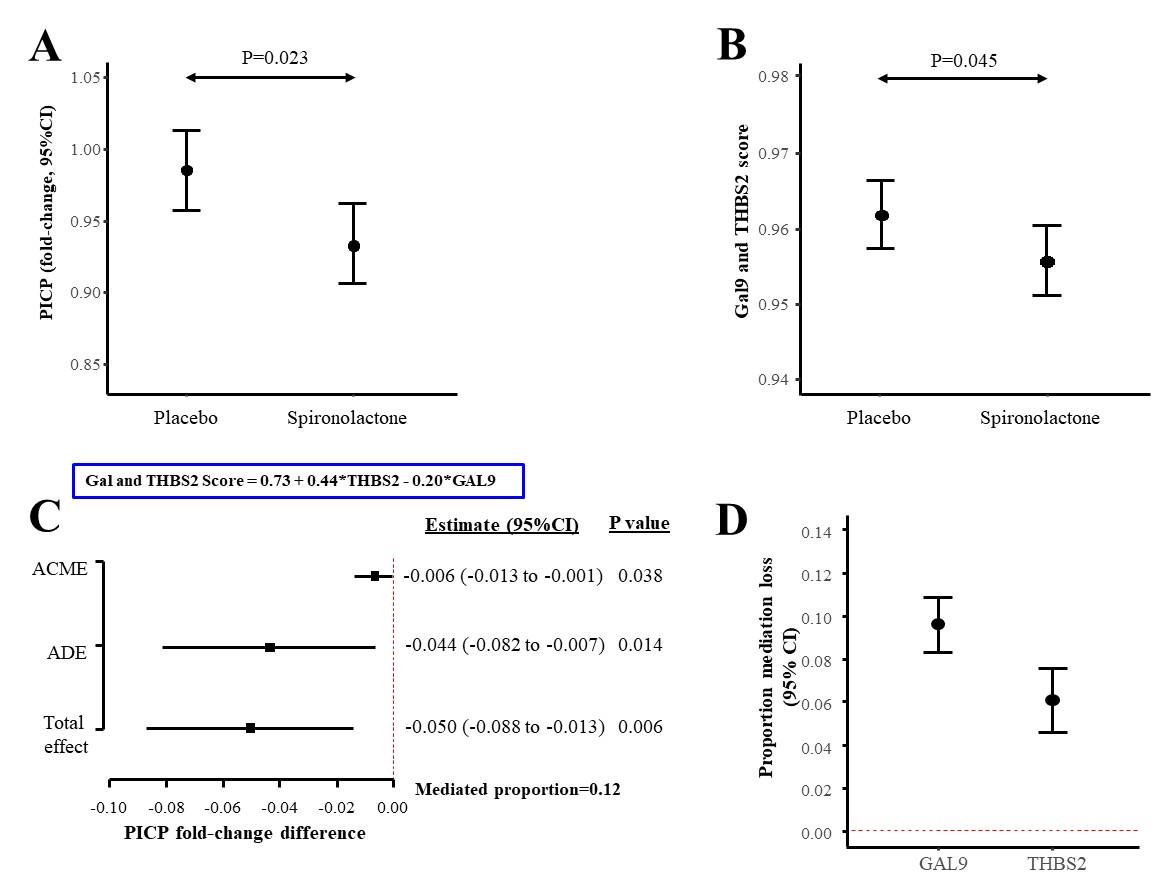


**Supplemental figure 14**. Mediation analyses in Aldo-DHF clinical trial. Panel A depicts predicted mean PICP fold-change values (estimated marginal means by group) and 95% CI intervals in patients treated with placebo or spironolactone after robust linear regression analysis with PICP fold-change as the dependent variable, and the randomization variable and log2-transformed baseline PICP as independent variables. Panel B represents the estimated marginal means (and 95% CI) of the mediator term score derived from a linear model using PICP fold-change as the dependent variable and including THBS2 and GAL9 fold-changes as independent variables in patients treated with placebo or spironolactone. Panel C shows an effect decomposition plot for the THBS2 and GAL9-based score as a mediator term. This score rendered a mediated proportion of 0.12, suggesting that the combined variation in these proteins mediated 12% of the total effect of spironolactone on PICP. Panel D depicts the mediation loss after the removal of each protein from the mediator term. Estimates are accompanied by 95% percentile bootstrap confidence intervals based on 1000 bootstrap resamples. ACME, average causal mediation effect; ADE, average direct effect.

**
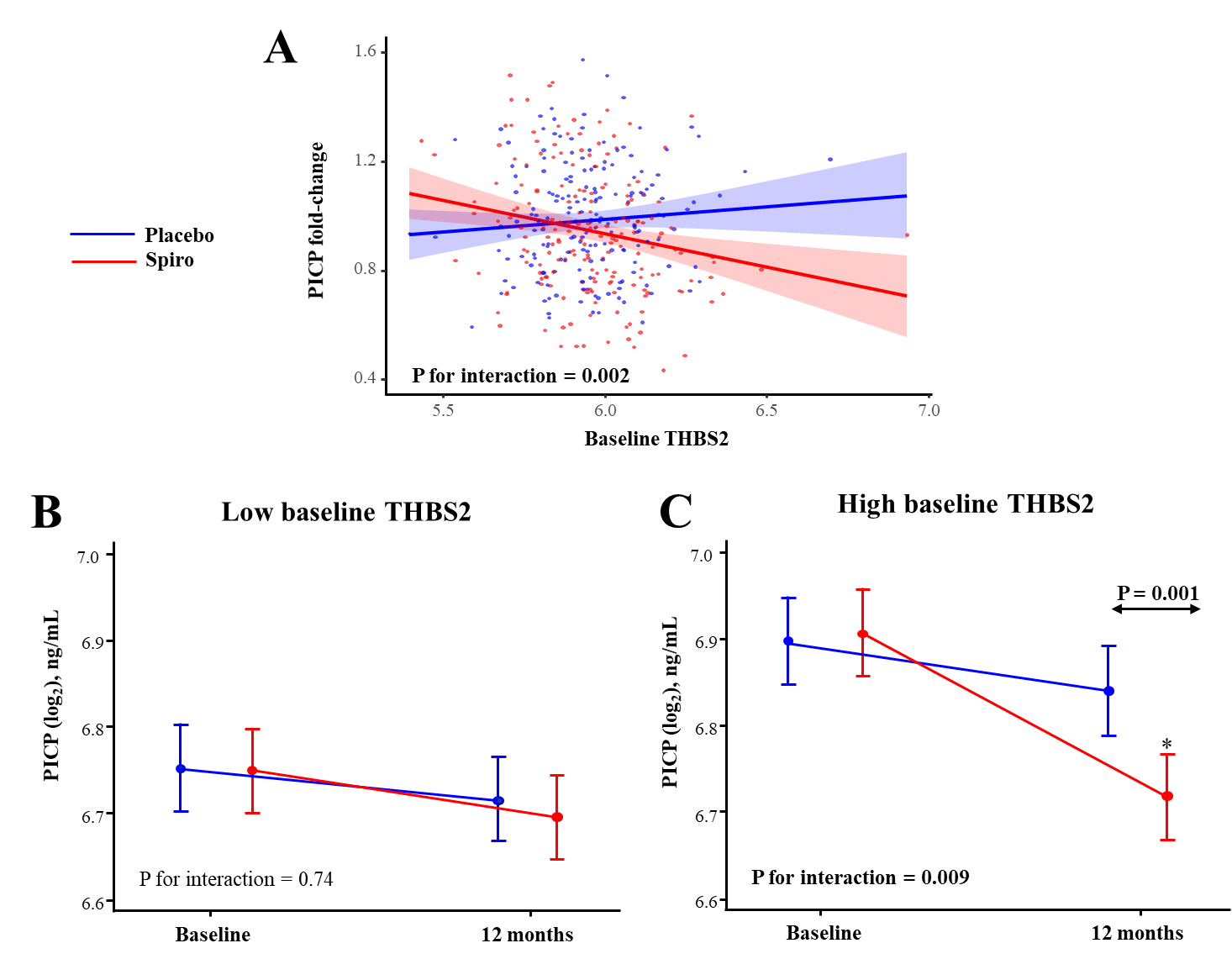
**

**Supplemental figure 15.** Regression lines showing PICP fold-change (final/baseline values) as a function of baseline THBS2 for placebo (blue) and spironolactone(red)-treated patients from the Aldo-DHF trial after linear regression analyses including an interaction term between THBS2 and the randomization variable, and adjusted by baseline values of PICP, and the estimated glomerular filtration rate fold-change. Color areas show the 95% confidence intervals. Graphs B and C show PICP levels according to assigned treatment in patients with low (below the median: NPX<5.95, n=184, 50.5% treated with spironolactone) and high (above the median: NPX≥5.95, n=184, 52.7% treated with spironolactone) THBS2 levels at baseline, respectively. Data are expressed as the predicted mean values (estimated marginal means of visits by group) and 95% CI at baseline and at 12 months in patients treated with spironolactone or placebo after linear mixed regression analysis with PICP as the dependent variable, and treatment, visit, their interaction term, and baseline PICP and estimated glomerular filtration rate fold-change as fixed effects.


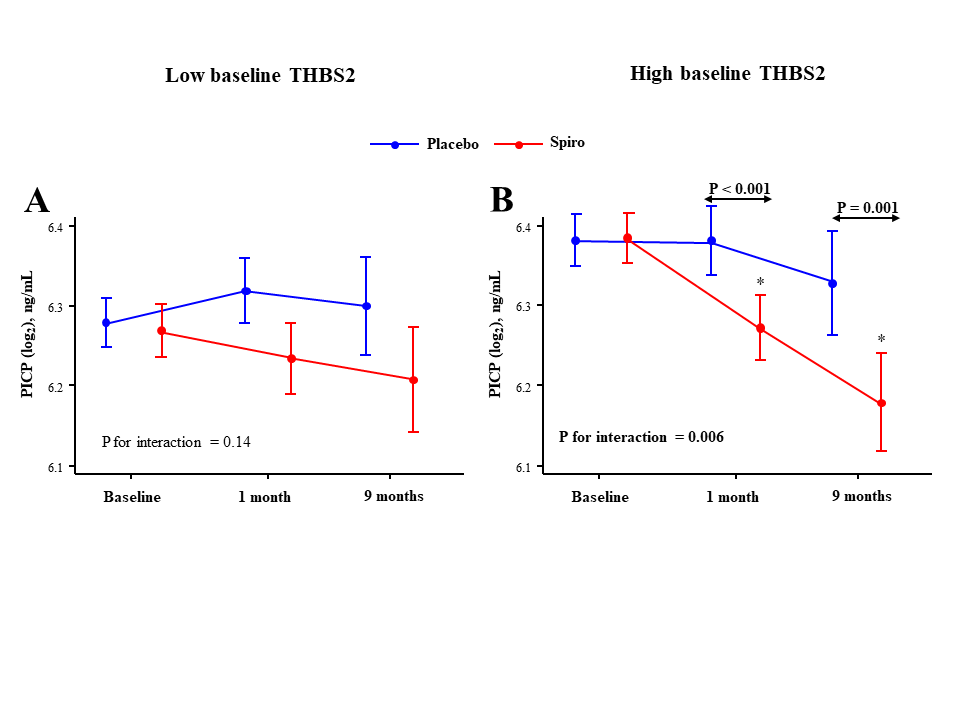


**Supplemental figure 16.** PICP levels according to assigned treatment in patients with low (A: below the median) and high (B: above the median) THBS2 levels at baseline in HOMAGE. Data are expressed as the predicted mean values (estimated marginal means of visits by group) and 95% CI at baseline and at 12 months in patients treated with spironolactone or standard of care after linear mixed regression analysis with PICP as the dependent variable, and treatment, visit, their interaction term, and baseline PICP and estimated glomerular filtration rate fold-change as fixed effects. *P<0.05 vs Baseline.
